# Ethnic inequalities and pathways to care in psychosis in England: a systematic review and meta-analysis

**DOI:** 10.1186/s12916-018-1201-9

**Published:** 2018-12-12

**Authors:** Kristoffer Halvorsrud, James Nazroo, Michaela Otis, Eva Brown Hajdukova, Kamaldeep Bhui

**Affiliations:** 1 Centre for Psychiatry, Wolfson Institute of Preventive Medicine, Barts and the London School of Medicine and Dentistry, Queen Mary University of London, Charterhouse Square, London, EC1M 6BQ UK; 2 Sociology, School of Social Sciences, University of Manchester, Humanities, Bridgeford Street, Oxford Road, Manchester, M13 9PL UK; 3 Centre for Research in Public Health and Community Care (CRIPACC), University of Hertfordshire, Health Research Building, College Lane, Hatfield, AL10 9AB UK

**Keywords:** Pathways to care, Psychosis, Severe mental illness, Ethnicity, Systematic review, Meta-analysis

## Abstract

**Background:**

As part of a national programme to tackle ethnic inequalities, we conducted a systematic review and meta-analysis of research on ethnic inequalities in pathways to care for adults with psychosis living in England and/or Wales.

**Methods:**

Nine databases were searched from inception to 03.07.17 for previous systematic reviews, including forward and backward citation tracking and a PROSPERO search to identify ongoing reviews. We then carried forward relevant primary studies from included reviews (with the latest meta-analyses reporting on research up to 2012), supplemented by a search on 18.10.17 in MEDLINE, Embase, PsycINFO and CINAHL for primary studies between 2012 and 2017 that had not been covered by previous meta-analyses.

**Results:**

Forty studies, all conducted in England, were included for our updated meta-analyses on pathways to care. Relative to the White reference group, elevated rates of civil detentions were found for Black Caribbean (OR = 3.43, 95% CI = 2.68 to 4.40, *n* = 18), Black African (OR = 3.11, 95% CI = 2.40 to 4.02, *n* = 6), and South Asian patients (OR = 1.50, 95% CI 1.07 to 2.12, *n* = 10). Analyses of each Mental Health Act section revealed significantly higher rates for Black people under (civil) Section 2 (OR = 1.53, 95% CI = 1.11 to 2.11, *n* = 3). Rates in repeat admissions were significantly higher than in first admission for South Asian patients (between-group difference *p* < 0.01). Some ethnic groups had more police contact (Black African OR = 3.60, 95% CI = 2.15 to 6.05, *n* = 2; Black Caribbean OR = 2.64, 95% CI = 1.88 to 3.72, *n* = 8) and criminal justice system involvement (Black Caribbean OR = 2.76, 95% CI = 2.02 to 3.78, *n* = 5; Black African OR = 1.92, 95% CI = 1.32 to 2.78, *n* = 3). The White Other patients also showed greater police and criminal justice system involvement than White British patients (OR = 1.49, 95% CI = 1.03 to 2.15, *n* = 4). General practitioner involvement was less likely for Black than the White reference group. No significant variations over time were found across all the main outcomes.

**Conclusions:**

Our updated meta-analyses reveal persisting but not significantly worsening patterns of ethnic inequalities in pathways to psychiatric care, particularly affecting Black groups. This provides a comprehensive evidence base from which to inform policy and practice amidst a prospective Mental Health Act reform.

**Trial registration:**

CRD42017071663

**Electronic supplementary material:**

The online version of this article (10.1186/s12916-018-1201-9) contains supplementary material, which is available to authorized users.

## Introduction

Health inequalities have been a long-standing challenge for global public health systems and the National Health Service (NHS). The Prime Minister’s Race Audit [1] revealed ‘race’ disparities in the fields of education, criminal justice, health and mental health care. In this context, the government recently announced a review of the Mental Health Act (1983, amended in 2007) with a focus on ‘race’ [2]. For patients with severe mental illness, ethnic inequalities in access to and outcomes from mental health services are well known, having been documented for more than four decades [3–7]. The issues that have been investigated include compulsory treatment, criminal justice involvement, police contact and admissions to psychiatric hospitals. All of these are more common in Black patients [3–7]. The explanations for these adverse pathways include multiple social disadvantages that ethnic minority people face, including living in urban environments, poverty, resource-poor services, unemployment and chronic experiences of exclusion, racism and discrimination [8–10], operating at both interpersonal and societal levels [11, 12].

Explanations for ethnic inequalities are often controversial, resulting in scientific disputes about the cause and the remedy of these inequalities. However, what is striking is that the inequalities persist despite periods of increased funding in mental health services, and now the concern is that the inequalities may worsen given the financial crises, continuation of austerity measures and changes to the NHS [13]. There has been little research or policy attention to these ethnic inequalities since the Delivering Race Equality programme [14] ended with evaluations [15] showing no dramatic changes in outcomes. No national policies have been specifically designed to tackle ethnic inequalities in mental health care. In February 2016, the NHS in England’s *Five Year Forward View For Mental Health* recommended a review of the Mental Health Act in response to increasing numbers of detentions that particularly affect Black, Asian, and Minority Ethnic individuals [16]. The current Prime Minister Theresa May has made a pledge to reform the Act [2], although this is occurring in the context of significant shortage of resources, evidence gaps and policy dilemmas on what might be done to remedy ethnic inequalities in mental health care. Routine data collection on admission to and compulsory treatment in psychiatric hospitals by ethnic group was abandoned by the Department of Health in 2011 as these were showing no progress; the latest relevant meta-analyses consider previous literature up to 2012 [6, 17], but there are no recent analyses.

## Methods

We conducted an initial systematic review of reviews mapping the evidence on ethnic inequalities in mental health (with no publication date restrictions), supplemented by up-to-date evidence from a targeted systematic search of primary studies conducted in England and/or Wales of pathways to care between 2012 and 2017. We have followed the PRISMA statement and a protocol detailing methodological considerations of the initial review of reviews was registered with PROSPERO (registration number CRD42017071663).

### Search strategy and screening

A structured search strategy was used (see Additional file 1), influenced by search terms from related systematic reviews [6] [18] [17], with a review-filter adapted for the review of reviews.

Searches for previous reviews were conducted through to 03.07.17 in nine databases: MEDLINE, Embase, PsycINFO, CINAHL, Cochrane Database of Systematic Reviews, Database of Abstracts of Reviews of Effects (DARE), The Campbell Collaboration Online Library, NHS Evidence, and National Institute for Health Research’s (NIHR) Journals Library and Policy Research Programme. King’s Fund reference lists were also searched. We also checked reference lists in included reviews and conducted forward citation searches of references citing the reviews (in Google Scholar), as well as searched PROSPERO for any ongoing reviews (contacting authors regarding publication dates).

We then considered the references in those systematic reviews and meta-analyses that we scored as being of medium or high quality (see AMSTAR quality assessment below), to identify relevant primary studies to carry forward. This was supplemented by an additional search (conducted on 18.10.17) for more recent primary studies published between 2012 and 2017 (as the latest meta-analyses considered research only up to 2012 [6, 17]). We restricted our searches to four databases: MEDLINE, Embase, PsycINFO and CINAHL. We examined both primary studies carried forward from previous medium and high-quality systematic reviews and meta-analyses and those published more recently into the combined updated overall meta-analyses.

Records were screened on title and abstract by two reviewers (KH and EBH), and if necessary, by review of the full text. Where additional information was needed, authors were contacted for original data. A third reviewer (KB) adjudicated if there was disagreement.

### Inclusion and exclusion criteria

#### Study type

All relevant systematic reviews or meta-analyses of the extant research were included in the review of reviews, with no restrictions on methods (i.e. systematic reviews of quantitative, qualitative or mixed methods studies). In the updated search (2012–2017), only primary studies with relevant quantitative data were included to update meta-analyses on pathways to care.

#### Publication type

We included publications in peer-reviewed journals or reports published through recognised platforms such as government or university websites, excluding book chapters or conference papers.

#### Language and region

Only English language publications were retrieved as we included relevant data by ethnicity conducted in England and/or Wales only, as key legislation in the pathways to care such as the Mental Health Act 1983 (amended in 2007) only apply to people in these countries. Although Wales was incorporated in our search and inclusion criteria, all studies included for our meta-analyses were conducted in England as no studies were found that covered Wales.

#### Populations

In the review of reviews, the included population was adults or children with mental disorders as classified by standardised measures (Diagnostic and Statistical Manual or the International Classification of Diseases) or clinical evaluation. To capture the standard outcomes considered in previous pathways to care meta-analyses, we restricted the population to adults with psychoses (affective or non-affective) for our updated meta-analyses.

#### Outcomes

In the review of reviews, outcomes related broadly to prevalence or incidence rates and interventions to tackle ethnic inequalities in addition to pathways to care, but for the present meta-analyses, we only considered the following specific pathways outcomes: compulsory admission or detention, police or criminal justice system involvement, general practitioner (GP) involvement and the duration of untreated psychosis as a potential pathway determinant.

Compulsory admission is the use of Mental Health Act sections to detain persons in ‘secure’ settings, such as a hospital, due to mental health concerns [5]. While ‘forensic detentions’ refer to sections of the Mental Health Act applied to patients who are currently imprisoned or otherwise remanded through the criminal justice system, ‘civil detentions’ refer to sections applied to those who do not currently have these punitive measures imposed upon them.

Police involvement includes police contact on the pathway to care related to the patients’ mental illness, or special measures such as police escort to psychiatric services, with or without patients being sectioned. Criminal justice system involvement refers to contact with any judicial agencies.

General practitioner (GP) involvement in the patients’ pathways to care typically results from referral to GPs from a range of actors such as family members or the patients themselves.

Duration of untreated psychosis is the number of days between the first onset of psychotic symptoms and the beginning of treatment. We excluded data on the duration between the onset of prodromal symptoms (so before actual psychotic symptoms) and the onset of treatment [19].

### Data extraction and quality assessment

A data extraction form was piloted and iteratively amended to improve relevant data capture. One reviewer extracted data for meta-analyses (KH), which was checked by another reviewer (KB). When there was a significant suspected overlap in samples, we selected the paper with the most comprehensive analysis in terms of the specificity or number of ethnic categories, or if similar ethnic categories were applied, we chose relevant data from the largest sample size for use in the respective meta-analyses.

Quality assessment was performed by two independent reviewers by consensus (KH and MO or EBH), with differences to be reconciled by a third reviewer (KB). The AMSTAR checklist was used to assess quality in the review of reviews (see Additional file 2), with reviews classified as either ‘low’ (0–4 points), ‘medium’ (5–8 points) or ‘high’ (9–11 points) quality [20]. Bhui et al.’s assessment tool [3] was subsequently used to assess the primary studies included for meta-analyses, which also allowed studies to be ranked as ‘low’ (0–3 points), ‘moderate’ (4–7 points) or ‘high’ (8–11 points) quality.

### Meta-analyses

Random effects meta-analyses were conducted in Comprehensive Meta-Analysis version 3.3. We extracted raw data by ethnicity, where available, for the denominators and cases to calculate average odds ratios (OR). However, for the duration of untreated psychosis, we calculated average standardised mean differences. Due to its skewed distribution, we either extracted log-transformed means and standard deviations from papers, contacted authors to obtain these data, or used a verified method [21] to transform the raw scales to log-transformed data. Statistical heterogeneity was investigated with the *I*^2^-statistic using guidance of its importance (i.e. above 50% may indicate substantial heterogeneity) [22] and Cochran’s *Q* (with *p* value below 0.05 suggestive of heterogeneity).

The definitions of ethnicity were variously defined and operationalised across papers, with some polarising subjects into a broadly defined Black compared to a broadly defined White group. If disaggregated, ethnic groups comprising White people tended to be divided between White British and White Other, and the Black population variously included Black Caribbean, Black African, Black British or Black Other. The reported Asian population was usually synonymous with a regional South Asian population due to its historical prominence within the Asian community in the UK (with a few exceptions, in which we used specific ‘South Asian’ rather than the aggregated ‘Asian’ data).

For meta-analyses, we summed frequency counts of all disaggregated White and Black groups, respectively, to facilitate comparisons with papers reporting on an aggregated level (White, Black). We conducted (pre-specified) subgroup analyses of more specific ethnic groups where possible. Data had not been sufficiently disaggregated across papers to conduct subgroup analyses at a national level for the South Asian group (e.g. Indian, Pakistani people). Data on the ethnic group simply classified as ‘Other’ across papers were not meta-analysed, as its ethnicity representation varied considerably.

Subgroup analyses for compulsory admission were conducted by patient type, first compulsory admission (either for those experiencing a psychotic episode for the first time or without reference to the patients’ illness stage) compared to those previously admitted who are then readmitted (compulsory) one or more times, and by specific sections of the Mental Health Act. We also conducted separate analyses for involvement of the police or other parts of the criminal justice system. For all the main outcomes, we conducted subgroup analyses to assess any impacts on the findings of the decade that studies had been published (divided into 1980–1989, 1990–1999, 2000–2009 and 2010–2017). We also conducted sensitivity analyses for all main outcomes including only studies of high quality to investigate the potential impact of methodological quality (as pre-specified). To verify the significance of any between-group effects, we report the *p* value for interaction with a *p* value < 0.05 indicating a significant subgroup difference.

## Results

**Fig. 1:**
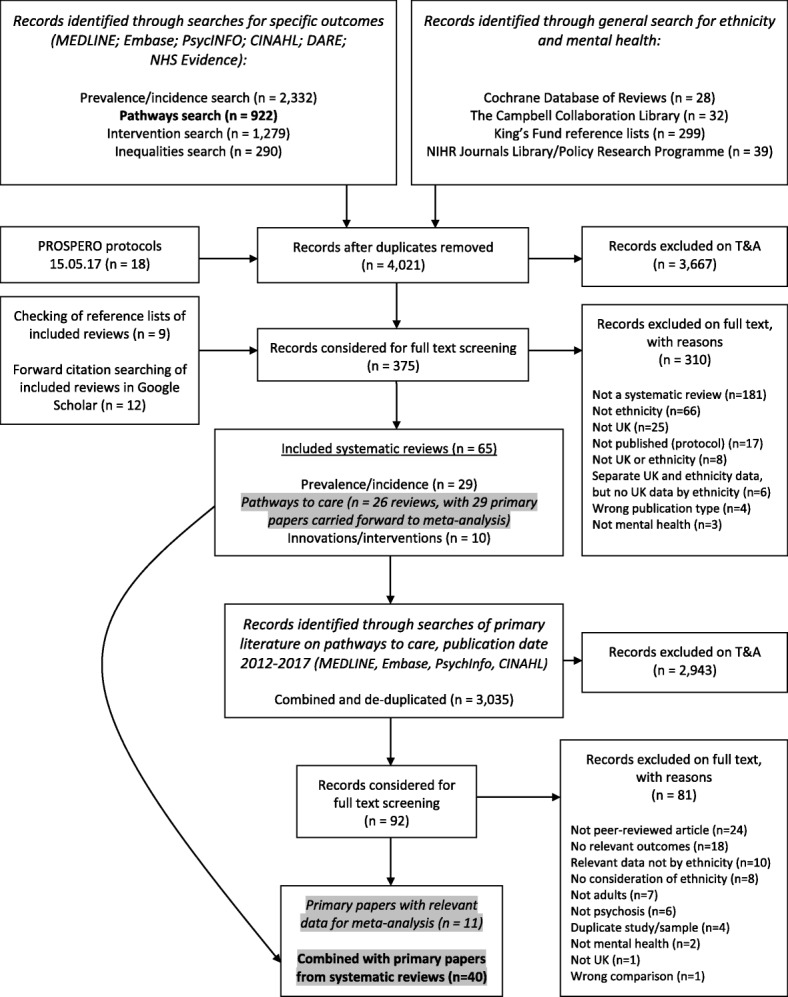
PRISMA flow diagram of searches and screening

Figure 1 shows the PRISMA diagram summarising the search, hits and screening process. Overall, 40 publications provided relevant data to update previous meta-analyses: 29 [19, 23–50] from reference lists of previous reviews or meta-analyses rated as medium [4–6, 8, 10, 12, 17, 51–56] or high [3] quality and 11 [57–67] from the additional search for primary studies between 2012 and 2017. Reasons for excluding publications after full-text review are available in Additional file 3. Table 1 summarises the results of previous meta-analyses, while Table 2 gives an overview of the 40 studies used in our updated meta-analyses (see also Additional file 4 for a more detailed table).

**Table 1:** Previous meta-analyses on pathways to mental health care in the UK

Study	Ethnicity categories	GP, *n*, odds ratio (OR), 95% CI	Civil/forensic detention, *n*, odds ratio (OR), 95% CI	Police or CJS, *n*, odds ratio (OR), 95% CI	DUP	AMSTAR quality (max = 11)
Anderson et al. 2014 [6]	White (reference)	–		–		Total = 7 (Medium quality)
	Black Groups	N = 5 studies, OR = 0.66 (95% CI = 0.53 to 0.82)		Police/CJS: N = 5 studies, OR = 2.14 (95% CI = 1.66 to 2.76)		
	Asian Groups	N = 3 studies, OR = 1.24 (95% CI = 0.81 to 1.91)		Police/CJS: N = 3 studies, OR = 0.73 (95% CI = 0.34 to 1.57)		
Bhui et al. 2003 [3]	White (reference)		–			Total = 9 (High quality)
	Black		Civil: N = 12 studies, OR = 4.31 (95% CI = 3.33 to 5.58)			
Singh et al. 2007 [5]	White (reference)		–			Total = 6 (Medium quality)
	Black		Civil: N = 15 studies, OR = 4.48 (95% CI = 3.71 to 5.41)			
			Forensic: N = 2 studies, OR = 2.45 (95% CI = 1.57 to 3.82)			
	Asian		Civil: N = 4 studies, OR = 3.42 (95% CI = 2.31 to 5.07)			
Singh et al. 2013 [4]	Black vs. Non-Black	N = 4 studies, OR = 0.50 (95% CI = 0.35 to 0.71)	Civil: N = 6 studies, OR = 2.33 (95% CI = 1.85 to 2.93)	Police/CJS: N = 4 studies, OR = 2.25 (95% CI = 1.74 to 2.92)		Total = 5 (Medium quality)
	Black Caribbean vs. White British		Civil: N = 2 studies, OR = 2.88 (95% CI = 1.84 to 4.51)			
	Asian vs. broadly defined White		Civil: N = 2 studies, OR = 0.59 (95% CI = 0.25 to 1.39)

**Table 2:** Overview and quality of included primary studies (used in meta-analyses)

Study	Ethnicity measure	Ethnicity categories (including *n*)	Relevant outcomes	Main findings (ethnic minority vs. White ref.)	Quality* (max = 11)
Ajnakina et al. [57]	Census	WB (62), BA (63), BC (50)	CA (civil), police	Higher CA and police in BA and BC	7 (moderate)
Bebbington et al. [24]	N/A	W (190), BC (49)	CA (civil)	Higher CA in BC	4 (moderate)
Banerjee et al. [23]	N/A	WE (804), BC (375), O (50)	CA (forensic)	Higher CA in BC	2 (low)
Bhui et al. [25]	Place of birth, census	W (184), BA (16), BB(12), BC (26), A/0 (18)	CA (forensic)	Higher CA in BA and BB	8 (high)
Bhui et al. [58]	Self-report	W (177), B (160), SA (114), O (29)	GP, CJS, DUP	Higher CJS in BA and BC, non-significant GP and DUP (including SA)	9 (high)
Bhui et al. [59]	Census	WB (23), WO (14), BA (28), BC (31), BO (1), Bd (4), In (4), P (3), O (14)	CJS	Higher CJS in BA and BC	9 (high)
Birchwood et al. [26]	Third party	WB (74), BC (50), A (30), Ir (5), O (10)	CA (civil), police	Non-significant CA and police (BC and A)	5 (moderate)
Brunet [19]	Third party	W (16), B (36), A (28), O (8)	GP, CA (civil), DUP	Shorter DUP, non-significant GP and CA (B and A)	2 (low)
Burnett et al. [27]	Place of birth	W (38), BC (38), A (24)	GP, CA (mixed), police/CJS	Non-significant GP, CA and police/CJS (B and A)	6 (moderate)
Callan [28]	Place of birth	WB (169), BC (200)	GP, CA (civil), police	Higher CA and police in B, non-significant for GP	7 (moderate)
Cole et al. [29]	Self-report, census	W (39), B (38), A/O (16)	GP, CA (civil), police	Non-significant GP, CA and police (B and A/O)	6 (moderate)
Commander et al. 1999 [30]	Self-report, census	W (40), B (40), A (40)	GP, CA (civil), police	Higher CA and police (B and A), GP higher in A, non-significant for B	4 (moderate)
Crowley and Simmons [31]	Third party	W (75), BC (49), A (28)	CA (civil)	Higher CA in BC, non-significant for A	3 (low)
Davies et al. [32]	Place of birth, census	WB (207), WO (36), BA (27), BC (112), O (15)	CA (mixed)	Higher CA (BA and BC), non-significant in WO	8 (high)
Drake et al. [33]	Self-report	W (216), BC (19), O (13)	DUP	Non-significant DUP for BC	6 (moderate)
Gajwani et al. [60]	Self-report	W (437), BA (62), BC (120), Bd (16), In (47), P (125)	CA (mixed)	Non-significant CA in ethnic minority (including Black) groups	9 (high)
Ghali et al. [61]	Census	WB (183), WO (103), BA (136), BB (152), BC (27), SA (80)	GP, CJS, DUP	Higher CJS in BA only, shorter DUP for Black groups and SA, GP non-significant	11 (high)
Goater et al. [34]	Self-report, census	W (68), B (71), O (15)	CA (mixed)	Higher CA in B after 5 years (non-significant after only 1 year)	6 (moderate)
Harrison et al. [35]	N/A	Non-BC (89), BC (42)	GP, CA (civil), police	Higher CA and police in BC, GP non-significant	4 (moderate)
Ineichen et al. [36]	Third party	WB (193), WO (9), BC (43), O (19)	CA (civil)	Higher CA in BC, non-significant for WO	4 (moderate)
Johnson et al. [37]	N/A	W (173), BA (15), BC (70), O (14)	CA (civil)	Higher CA in BC, non-significant for BA	5 (moderate)
Koffman et al. [38]	Third party	W (2,978), B (631), A (160)	CA (civil)	Higher CA in B and A	5 (moderate)
Lawlor et al. [62]	Census	WB (146), WO (45), BA (41), BC (26), BO (29)	GP, CA (civil), police/CJS	Higher CA and police/CJS (Black groups and WO), lower GP (Black groups) non-significant GP (WO)	10 (high)
Lloyd and Moodley [39]	Third party	W (101), B (37)	CA (civil)	Higher CA in B	5 (moderate)
Mann et al. [63]	Self-report, census	WB (158), WO (93), BA (188), BB (55), BC (78), mixed B/W (36), SA (37), A (O)(29)	GP, CA (civil), CJS	Particularly high point estimate for CA in BA, less marked for other groups and outcomes, or non-significant	9 (high)
McKenzie et al. [40]	Place of birth	WB (58), BC (53)	CA (civil), CJS	Higher CA and CJS in BC	8 (high)
Moodley and Perkins [41]	N/A	W (25), BC (22)	CA (civil)	Higher CA in BC	2 (low)
Moodley and Thornicroft [42]	Third party	W (295), BC (47)	CA (civil), police	Higher CA and police in BC	3 (low)
Morgan et al. [43]	Self-report	WB (237), WO (33), BA (64), BC (128)	GP, CA (civil), CJS	Higher CA, CJS, lower GP (BA and BC), non-significant CA, CJS and GP (WO vs. WB)	9 (high)
Morgan et al. [44]	Self-report	WB (217), BC (129), BA (68)	DUP	Shorter (BA) and non-significant (BC) DUP	8 (high)
Morgan et al. 2017 [64]	Self-report, census	WB (159), BA (44), BC (107)	CA (civil), police	Higher CA and police (BA and BC)	8 (high)
Owens et al. [45]	Third party	Non-BC (155), BC (120)	CA (civil), police	Higher CA and police in BC	4 (moderate)
Parkman et al. [46]	Place of birth, census	WB (94), WO (17), BC (42)	CA (mixed)	Higher CA in BC, non-significant in WO vs. WB	7 (moderate)
Patrick et al. [47]	N/A	W (34), B (26)	CA (civil)	Higher CA in B	3 (low)
Singh et al. [48]	Third party, census	W (352), BC (44)	CA (civil)	Higher CA in BC	8 (high)
Singh et al. [66]	Third party	W (2,587), B (811), A (430), O (359)	CA (civil)	Higher CA in B, non-significant for A	7 (moderate)
Singh et al. [65]	Self-report, census	W (45), B (35), A (43)	CA (civil), CJS, GP, DUP	Higher CA and CJS (B), non-significant GP and DUP (B), non-significant CA, CJS, DUP (A)	7 (moderate)
Takei et al. [49]	N/A	W (49), BC (32)	CA (civil)	Higher CA in B	5 (moderate)
Thomas et al. [50]	Third party	W (1,265), BC (193), A (76)	CA (civil), police	Higher CA (BC and A), higher police (BC), non-significant police (A)	3 (low)
Weich et al. [67]	N/A	W (997,169), B (39,249), A (46,544), mixed (13,781), O (22,053)	CA (civil)	Higher CA in B and A	2 (low)

Ethnicity categories: *W* White, *WB* White British, *WO* White Other, *Ir* Irish, *B* Black, *BA* Black African, *BB* Black British, *BC* Black Caribbean, *BO* Black Other, *A* Asian, *SA* South Asian, *Bd* Bangladeshi, *In* Indian, *P* Pakistani, *O* Other

Relevant outcomes: *CA* compulsory admission (for civil, forensic or mixed (civil and forensic) detentions), *CJS* criminal justice system involvement, *GP* general practitioner involvement, *DUP* duration of untreated psychosis

*The scoring system used to rate primary studies is replicated from Bhui et al. [3]. From a maximum of 11 points, primary studies that received a total of 0–3 points were ranked as ‘low’ quality, 4–7 points ‘moderate’ quality and 8–11 points ‘high’ quality. See Additional file 4 for the full breakdown of the score of each item of the quality assessment system for the respective studies

### Compulsory admission

**Fig. 2:**
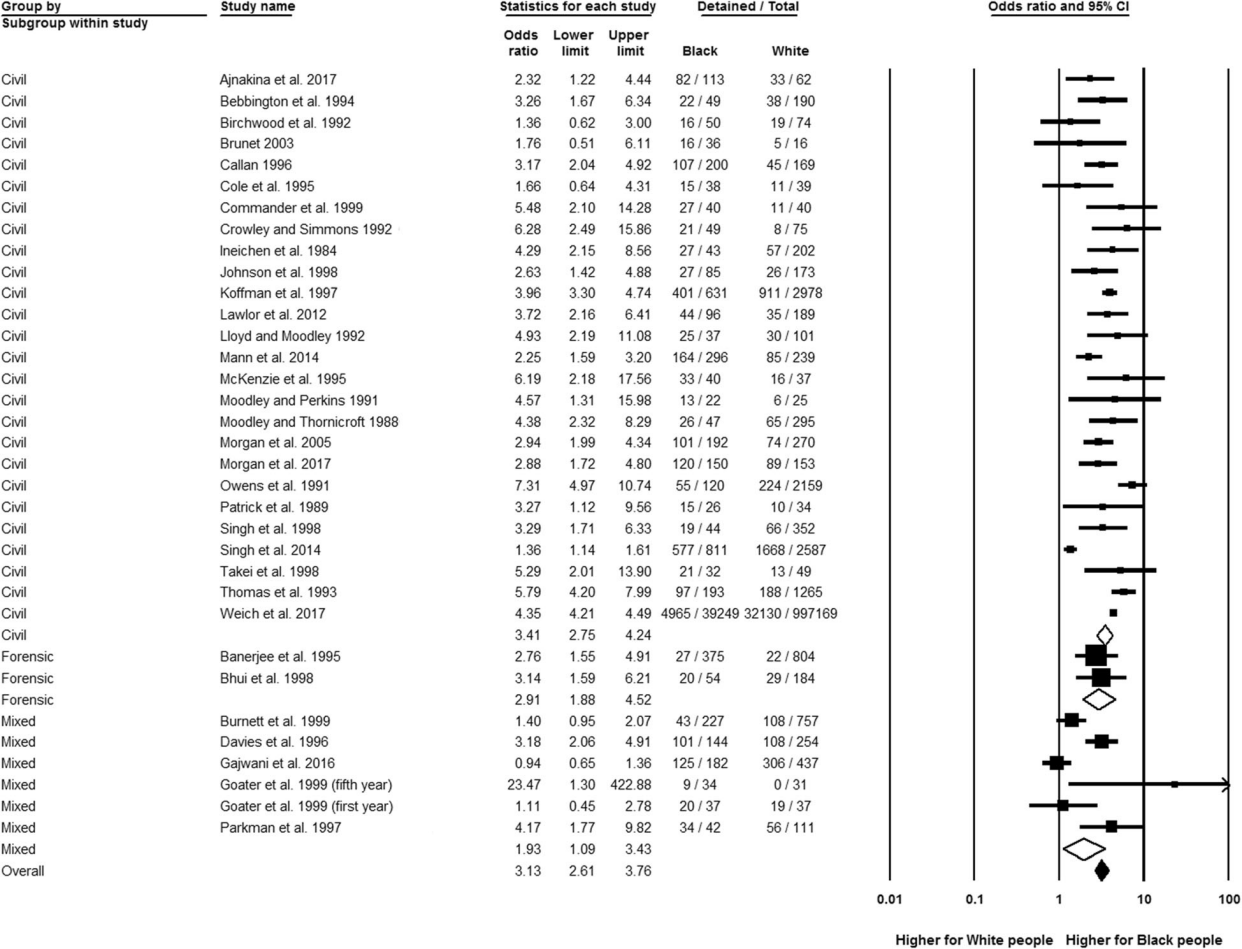
Compulsory admission by patient type, Black relative to White group. Two effect estimates were entered from Goater et al. [34] as data were recorded after the first and the fifth year for that study

Figure 2 shows that Black people (broadly defined) had significantly higher odds of compulsory admission than the White reference group (OR 3.13, 95% CI 2.61 to 3.76, *n* = 33), with a high possibility of statistical heterogeneity (*I*^2^ = 89.84%; *Q* = 324.73, df = 33, *p* < 0.01).

In subgroup analyses, Black people were almost three and a half times more likely to be compulsory admitted than White people in civil detentions (OR 3.41, 95% CI 2.75 to 4.24, *n* = 26) while a little short of three times higher in forensic detentions (OR 2.91, 95% CI 1.88 to 4.52, *n* = 2), but the between-group analysis was not significant (*p* for interaction = 0.53). No significant subgroup differences by time (in the form of the decade of publication) were revealed. Available evidence on specific sections of the Mental Health Act showed that Black people (all admitted) were more likely to be detained under (civil) Section 2 for the assessment of patients over 28 days than White people (OR 1.53, 95% CI 1.11 to 2.11, *n* = 3), with non-significant results yielded for all other specific civil and forensic and police sections (see Additional file 5 for section definitions). Analyses of first compulsory admission compared to readmissions indicated no significant subgroup differences. Compared to the White reference group, there were elevated rates of civil detentions for Black Caribbean (OR 3.43, 95% CI 2.68 to 4.40, *n* = 18), Black African (OR 3.11, 95% CI 2.40 to 4.02, *n* = 6) and Black British people (OR 2.04, 95% CI 1.11 to 3.75, *n* = 1); this was also the case for forensic detentions for the Black ethnic groups (Black British OR 7.48, 95% CI 2.22 to 25.20, *n* = 1; Black African OR 3.21, 95% CI 1.08 to 9.51, *n* = 1; Black Caribbean OR 2.52, 95% CI 1.54 to 4.13, *n* = 2). The between-group variations were not significant.

The sensitivity analysis of only high-quality studies (the majority from 2012 to 2017, see Table 2) revealed no significant between-group differences in effects, compared with the overall analyses.

**Fig. 3:**
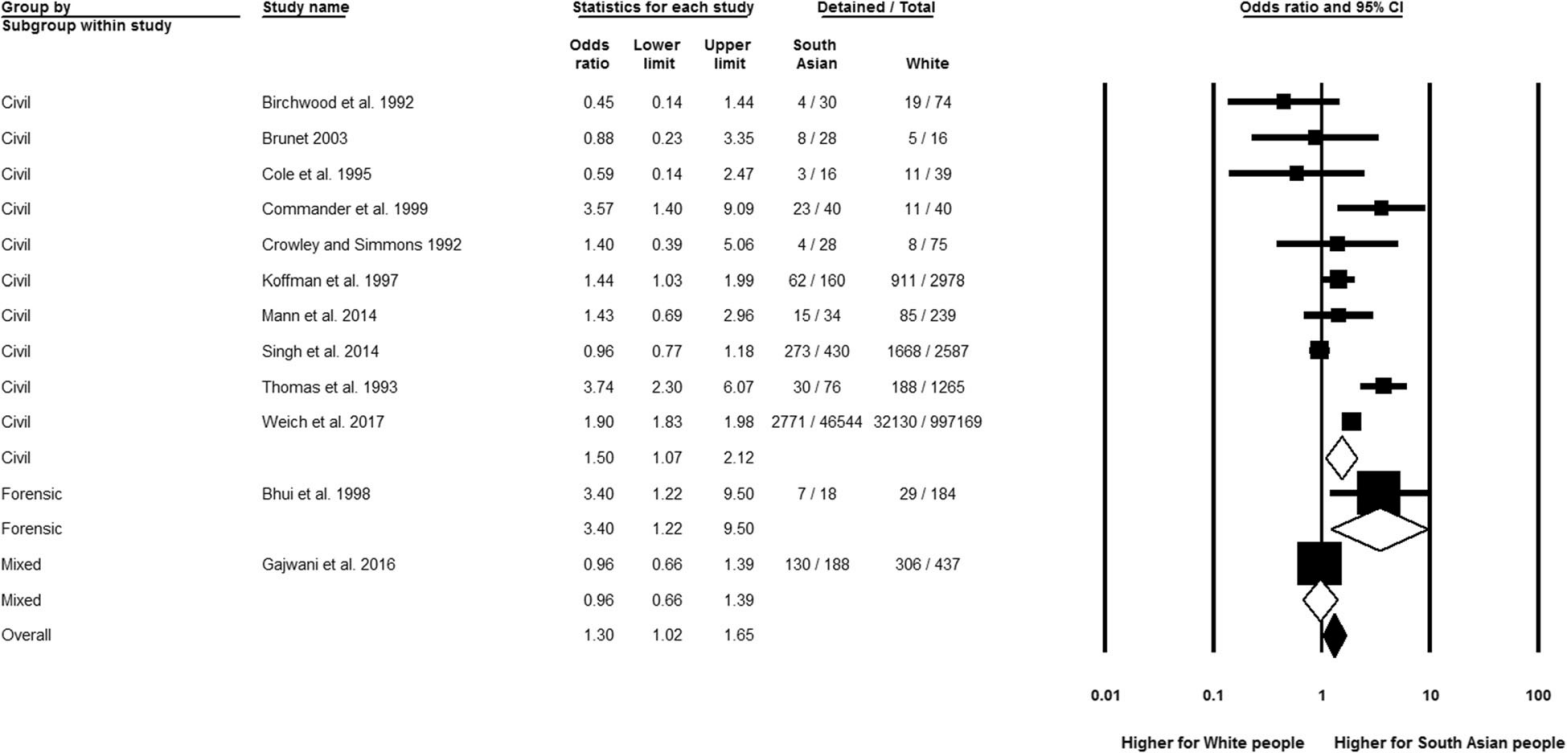
Compulsory admission by patient type, South Asian relative to White group

Figure 3 shows that the South Asian group had significantly higher odds of compulsory admission than the White group (OR 1.30, 95% CI 1.02 to 1.65, *n* = 12), with a high possibility of statistical heterogeneity (*I*^2^ = 85.19%; *Q* = 74.28, df = 11, *p* < 0.01). Disaggregation by patient type revealed significant estimates for both forensic (OR 3.40, 95% CI 1.22 to 9.50, *n* = 1) and civil detentions (OR 1.50, 95% CI 1.07 to 2.12, *n* = 10) in the South Asian population, with a non-significant subgroup difference (*p* = 0.14). Also, no significant subgroup differences were revealed when publication decade was considered. However, the significant result for civil detentions was rendered non-significant in sensitivity analysis by study quality (OR 1.43, 95% CI 0.69 to 2.96), but based on only one study [63] and a non-significant *p* value for interaction with the overall analysis (*p* = 0.90). It was not possible to establish any significant variations on specific sections of the Mental Health Act for all admitted South Asian compared to all admitted White people. Analyses of admission frequency for civil detentions, when compared to the White reference, revealed a significant difference (*p* < 0.01) suggesting a higher rate of recurrent admissions (OR 4.75, 95% CI 2.64 to 8.54, *n* = 1), than first compulsory admission (OR 1.19, 95% CI 0.72 to 1.98, *n* = 6) for South Asian people.

A comparison of the White Other with the White British group indicated towards higher odds of compulsory admission for the former group, but was not significant (OR 1.51, 95% CI 0.99 to 2.30, *n* = 6). There was relatively little reason to suspect statistical heterogeneity (*I*^2^ = 25.25%; *Q* = 6.69, df = 5, *p* = 0.25). Furthermore, a non-significant result was observed in the separate analysis for civil detentions (OR 1.56, 95% CI 0.85 to 2.87, *n* = 4) and when only high-quality studies were included. No significant subgroup differences to help explain the overall results were detected in analyses by publication decade. It was not possible to investigate estimates for White Other people for forensic detentions, by specific Mental Health Act sections, or by admission frequency due to a lack of available data.

### Police or criminal justice system involvement

**Fig. 4:**
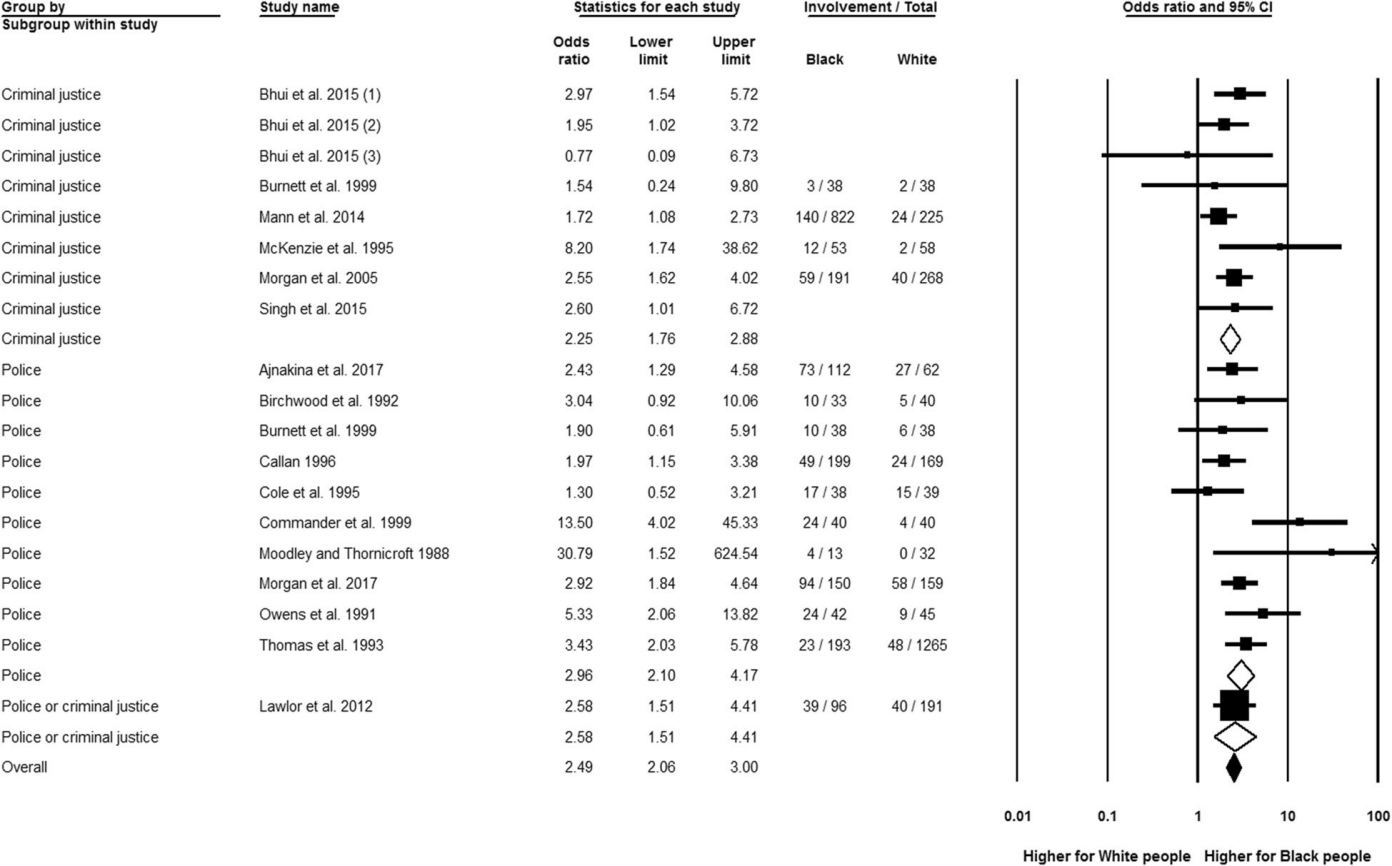
Police or criminal justice system involvement, Black relative to White group. Three effect estimates were entered from Bhui et al. [25], as these statistics related to different Black groups in that study: 1 = Black Caribbean, 2 = Black African, 3 = Black Other

Figure 4 shows that Black people had almost two and a half higher likelihood of a combined estimate of police and criminal justice system involvement than the White reference (OR 2.49, 95% CI 2.06 to 3.00, *n* = 17), with relatively low potential for heterogeneity (*I*^2^ = 26.44%; *Q* = 24.47, df = 18, *p* = 0.14). Separate analyses by police contact (OR 2.96, 95% CI 2.10 to 4.17, *n* = 10) and criminal justice system involvement (OR 2.25, 95% CI 1.76 to 2.88, *n* = 6) both demonstrate raised rates for Black people. Considering publication decade or including only high-quality studies made no difference to these findings.

Moreover, no significant subgroup-effect variance was detected between the analyses by specific Black groups. These analyses showed that, compared to the White reference, the Black African (OR 3.60, 95% CI 2.15 to 6.05, *n* = 2) and Black Caribbean populations (OR 2.64, 95% CI 1.88 to 3.72, *n* = 8) had a higher probability of police contact. Significantly higher probability of criminal justice system involvement was also identified for the Black Caribbean (OR 2.76, 95% CI 2.02 to 3.78, *n* = 5) and Black African populations (OR 1.92, 95% CI 1.32 to 2.78, *n* = 3), while the result for Black British people did not reach significance (OR 1.56, 95% CI 0.98 to 2.48, *n* = 1).

**Fig. 5:**
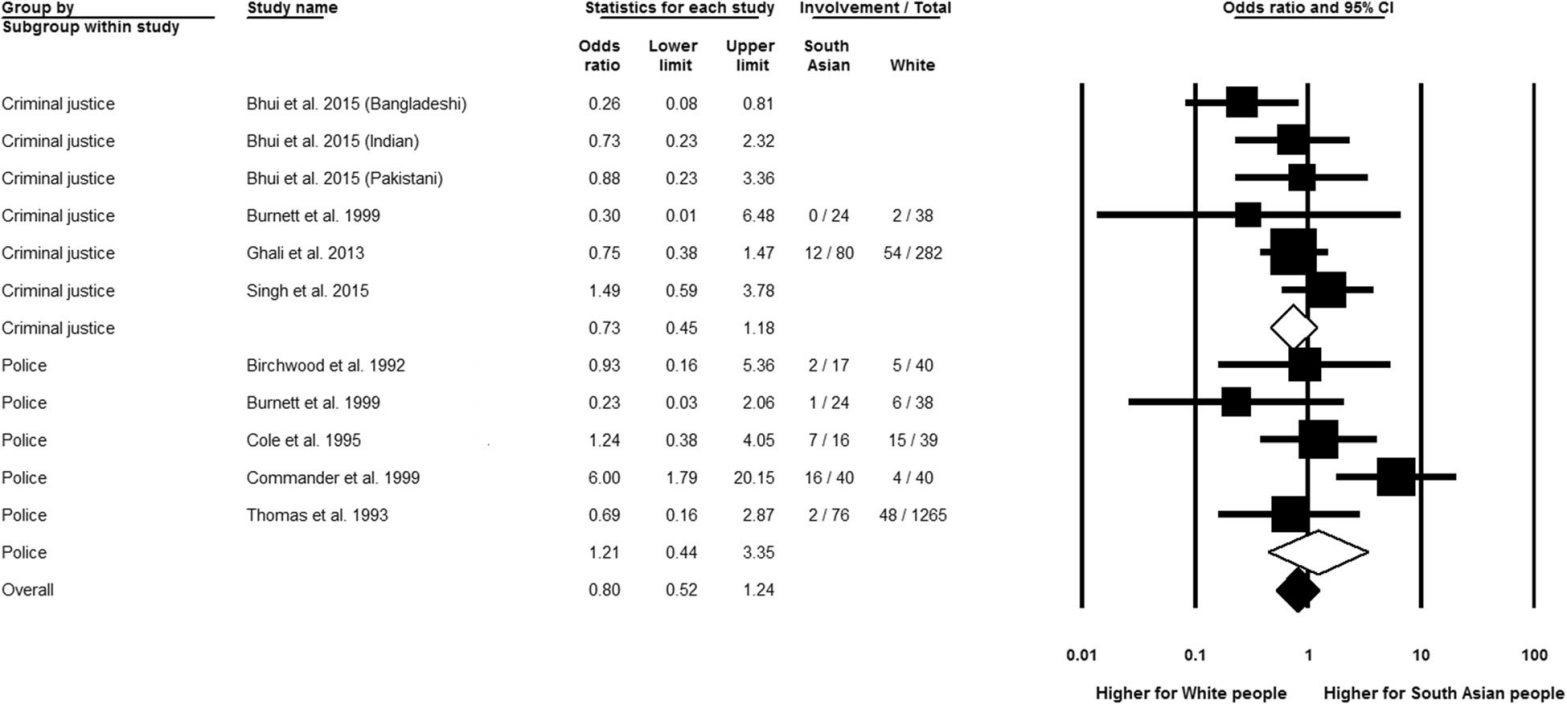
Police or criminal justice system involvement, South Asian relative to White group. Three effect estimates were entered from Bhui et al. [25], as these statistics related to different South Asian nationalities in that study: Bangladeshi, Indian and Pakistani

South Asian people, compared to the White reference, did not show a significant difference in police and criminal justice system involvement (see Fig. 5, OR 0.80, 95% CI 0.52 to 1.24, *n* = 9), with the threshold for ‘substantial heterogeneity’ not reached (*I*^2^ = 44.28%, *Q* = 17.95, df = 10, *p* = 0.06). Separate meta-analyses for police (OR 1.21, 95% CI 0.44 to 3.35, *n* = 5) and criminal justice system involvement (OR 0.73, 95% CI 0.45 to 1.18, *n* = 4) both yielded non-significant results, as did sensitivity analysis using only high quality studies. Time was not a significant source of heterogeneity in the analyses by different decades of publication.

Comparing the White Other and White British groups revealed a significantly higher risk in the combined analysis of both police and criminal justice system involvement for the White Other group (OR 1.49, 95% CI 1.03 to 2.15, *n* = 4). All studies were of high quality, with the *I*^2^-statistic (=0%) and Cochran’s *Q* (=2.60, df = 3, *p* = 0.46) not detecting heterogeneity. It was not possible to analyse the separate effects of police involvement as the study [68] including this form of involvement combined it with criminal justice system data, while the analysis of the remaining three studies that assessed criminal justice system involvement independently yielded a non-significant result (OR 1.28, 95% CI 0.84 to 1.95, *n* = 3). Time was not a significant heterogeneity source.

### General practitioner (GP) involvement

**Fig. 6:**
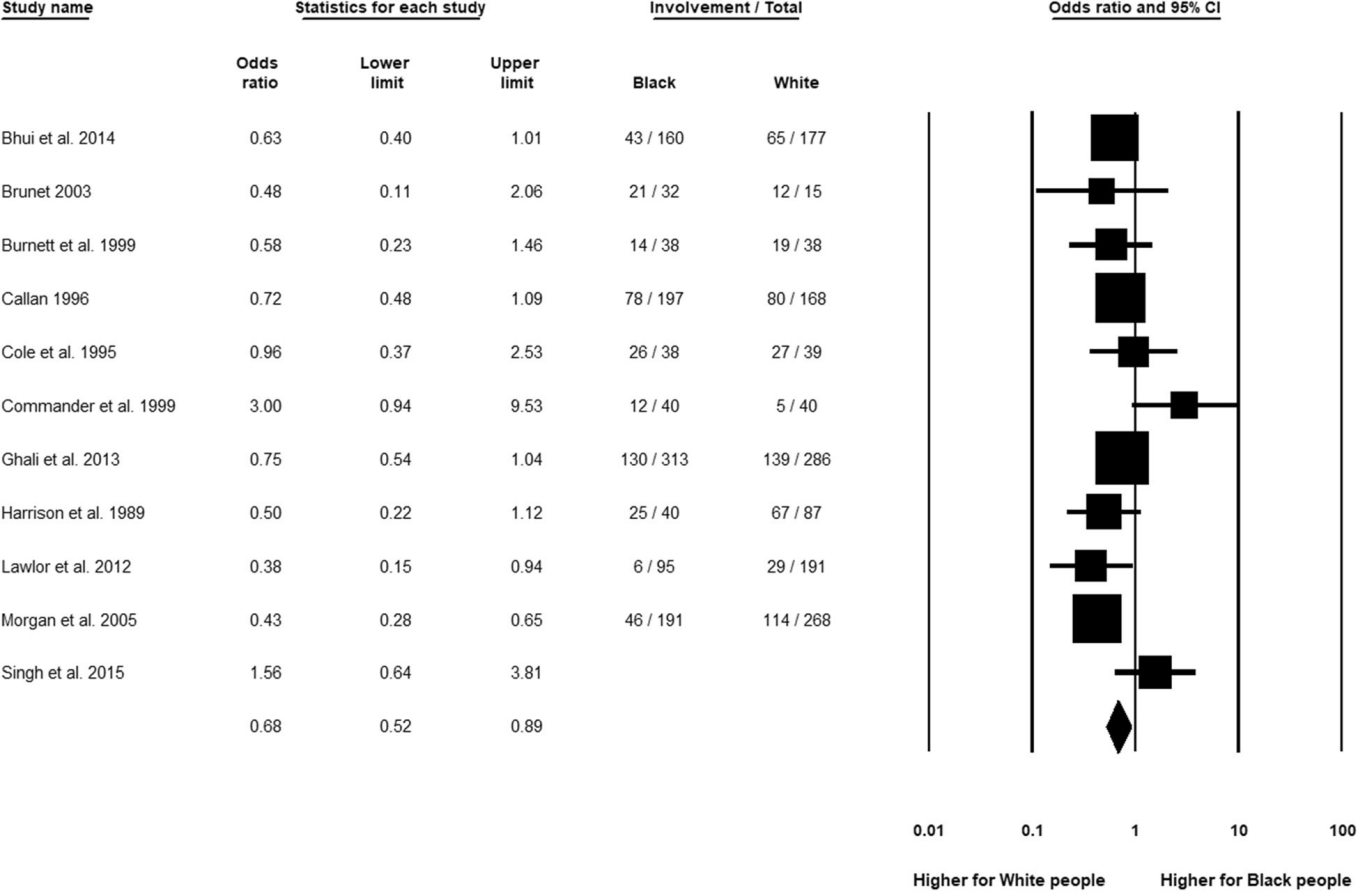
General practitioner (GP) involvement, Black relative to White group

Figure 6 shows that GP contact was significantly less likely for Black compared to White people (OR 0.68, 95% CI 0.52 to 0.89, *n* = 11). The *I*^2^-statistics did not reach the threshold for ‘substantial heterogeneity’ at 50% (*I*^2^ = 44.40%, *Q* = 17.99, df = 10, *p* = 0.06). There were also no significant differences between the subgroup analyses by publication decade. Moreover, the significance of the lower GP contact for Black than White patients was retained when only high-quality studies were analysed separately.

Analyses by specific ethnic group revealed only minor and non-significant (*p* = 0.71) differences in effect magnitude of GP involvement between Black Caribbean (OR 0.59, 95% CI 0.46 to 0.75, *n* = 6) and Black African people (OR 0.52, 95% CI 0.37 to 0.73, *n* = 3), compared to the White group. In the Black British population, the result was non-significant (OR 0.77, 95% CI 0.51 to 1.14, *n* = 1).

**Fig. 7:**
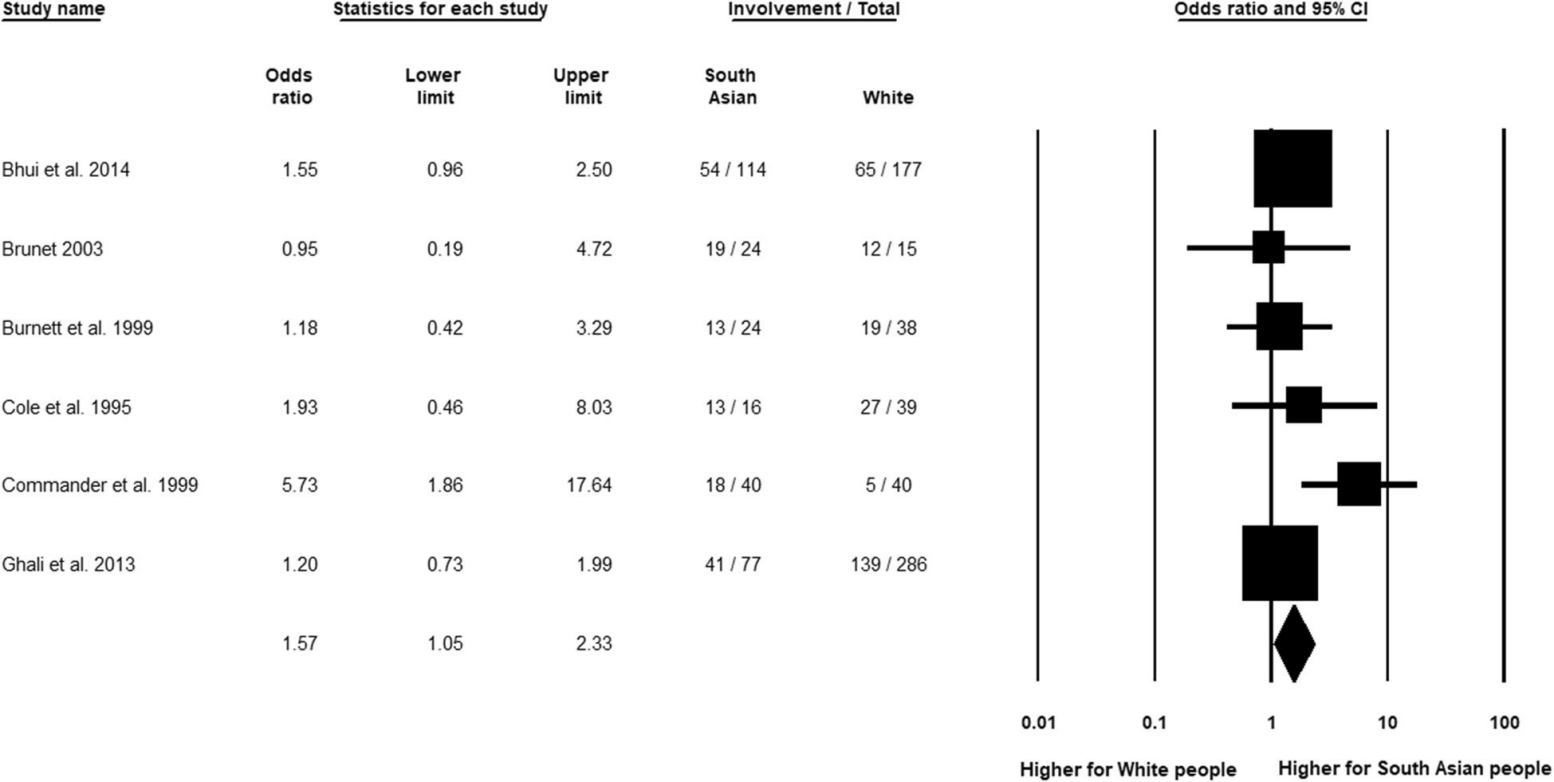
General practitioner (GP) involvement, South Asian relative to White group

The meta-analysis of South Asian people compared to White people showed a reverse relationship in which the ethnic minority group (South Asian people) had significantly higher GP involvement (Fig. 7, OR 1.57, 95% CI 1.05 to 2.33, *n* = 6). Overall heterogeneity was not indicated (*I*^2^ = 12.23%; *Q* = 6.84, df = 6, *p* = 0.34), with time not being an explanation of the overall finding in the analyses by publication decade. In the analysis of high-quality studies, however, the significance of the overall finding was lost (OR 1.38, 95% CI 0.97 to 1.95, *n* = 2).

Finally, there is a statistically non-significant indication towards lower GP involvement for White Other compared to White British people (OR 0.74, 95% CI 0.45 to 1.20, *n* = 3). All studies were rated as being of high quality, with no significant subgroup differences by publication decade and with the thresholds for statistical heterogeneity not reached (*I*^2^ = 31.74%; *Q* = 2.93, df = 2, *p* = 0.23).

### Duration of untreated psychosis

**Fig. 8:**
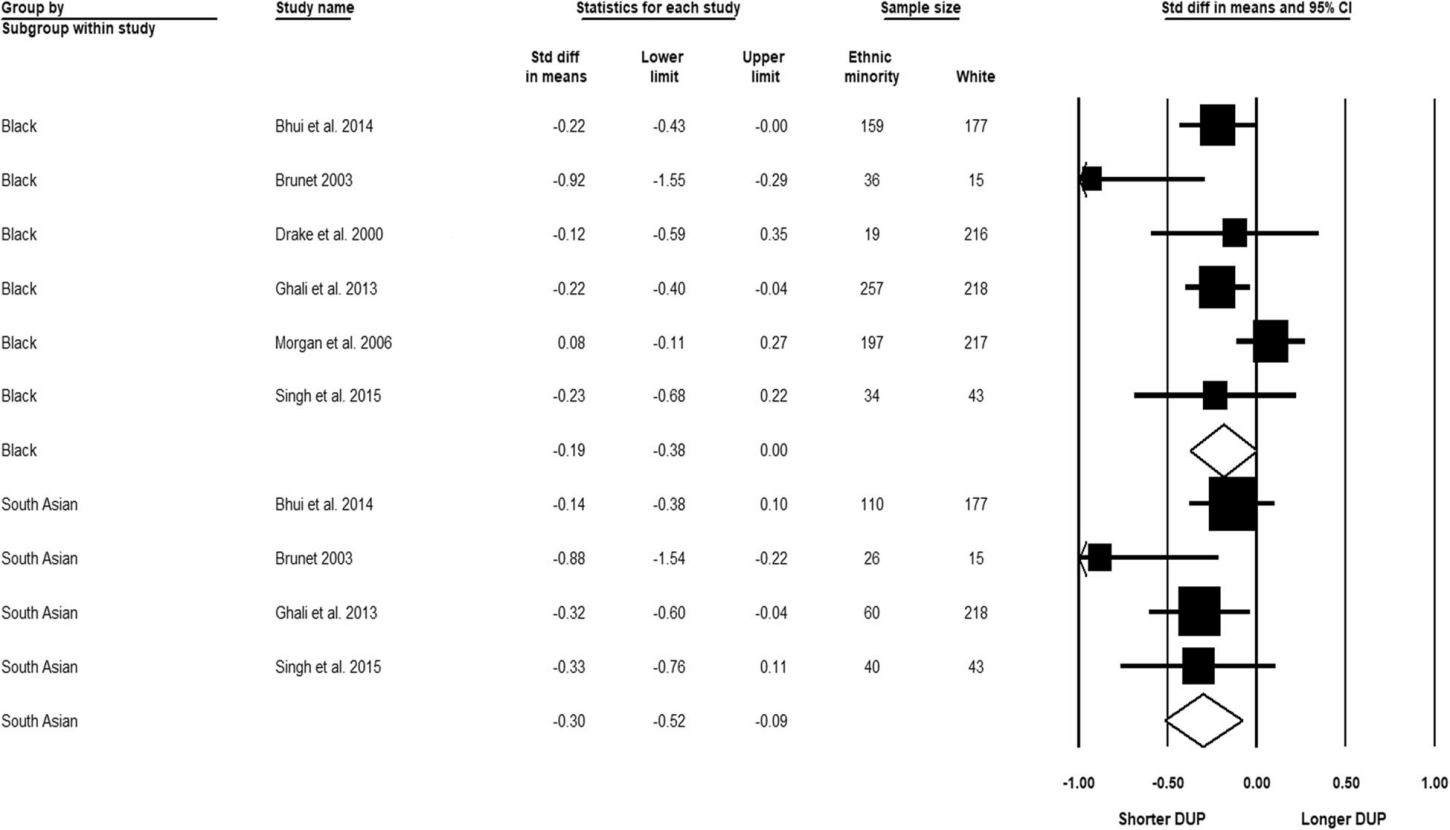
Duration of untreated psychosis (DUP), Black and South Asian relative to White group

Figure 8 shows the meta-analysis of the duration of untreated psychosis by the broadly defined Black and the South Asian groups, respectively, relative to the White reference. A non-significant result was indicated in the comparison between Black and White people based on six studies (SMD − 0.19, 95% CI − 0.38 to 0.00). There was reason to suspect heterogeneity of substantial importance (*I*^2^ = 60.16%; *Q* = 12.55, df = 5.00, *p* = 0.03). Time was not a significant heterogeneity source, and the result remained non-significant when only high-quality studies were retained. The second comparison indicated a significantly shorter duration for South Asian compared to White people based on four studies (SMD − 0.30, 95% CI − 0.52 to − 0.09), with substantial heterogeneity not detected (*I*^2^ = 35.07%; *Q* = 4.62, df = 3.00, *p* = 0.20) and no significant differences between the subgroup analyses by publication decade. In addition, the significance of the overall result was retained with only high-quality studies included. It was not feasible to conduct subgroup analyses by more specific Black, South Asian or White groups due to limited available data and insufficient reporting.

## Discussion

### Summary of findings

Our meta-analyses incorporate decades of primary research and synthesise the up-to-date evidence on ethnic inequalities in the pathways to care in psychosis and the duration of untreated psychosis as a potential pathway determinant. In previous literature, GP consultation has been considered less coercive than alternative pathways, with disengagement from services associated with police and criminal justice system involvement [68], while service dissatisfaction [69] and post-traumatic stress [70] have been associated with compulsory admission. Compared to the White reference, our analyses show excess rates for Black African and Black Caribbean people in compulsory admission, police or criminal justice system contact, but low probability of GP involvement. Insufficient evidence was identified for the Black British population for some outcomes or results were non-significant, apart from the significantly higher rates in compulsory admission (civil and forensic). Although point estimates tended to be less elevated, significantly higher rates were also found for other ethnic minority groups: in detention (particularly repeat) for South Asian people and police and criminal justice system involvement for White Other than White British people. In their comparisons with the White reference, there was a relative lack of variations in the duration of untreated psychosis for Black people and shorter times to treatment for South Asian people. Analyses by decade of publication across all the main outcomes above suggested that the results have not undergone significant changes over time.

### Comparison with previous meta-analyses

The present meta-analyses benefit from combining all relevant studies from previous meta-analyses and updating the evidence base. Variations from previous meta-analyses (see Table 1) are mainly observed in the magnitude rather than direction of effects, and with overlapping confidence intervals. This suggests that concerns that inequalities may worsen in the wake of the financial climate and austerity have not materialised in significantly increased inequalities. For instance, for civil detentions, we show a slightly lower (but significantly raised) point estimate for Black compared to White people (OR 3.41, 95% CI 2.75 to 4.24, *n* = 26) than in meta-analyses from 2007 [5] (OR 4.48, 95% CI 3.71 to 5.41, *n* = 15) and 2003 [3] (OR 4.31, 95% CIs 3.33 to 5.58, *n* = 12).

Our analyses are also unique in investigating sections of the Mental Health Act and find significantly higher rates of compulsory admission in the Black compared to the White population for (civil) Section 2 only, contradicting previous research [71] suggesting a particular relevance of police sections (e.g. Section 136). Former meta-analyses also demonstrate highly variable odds of civil detentions in the Asian population, with a non-significant estimate from 2013 [4] (OR 0.59, 95% CI 0.25 to 1.39, *n* = 2) but significantly higher rates from 2007 [5] (OR 3.42, 95% CI 2.31 to 5.07, *n* = 4)—the latter significant finding is also indicated in our analyses but with a smaller effect size (OR 1.50, 95% CI 1.07 to 2.12, *n* = 10). For police and criminal justice system involvement, we show a highpoint estimate in the Black versus White group analysis of all cases of psychosis (OR 2.49, 95% CI 2.06 to 3.00, *n* = 17), similar to meta-analyses of first episode psychosis from 2014 [6] (OR 2.14, 95% CI 1.66 to 2.76, *n* = 5) and from 2013 [4] (OR 2.25, 95% CI 1.74 to 2.92, *n* = 4). Only minor differences in point estimates and overlapping confidence intervals between previous meta-analyses of GP involvement and our meta-analyses are indicated (see Table 1). The failure of previous meta-analyses to analyse more specific ethnic group variations across all the main pathways to care outcomes, to distinguish between police and criminal justice system involvement or to break down the evidence on the duration of untreated psychosis by UK country level [17] (see also Table 1) precludes further comparisons.

### Strengths and limitations

Our review approach allowed us to present policy-relevant information [72] in the context of informing an urgently needed reform of the Mental Health Act, and sustained efforts globally to understand ethnic inequalities in mental health experiences and outcomes. Carrying forward primary studies from previous reviews is a previously adopted technique [4]; however, the selection of reviews to help identify relevant primary studies for meta-analyses is usually not conducted in a systematic fashion [4]. Our initial review of reviews comprehensively mapped the availability of previous systematic reviews and meta-analyses, and searches were implemented in multiple rounds to capture an extensive range of evidence, though further relevant literature may be available through the ‘grey literature’ (i.e. book chapters, conference papers). It is also acknowledged that previous reviews and meta-analyses are of variable quality. As such, we used the AMSTAR assessment tool to select primary studies only from reviews or meta-analyses ranked of sufficient quality (medium or high). This mitigated the potential limitation of relying on the robustness of the methods and searches of the identified reviews and meta-analyses that studies were carried forward from.

### Implications for research

Most research investigating inequalities in service uptake focuses on dimensions of inequality other than ethnicity, and mental illnesses other than psychotic disorders are rarely investigated [18]. Both ethnic group and moderating variables also need to be reported in a more consistent fashion. Although our subgroup analyses included patient type, admission frequency, sections of the Mental Health Act and sensitivity analyses to assess the impact of methodological quality, inconsistency in the analyses or reporting across the available primary studies of only some moderating variables and for only some ethnic groups leaves open the possibility that unexplored variables may work alongside other relevant factors (detected or not) to explain ethnic variations. This has rendered further subgroup analyses (e.g. age, gender, socioeconomic influences) less fruitful against our aim and the backdrop of updating the evidence base on ethnic inequalities, in which we have included both aggregated analyses of Black and White populations to enable statistically more powerful analyses, as well as extended the relevant literature by exploring variations by more specific ethnic categories (Black Caribbean, Black African, Black British, South Asian, White Other and White British). Yet, of note is the noticeably high *I*^2^-statistic for our analyses of compulsory admission in particular (above 80%). Anderson et al. [17] observed considerable shaping of compulsory admission outcomes by socioeconomic variables—i.e. in one study [27] higher compulsory admission for Black males living alone, Asian patients living in public housing, and White males with low education—discouraging them from conducting meta-analyses on compulsory admission. Various practices exist of whether it is informative to conduct meta-analyses when confronted with high levels of statistical heterogeneity [73]. However, we believe that the consistently high rates for compulsory admission that have been reported over many decades for ethnic minority people [3–5, 23–25, 28, 30–32, 34, 36–43, 45–50, 57, 62–64, 66, 67], particularly for Black groups, need to be highlighted and considered in the planning of any prospective Mental Health Act reform, while not disregarding but alerting readers and decision makers to the potential for heterogeneity when inspecting forest plots of average effects. The detected high levels of heterogeneity in previous meta-analyses of pathways to mental health care have influenced our decision to choose random effects models to acknowledge such heterogeneity. However, the range of potential moderating factors will need further investigation and, towards this end, will require sufficient reporting in future primary study papers to determine their respective roles.

### Implications for policy and practice

Despite the limitations of the available literature, our meta-analyses provide the most inclusive and up-to-date—as far as we are aware—evidence base on ethnic inequalities in treatment for severe mental illness in England. In the context of a prospective reform of the Mental Health Act, this provides—alongside other relevant sources of information—a foundation from which key issues can be mapped out to increase awareness and inform policy and practice. The apparent lack of focus on tackling the persisting ethnic inequalities in mental health is surprising, especially in the wake of policymakers’ emerging focus on general mental health issues [2]. A reconfiguration of services including more wide-spanning, national policies are needed in order to address these inequalities in mental health care, in addition to inequalities in health more broadly. A prospective policy and practice shift should not only concern ethnic inequalities, but also other and associated inequalities centred around socioeconomic and geographical factors, gender, age, and so on—without preventing initiatives from accommodating for the specific needs and priorities of ethnic minority people. Furthermore, these lessons will be transferable to tackling inequalities in health and mental health in other national contexts.

Policymakers and practitioners will need to consider how ethnic variations in pathways to mental health care reflect societal, institutional and interpersonal disadvantages, including racism at each of these levels. Institutional racism often receives less attention than more overt incidents of racial prejudice and racial violence, and some critical voices have even denied the relevance of ‘race’ and racism [74]. However, it is important to recognise how racism operates within and across societal institutions and acts as a fundamental mechanism driving and sustaining inequalities. Racism reflects power dynamics in broader society that are embedded in mainstream institutions over time, shaped by the historical and contemporary inequalities in access to social, cultural and economic resources by racial or ethnic background [75, 76].

A limited number of relevant programmes championing reform and ‘race equality’ in the NHS, such as the 2005 Delivering Race Equality programme, have contributed to learning about barriers and facilitators to service access, but they have done little to achieving wider system changes or to reduce ethnic inequalities in detention rates [15]. More recently, the Prime Minister’s Race Disparity Audit highlighted broader ethnic inequalities, for example in relation to education, the labour market and housing [1]. However, it did not examine how these inequalities can be intensified in times of economic recession and through hostile political ideologies. A recent report showed the particularly adverse effects of the extensive cuts to welfare benefits and health and other services that have occurred since 2010, on the lives of disadvantaged ethnic minority communities [77]. Although our analyses showed persisting, but no significantly worsening inequalities in pathways to mental health care, the more prolonged manifestations or ramifications of the current political climate may be yet to be realised.

Perhaps the main challenge for services is how to identify and tackle institutional racism that is entrenched in the practice and principles of institutions—including their regulations, protocols, cultures and role definitions—and reinforced by stakeholders trained to behave in a compliant manner. Practitioners (mental health, social care, criminal justice) are likely to have internalised the expectations of how to operate within their institutions to such an extent that they unwittingly perform their duties without fully considering how they might sustain inequalities [75, 76].

## Conclusions

Evidence on pathways to mental health care has been presented through updated meta-analyses that reveal persisting inequalities in service use and referral methods for severe mental illness that adversely affect ethnic minority people in England. This is demonstrated for Black ethnic groups in particular with greater compulsory admission and police or criminal justice system contact, rather than seemingly more enabling channels such as GP consultation. We urge decision makers to consider these findings in the planning of prospective mental health reforms and the reconfiguration of services.

## Additional files

Additional file 1: Sample search strategies through Ovid MEDLINE(R). (DOCX 33 kb)

Additional file 2: AMSTAR quality assessment (for review of reviews). (DOCX 39 kb)

Additional file 3: Reasons for exclusion on full text. (DOCX 93 kb)

Additional file 4: Full summary and quality scores of included primary studies (used in meta-analyses). (DOCX 51 kb)

Additional file 5: Relevant sections of the Mental Health Act (1983, amended in 2007). (DOCX 22 kb)

## Abbreviations

CI: Confidence intervals
DARE: Database of Abstracts of Reviews of Effects
GP: General practitioner
NHS: National Health Service
NIHR: National Institute for Health Research
OR: Odds ratio
SMD: Standardised mean difference
