# Supplementary material for: Ethnic inequalities and pathways to care in psychosis in England: a systematic review and meta-analysis
Source: BMC Med. 2018 Dec 12;16:223. doi: 10.1186/s12916-018-1201-9 (PMC6290527; doi:10.1186/s12916-018-1201-9)
Supplement: Supplementary file 1 — Sample search strategies through Ovid MEDLINE(R). (DOCX 33 kb) [file 12916_2018_1201_MOESM1_ESM.docx]

**Additional file 1:**

**Sample search strategies through Ovid MEDLINE(R)**

General search terms (combined, respectively, with more specific terms in subheadings below)

1. exp Mental Disorders/

2. Mentally Ill Persons/

3. Mental Health/

4. ((mental* or psych*) adj (health or status* or ill* or condition* or disorder* or disease* or problem*)).ti,ab.

5. "Agoraphobia"/ or "anankastic personality disorder"/ or "Anorexia Nervosa"/ or "Antisocial Personality Disorder"/ or "Attention Deficit and Disruptive Behavior Disorders"/ or "Attention Deficit Disorder with Hyperactivity"/ or "avoidant personality disorder"/ or "Body Dysmorphic Disorders"/ or "Borderline Personality Disorder"/ or "Bulimia Nervosa"/ or "Bulimia"/ or "Catatonia"/ or "Compulsive Behavior"/ or "Compulsive Personality Disorder"/ or "Conduct Disorder"/ or "Conversion Disorder"/ or "Cyclothymic Disorder"/ or "Delirium, Dementia, Amnestic, Cognitive Disorders"/ or "Dependency (Psychology)"/ or "Dependent Personality Disorder"/ or "Depersonalization"/ or "Depressive Disorder, Major"/ or "Dissociative Disorders"/ or "Dysthymic Disorder"/ or "Eating Disorders"/ or "Feeding Behavior"/ or "Hallucinations"/ or "histrionic personality disorder"/ or "Hysteria"/ or "Mental Disorders"/ or "Mood Disorders"/ or "Multiple Personality Disorder"/ or "narcissistic personality disorder"/ or "Neurasthenia"/ or "Neurotic Disorders"/ or "Obsessive Behavior"/ or "obsessive compulsive personality disorder"/ or "Obsessive-Compulsive Disorder"/ or "Panic Disorder"/ or "Panic"/ or "Paranoid Personality Disorder"/ or "passive-aggressive personality disorder"/ or "Personality Disorders"/ or "Phobic Disorders"/ or "Psychophysiologic Disorders"/ or "Rett Syndrome"/ or "Shared Paranoid Disorder"/ or "Social Behavior Disorders"/ or "Somatoform Disorders"/ or "Stress Disorders, Post- Traumatic"/ or "Delusions"/

6. "Adjustment Disorders"/ or exp Amnesia/ or exp "Attention Deficit and Disruptive Behavior Disorders"/ or "Binge-Eating Disorder"/ or exp "Cognition Disorders"/ or exp "Communication Disorders"/ or "Consciousness Disorders"/ or "Coprophagia"/ or "Delirium"/ or "Developmental Disabilities"/ or exp "Dyslexia, Acquired"/ or "Factitious Disorders"/ or "Impulse Control Disorders"/ or "Motor Skills Disorders"/ or "Munchausen Syndrome"/ or "Neurocirculatory Asthenia"/ or exp "Obsessive-Compulsive Disorder"/ or "Pica"/ or "Stereotypic Movement Disorder"/ or exp "Stress Disorders, Traumatic"/ or exp "Child Development Disorders, Pervasive"/ or "Mental Disorders Diagnosed in Childhood"/ or "Depression, Postpartum"/ or "Depressive Disorder, Treatment-Resistant"/ or "Seasonal Affective Disorder"/

7. ("anankastic personalit*" or "anorexia nervosa" or "antisocial personalit*" or "attention deficit disorder*" or "body dysmorphic" or "conduct disorder*" or "cyclothymic personalit*" or "endogenous depression" or "folie a deux" or "obsessive compulsive" or "panic disorder" or "panic disorders" or "pervasive developmental" or "post traumatic" or "seasonal affective" or "affective disorder*" or "avoidant personalit*" or "behavio?r disorder*" or "behavio?r problem*" or "behavioral disorder*" or "behavioural disorder*" or "conversion disorder*" or "eating behavio?r" or "eating adj1 disorder*" or "overactive disorder*" or (personality adj3 disorder*) or agoraphobia or (anankastic adj1 person*) or (antisocial adj1 person*) or (asocial adj1 person*) or Asperger* or autism or autistic or (avoidant adj1 person*) or "borderline personalit*" or bulimia or catatonia or catatonic or compulsion* or (compulsive adj1 person*) or (conversion adj1 disorder*) or cyclothymia or (dependent adj1 personalit*) or depersonali?ation or dereali?ation or disintegrative or (dissocial adj1 person*) or dissociation* or dissociative or dysthym* or fugue or hallucination* or hebephreni* or (histrionic adj1 person*) or hyperkinetic or hypomania or hysteria or mania* or manic* or (narcissistic adj1 person*) or neurasthenia or neurosis or neurot* or (obsessive adj1 person*) or oligophreni* or paranoia or paranoid or (passive-aggressive adj1 person*) or phobia* or phobic or posttraumatic or PTSD or psychopath* or rett or rett?s or retts or sociopath* or somati?ation or somatoform).ti,ab.

8. (amnesi* or hypomania or cyclothymia or dysthymia or delirium or hallucinosis or delusional or (mood adj2 disorder*) or asthenic or "emotionally labile" or postencephalitic or postconcussion* or (trance adj1 disorder*) or (possession adj1 disorder*) or obsessional or "severe stress" or (adjustment adj1 disorder*) or dissociate or "multiple personality" or neurasthenia or (psychological adj1 disturbance*) or (psychologically adj1 disturbed) or suicid* or parasuicid* or (self adj1 harm*) or (self adj1 injur*) or comorbid* or "co morbid*" or multimorbid* or "multi morbid*" or bulimi* or anorexi* or neuros* or OCD or "psychological stress" or "psychological distress" or "mental stress" or "adjustment disorder" or "adjustment disorders" or "Psychological Sexual Dysfunction*" or "Psychosexual Dysfunction*" or "Psychosexual Disorder*" or "Sexual Aversion Disorder*" or "Orgasmic Disorder*" or "Sexual Arousal Disorder*" or "Hypoactive Sexual Desire Disorder*" or "Pathological Gambling" or Trichotillomania or "Sleep Disorder*" or "Substance Related Disorder*" or "Impulse Control Disorder*" or "Explosive Disorder*" or Kleptomania or "Firesetting Behavio?r*" or Pyromania* or "Substance Use Disorder*").ti,ab.

9. or/1-8

10. "emigrants and immigrants"/ or ethnic groups/ or arabs/ or gypsies/ or jews/ or refugees/ or "transients and migrants"/ or Cross-cultural comparison/ or exp "human migration"/ or "ethnology"/

11. exp continental population groups/ or exp african continental ancestry group/ or exp asian continental ancestry group/ or exp european continental ancestry group/ or exp oceanic ancestry group/

12. (diaspora* or multicultural or "multi cultural" or (crosscultural or "cross cultural") or (transcultural or "trans cultural") or (multiethnic or "multi ethnic") or (multiracial or "multi racial") or biracial or migrant* or immigrant* or refugee* or "cultural diversity" or "culturally diverse" or "racial diversity" or "racially diverse" or "ethnic diversity" or "ethnically diverse" or superdivers* or "super divers*" or "super-divers*" or (multilingual or "multi lingual") or (traveller* or Gypsies or Gypsy or Gipsy or Gipsies or Romany or Romanies or Romani or Romanis or Rromani or Rromanis or Roma) or "asylum seeker*" or "seeking asylum" or "mixed race*" or ethnocultural or sociocultural or ethnoracial or "ethno cultural" or "socio cultural" or "ethno racial" or "diverse population*" or "ethnic difference*" or ethnicity or indigenous or "minority ethnic" or "ethnic data" or "race data" or "ethnic classification" or "race classification" or "ethnic group*" or "racial group*" or "ancestry group*" or "cultur* difference*" or ethnic* or heritage).ti,ab.

13. (black* or "Black African*" or (Black adj1 British) or "British African*" or "Afrocaribbean*" or "Afro caribbean*" or (Black adj1 caribbean*) or Caucasian* or "South American*" or "Central American*" or Balkan* or "Mixed white" or "Mixed black" or Jews or Jewish or "Non white*" or nonwhite* or BME or "Black and Minority Ethnic" or "Eastern Europe*" or Hispanic* or Latin*).ti,ab.

14. ((minorit* adj3 (culture* or m?n or wom?n or male* or female* or adult* or adolescent* or teenager* or "teen ager*" or child* or young* or old* or elder* or people* or person* or individual* or member* or population* or communit* or neighbourhood* or neighborhood* or group* or area* or demograph*)) or (ethnic adj3 (culture* or people* or population* or communit* or neighbourhood* or neighborhood* or group* or area* or demograph* or minorities or minority)) or "ethnic origin*" or (White adj2 (minorities or minority))).ti,ab.

15. (((black or white) adj3 (culture* or ethnic* or m?n or wom?n or male* or female* or adult* or adolescent* or teenager* or "teen ager*" or child* or young* or old* or elder* or people* or person* or individual* or population* or communit* or neighbourhood* or neighborhood* or group* or area* or demograph* or minorities or minority)) or ((displaced or alien) adj2 (people* or person* or individual*)) or (born adj2 overseas) or ((vulnerable or disadvantaged or marginal* or transient or undocumented) adj1 (people* or person* or individual* or population* or communit* or neighbourhood* or neighborhood* or group* or area* or demograph*))).ti,ab.

16. ((arab*or somali* or yemini* or Vietnamese or Chinese or Caribbean* or Pakistani* or Bangladeshi* or Punjabi* or Somali* or Gujarati* or Japanese or Asian* or Irish or Indian* or Bengali* or Afghanistani* or Turkish or Kurdish or Yemeni* or Albanian* or Polish or German* or African* or American* or Jamaican* or Nigerian* or Kenyan* or Zimbabwean* or Philippin* or Filipin* or "Sri Lankan*" or French or Italian or Chinese or Cantonese or Australia* or Somalia* or Portugues* or Canadian* or Ghanaian* or Lithuanian* or "Hong Kong" or Spanish or Iranian* or "New Zealand" or Romania* or Iraqi* or Turkish or Cypriot* or Malaysian* or Dutch or Ugandan* or Bulgarian* or Afghan* or Brazilian* or Slovak* or Mauritan* or Singapore* or Nepales* or Hungarian* or Latvian* or Russian* or Tanzanian* or Thai* or Swedish or Greek or Zambia* or Czech or Egyptia* or Trinidad* or Tobago* or Maltese or Austrian* or Belgian* or Libyan* or Korean* or Danish or Swiss) adj3 (culture* or m?n or wom?n or male* or female* or adult* or adolescent* or teenager* or "teen ager*" or child* or young* or old* or elder* or people* or person* or individual* or population* or communit* or neighbourhood* or neighborhood* or group* or area* or demograph* or minorities or minority or ethnic*)).ti,ab.

17. or/10-16

18. exp United Kingdom/

19. ("United Kingdom" or UK or "U.K." or "Great Britain" or Britain or GB or "G.B.").ti,ab.

20. (England or Scotland or Wales or "Northern Ireland").ti,ab.

21. (British or English or Scottish or Welsh or "Northern Irish").ti,ab.

22. or/18-21

23. 9 and 17 and 22

Search for inequality reviews related to any of the outcomes (combined with general terms above)

| 1. exp Socioeconomic Factors/ |  |
| --- | --- |
| 2. ((social adj2 depriv*) or (social adj2 disadvantage*) or equity or inequity or (health adj2 difference*) or (health adj2 disparit*) or (health adj2 equit*) or inequalit* or inequit* or "health service access*" or (health adj2 variation*) or marginalized or marginalised or ((multipl* or double or severe or serious) adj2 (depriv* or disadvantage*)) or poverty or (social adj class) or (social adj2 condition*) or (social adj2 depriv*) or (social adj2 difference*) or (social adj2 disparit*) or (social adj environment) or (social* adj2 exclu*) or (social adj2 factor*) or (social adj2 gradient*) or (social adj inclusion) or (social adj inequalit*) or (social adj inequit*) or (social* adj isolat*) or (social adj justice) or (social adj position) or (social adj security) or (social adj2 variation*) or (social adj welfare) or (social* adj 2 exclu*) or (socio adj economic adj attribut*) or (socio adj economic adj circumstanc*) or (socio adj economic adj factor*) or (socio adj economic adj gradient*) or (socio adj economic adj health* adj difference*) or (socio adj economic adj position) or (socio adj economic adj status) or (socio adj economic adj variable*) or (socioeconomic adj2 attribut*) or (socioeconomic adj circumstance*) or (socioeconomic adj factor*) or (socioeconomic adj gradient*) or (socioeconomic adj health adj difference*) or (socioeconomic adj position) or (socioeconomic adj status) or (socioeconomic adj variable*) or (state adj benefit*) or (uncompensated adj care) or underprivilege* or unemployed or unemployment or (vulnerable adj2 population*) or (vulnerable adj2 group*) or (vulnerable adj2 communit*) or (vulnerable adj2 people) or (vulnerable adj2 person*) or welfare or workless*).ti,ab. |  |
| 3. or/1-2 |  |

4. meta analysis.mp,pt. or review.pt. or search:.tw.

5. 3 and 4

6. limit 5 to (english language and humans)

7. remove duplicates from 6

Specific search for prevalence and incidence reviews (combined with the general search terms)

1. Prevalence/ or Incidence/

2. exp Population Surveillance/

3. "Family Characteristics"/ or "population dynamics"/ or exp "sex distribution"/ or "health transition"/ or exp "human migration"/ or "population characteristics"/ or "censuses"/ or "age distribution"/ or "ethnology"/ or "health status disparities"/

4. Demography/

5. Cross-cultural comparison/

6. Health Surveys/

7. Longitudinal Studies/

8. Epidemiology/

9. (prevalen* or incidence*).ti,ab.

10. survey*.ti,ab.

11. ("population stud*" or "community stud*" or "household*" or "community sample*" or "population sample*" or "community level" or "population level" or "community comparison*" or "population comparison*").ti,ab.

12. epidemiolog*.ti,ab.

13. or/1-12

14. meta analysis.mp,pt. or review.pt. or search:.tw.

15. 13 and 14

16. limit 15 to (english language and humans)

17. remove duplicates from 16

Specific search for interventions reviews (combined with the general search terms)

1. exp Therapeutics/ or exp Psychotherapy/

2. (treatment* or intervention* or therap* or psychotherap* or program*).ti,ab.

| 3. randomi?ed controlled trial.pt. |  |
| --- | --- |
| 4. controlled clinical trial.pt. |  |
| 5. randomi?ed.ab. |  |
| 6. placebo.ab. |  |
| 7. clinical trials as topic.sh. |  |
| 8. randomly.ab. |  |

9. trial.ti.

10. or/1-9

11. meta analysis.mp,pt. or review.pt. or search:.tw.

12. 10 and 11

13. limit 12 to (english language and humans)

14. remove duplicates from 13

Specific search for pathways reviews (combined with the general search terms)

1. exp "Referral and Consultation"/

2. exp "Critical Pathways"/

3. (pathway* adj2 care).mp.

4. (pathway* adj2 "health care").mp.

5. (pathway* adj2 "mental health care").mp.

6. (pathway* adj2 "psychiatric care").mp.

7. ((access* adj2 (service* or care)) or (service* adj2 (use* or usage* or utili?ation*)) or detention* or admission*).ti,ab.

8. (pathway* adj2 service*).mp.

9. (pathway* adj2 "health service*").mp.

10. (pathway* adj2 "mental health service*").mp.

11. (pathway* adj2 "psychiatric service*").mp.

12. exp "Health Services Accessibility"/ or exp "Commitment of Mentally Ill"/

13. ("general practitioner*" or GP or police or forensic or criminal or "Mental Health Act" or MHA or "duration of untreated psychosis" or DUP or "duration of untreated illness" or DUI).ti,ab.

14. or/1-13

15. meta analysis.mp,pt. or review.pt. or search:.tw.

16. 14 and 15

17. limit 16 to (english language and humans)

18. remove duplicates from 17

Specific search for pathways primary literature 2012-2017 (combined with general search terms)

1. exp "Referral and Consultation"/

2. exp "Critical Pathways"/

3. (pathway* adj2 care).mp.

4. (pathway* adj2 "health care").mp.

5. (pathway* adj2 "mental health care").mp.

6. (pathway* adj2 "psychiatric care").mp.

7. ((access* adj2 (service* or care)) or (service* adj2 (use* or usage* or utili?ation*)) or detention* or admission*).ti,ab.

8. (pathway* adj2 service*).mp.

9. (pathway* adj2 "health service*").mp.

10. (pathway* adj2 "mental health service*").mp.

11. (pathway* adj2 "psychiatric service*").mp.

12. exp "Health Services Accessibility"/ or exp "Commitment of Mentally Ill"/

13. ("general practitioner*" or GP or police or forensic or criminal or "Mental Health Act" or MHA or "duration of untreated psychosis" or DUP or "duration of untreated illness" or DUI).ti,ab.

14. or/1-13

15. limit 14 to (english language and humans)

16. remove duplicates from 15
