# Supplementary material for: Ethnic inequalities and pathways to care in psychosis in England: a systematic review and meta-analysis
Source: BMC Med. 2018 Dec 12;16:223. doi: 10.1186/s12916-018-1201-9 (PMC6290527; doi:10.1186/s12916-018-1201-9)
Supplement: Supplementary file 2 — AMSTAR quality assessment (for review of reviews). (DOCX 39 kb) [file 12916_2018_1201_MOESM2_ESM.docx]

**Additional file 2:**

**AMSTAR quality assessment (for review of reviews)**

AMSTAR questions used in the table below:

1. Was an 'a priori' design provided?
2. Was there duplicate study selection and data extraction?
3. Was a comprehensive literature search performed?
4. Was the status of publication (i.e. grey literature) used as an inclusion criterion?
5. Was a list of studies (included and excluded) provided?
6. Were the characteristics of the included studies provided?
7. Was the scientific quality of the included studies assessed and documented?
8. Was the scientific quality of the included studies used appropriately in formulating conclusions?
9. Were the methods used to combine the findings of studies appropriate?
10. Was the likelihood of publication bias assessed?
11. Was the conflict of interest included?

Pathways to care systematic reviews have been highlighted in grey in the table below.

| **Reference** | **1** | **2** | **3** | **4** | **5** | **6** | **7** | **8** | **9** | **10** | **11** | **AMSTAR score** | **Quality: low (0-4 points), medium (5-8 points),**  **high (9-11 points)** |
| --- | --- | --- | --- | --- | --- | --- | --- | --- | --- | --- | --- | --- | --- |
| Adelman et al 2009 | No | Can't answer | Yes | No | No | Yes | Yes | Yes | Can't answer | No | No | 4 | Low |
| Aggarwal et al 2016 | Yes | Yes | Yes | Yes | No | Yes | Yes | Yes | Yes | No | No | 8 | Medium |
| Al-Sharifi et al 2015 | No | Can't answer | No | No | No | Yes | Yes | No | Yes | No | No | 3 | Low |
| Anderson et al 2010 | No | Can't answer | Yes | No | No | Yes | No | No | Yes | No | No | 3 | Low |
| Anderson et al 2014a | No | Can't answer | Yes | No | Yes | Yes | Yes | Yes | Yes | Yes | No | 7 | Medium |
| Anderson et al 2014b | No | No | Yes | No | Yes | Yes | Yes | Yes | Yes | No | No | 6 | Medium |
| Anderson et al 2017 | Yes | No | Yes | No | No | Yes | Yes | Yes | Yes | No | No | 6 | Medium |
| Arblaster et al 1996 | No | Can't answer | Yes | Yes | No | No | No | No | Yes | No | No | 3 | Low |
| Atkinson et al 2001 | Yes | Yes | Yes | Yes | No | No | No | No | Yes | No | No | 5 | Medium |
| Badger et al 1999 | No | Can't answer | Yes | Yes | Yes | Yes | Yes | Yes | Can't answer | No | No | 6 | Medium |
| Bee et al 2008 | No | Yes | Yes | Yes | Yes | Can't answer | No | No | Yes | No | No | 5 | Medium |
| Bhattacharyya and Benbow 2013 | No | Yes | No | No | No | Yes | No | No | Can't answer | No | No | 2 | Low |
| Bhui et al 2003 | Yes | Yes | Yes | Yes | No | Yes | Yes | Yes | Yes | Yes | No | 9 | High |
| Bhui et al 2007 | No | Yes | No | No | No | Yes | Yes | Yes | Yes | No | No | 5 | Medium |
| Bhui et al 2015 | Yes | Yes | Yes | Yes | No | Yes | Yes | Yes | Yes | No | No | 8 | Medium |
| Bogic et al 2015 | Yes | Yes | Yes | Yes | No | Yes | Yes | Yes | Yes | Yes | No | 9 | High |
| Bourque et al 2011 | No | Yes | Yes | No | No | Yes | Yes | Yes | Yes | Yes | No | 7 | Medium |
| Bronstein and Montgomery 2011 | Yes | Can't answer | Yes | Yes | No | Yes | No | No | Yes | No | No | 5 | Medium |
| Cantor-Graae and Selten 2005 | No | Can't answer | Yes | No | No | Yes | No | No | Yes | Yes | No | 4 | Low |
| Carr et al 2014 | Yes | Yes | Yes | Yes | No | No | No | No | Yes | No | No | 5 | Medium |
| Carra and Johnson 2009 | No | Can't answer | Yes | No | No | Yes | No | No | Yes | No | No | 3 | Low |
| Chorlton et al 2011 | No | Can't answer | Yes | Yes | No | Yes | Yes | Yes | Yes | No | No | 6 | Medium |
| Churchill et al 1999 | No | Can't answer | Yes | Yes | Yes | Yes | No | No | Can't answer | No | No | 4 | Low |
| Clement et al 2014 | Yes | Yes | Yes | No | Yes | Yes | No | No | Yes | No | No | 6 | Medium |
| Cooper 2005 | No | No | Yes | Yes | No | No | No | No | Can't answer | No | No | 2 | Low |
| Cooper et al 2010 | No | Can't answer | Yes | No | No | Yes | Yes | No | Can't answer | No | No | 3 | Low |
| Denzel et al 2016 | No | Can't answer | Yes | No | No | Yes | Yes | Yes | Yes | No | No | 5 | Medium |
| Dixon-Woods et al 2005 | No | No | Yes | Yes | No | Yes | No | No | Yes | No | No | 4 | Low |
| Durà-Vilà and Hodes 2012 | No | Yes | Yes | No | No | Yes | Yes | Yes | Yes | No | No | 6 | Medium |
| Elvish et al 2012 | No | Can't answer | No | No | No | Yes | Yes | No | Can't answer | No | No | 2 | Low |
| Fazel et al 2005 | Can't answer | Can't answer | Yes | Yes | No | No | No | No | Yes | No | No | 3 | Low |
| Filges et al 2015 | Yes | Yes | Yes | Yes | Yes | Yes | Yes | Yes | Yes | Yes | No | 10 | High |
| Fusar-Poli et al 2017 | No | Yes | Yes | No | No | No | Yes | No | Yes | Yes | No | 5 | Medium |
| Garrett et al 2012 | No | Can't answer | Yes | No | No | Yes | No | No | Yes | No | No | 3 | Low |
| Goodman et al 2008 | Yes | Yes | Yes | Yes | Yes | Yes | Yes | Yes | Yes | No | No | 9 | High |
| Kalt et al 2013 | No | Can't answer | No | No | No | Yes | No | No | Yes | No | No | 2 | Low |
| Kapadia et al 2017 | No | Can't answer | Yes | Yes | No | Yes | No | No | Yes | No | No | 4 | Low |
| Kirkbride et al 2012 | Yes | Yes | Yes | Yes | No | No | Yes | Yes | Yes | Yes | No | 8 | Medium |
| Lamb et al 2012 | No | Can't answer | No | No | No | Yes | No | No | Yes | No | No | 2 | Low |
| Mann et al 2014 | No | Can't answer | Yes | Yes | No | Yes | Yes | Yes | Yes | No | No | 6 | Medium |
| McGilloway et al 2010 | No | Can't answer | Yes | No | No | Yes | No | No | Yes | No | No | 3 | Low |
| McGrath et al 2004 | No | Can't answer | Yes | Yes | No | Yes | Yes | Yes | Yes | No | No | 6 | Medium |
| Mindlis and Boffetta 2017 | Yes | Can't answer | Yes | No | No | No | Yes | Yes | Yes | Yes | No | 6 | Medium |
| Moffat et al 2009 | No | Yes | Yes | Yes | No | Yes | Yes | Yes | Can't answer | Not applicable | No | 6 | Medium |
| Montesinos et al 2013 | No | Can't answer | Yes | No | No | Yes | No | No | Can't answer | No | No | 2 | Low |
| Mukadam et al 2011 | No | No | Yes | No | No | Yes | Yes | Yes | Yes | No | No | 5 | Medium |
| Newbigging et al 2013 | No | Yes | Yes | Yes | No | No | No | No | Can't answer | No | No | 3 | Low |
| O’Donoghue et al 2016 | No | Can't answer | Yes | Yes | No | Yes | Yes | No | No | No | No | 4 | Low |
| Paradies et al 2013 | No | Yes | Yes | Yes | No | Yes | No | No | Can't answer | No | No | 6 | Medium |
| Pearson et al 2007 | No | Can't answer | Yes | Yes | Yes | Yes | No | No | Yes | No | No | 5 | Medium |
| Rees et al 2016 | Yes | No | Yes | Yes | No | No | No | No | Yes | No | No | 4 | Low |
| Robjant et al 2009 | No | Can't answer | Yes | No | No | Yes | No | No | Can't answer | No | No | 2 | Low |
| Sass et al 2009 | No | Yes | Yes | No | No | Yes | Yes | Yes | Yes | No | No | 6 | Medium |
| Sigvardsdotter et al 2016 | No | Can't answer | Yes | No | No | Yes | Yes | No | Yes | No | No | 4 | Low |
| Singh and Grange 2006 | No | Can't answer | Yes | No | No | Yes | No | No | Yes | No | No | 3 | Low |
| Singh et al 2007 | No | Yes | Yes | Yes | No | Yes | No | No | Yes | No | No | 5 | Medium |
| Singh et al 2013 | No | Can't answer | Yes | Yes | No | Yes | Yes | No | Yes | No | No | 5 | Medium |
| Spallek et al 2014 | No | Can't answer | No | No | No | Yes | No | No | Yes | No | No | 2 | Low |
| Storm and Engberg 2013 | No | Yes | Yes | No | No | Yes | No | No | Can't answer | No | No | 3 | Low |
| Swinnen and Selten 2007 | No | Can't answer | Yes | No | No | Yes | No | No | Yes | Yes | No | 3 | Low |
| Tortelli et al 2015 | No | Can't answer | Yes | Yes | No | Yes | No | No | Yes | Yes | No | 5 | Medium |
| Uppal and Bonas 2014 | No | Can't answer | Yes | No | No | Yes | No | No | Can't answer | No | No | 2 | Low |
| Voracek and Loibl 2008 | No | Can't answer | Yes | Yes | No | Yes | No | No | Yes | Yes | No | 5 | Medium |
| Werner et al 2014 | No | Can't answer | Yes | No | No | Yes | No | No | Can't answer | No | No | 2 | Low |
| Yang et al 2014 | Yes | Yes | Yes | No | No | Yes | No | No | Can't answer | No | No | 5 | Medium |
