# Supplementary material for: Ethnic inequalities and pathways to care in psychosis in England: a systematic review and meta-analysis
Source: BMC Med. 2018 Dec 12;16:223. doi: 10.1186/s12916-018-1201-9 (PMC6290527; doi:10.1186/s12916-018-1201-9)
Supplement: Supplementary file 3 — Reasons for exclusion on full text. (DOCX 93 kb) [file 12916_2018_1201_MOESM3_ESM.docx]

**Additional file 3:**

**Reasons for exclusion on full text**

Review of reviews

Not a systematic review (n=181)

Not ethnicity (n=66)

Not UK (n=25)

Not published (protocol) (n=17)

Not UK or ethnicity (n=8)

Separate UK and ethnicity data, but no UK data by ethnicity (n=6)

Wrong publication type (n=4)

Not mental health (n=3)

| **Reference** | **Main reason for exclusion** |
| --- | --- |
| Adi, Y., Killoran, A., Schrader McMillan, A., & Stewart-Brown, S. (2007). Systematic review of interventions to promote mental wellbeing in primary schools. Coventry: The University of Warwick. | Not ethnicity |
| Ahmad, W. I., Kernohan, E. E., & Baker, M. R. (1989). Health of British Asians; a research review. *Community Medicine, 11*(1), 49-56. | Not a systematic review |
| Amick, H. R., Gartlehner, G., Gaynes, B. N., Forneris, C., Asher, G. N., Morgan, L. C., Coker-Schwimmer, E., Boland, E., Lux, L. J. Gaylord, S., Bann, C., Pierl, C. B., & Lohr, K. N. (2015). Comparative benefits and harms of second generation antidepressants and cognitive behavioral therapies in initial treatment of major depressive disorder: systematic review and meta-analysis. *British Medical Journal, 351*. doi:10.1136/bmj.h6019 | Separate UK and ethnicity data, but no UK data by ethnicity |
| Anand, A. S., & Cochrane, R. (2005). The Mental Health Status of South Asian Women in Britain: A Review of the UK Literature. *Psychology and Developing Societies, 17*(2), 195-214. doi:10.1177/097133360501700207 | Not a systematic review |
| Arai, L., & Harding, S. (2004). A review of the epidemiological literature on the health of UK-born Black Caribbeans. *Critical Public Health, 14*(2), 81-116. | Not a systematic review |
| Attree, P., French, B., Milton, B., Povall, S., Whitehead, M., & Popay, J. (2011). The experience of community engagement for individuals: a rapid review of evidence. *Health & Social Care in the Community, 19*(3), 250-260. | Not a systematic review |
| Bados, A., Balaguer, G., & Saldaña, C. (2007). Outcome of cognitive-behavioural therapy in training practice with anxiety disorder patients. *British Journal of Clinical Psychology*, *46*(4), 429-435. | Not ethnicity |
| Barnes, M., & Coelho, V. S. (2009). Social participation in health in Brazil and England: Inclusion, representation and authority. *Health Expectations, 12*(3), 226-236. | Not ethnicity |
| Barnoux, M., & Wood, J. (2013). The specific needs of foreign national prisoners and the threat to their mental health from being imprisoned in a foreign country. *Aggression and Violent Behavior, 18*(2), 240-246. | Not a systematic review |
| Baumgartner, J. N., & Burns, J. K. (2014). Measuring social inclusion--a key outcome in global mental health. *International Journal of Epidemiology, 43*(2), 354-364. | Not ethnicity |
| Bee, P., Price, O., & Baker, J. (2015). Systematic synthesis of barriers and facilitators to service user-led care planning. T*he British Journal of Psychiatry, 207*(2), 104-114. | Separate UK and ethnicity data, but no UK data by ethnicity |
| Bennett, J., Kalathil, J., & Keating, F. (2007). One size doesn't fit all: new evidence suggests a need for a radical review of current standards of race equality training for mental health staff. *Mental Health Today*, 28-31. | Not a systematic review |
| Bennett, J., & Keating, F. (2009). Training to redress racial disadvantage in mental health care: race equality or cultural competence? *Journal of Public Mental Health, 8*(2), 40-46. | Not a systematic review |
| Berry, J. G., Bloom, S., Foley, S., & Palfrey, J. S. (2010). Health inequity in children and youth with chronic health conditions. *Pediatrics, 126 Suppl 3*, S111-119. | Not a systematic review |
| Bhopal, R., Vettini, A., Hunt, S., Wiebe, S., Hanna, L., & Amos, A. (2004). Review of prevalence data in, and evaluation of methods for cross cultural adaptation of, UK surveys on tobacco and alcohol in ethnic minority groups. *BMJ: British Medical Journal (International Edition), 328*(7431), 76-80. | Not a systematic review |
| Bhugra, D., Mallett, R., & Leff, J. (1999). Schizophrenia and African-Caribbeans: A conceptual model of aetiology. *International Review of Psychiatry, 11*(2), 145-152. | Not a systematic review |
| Bhui, K. (1999). Common mental disorders among people with origins in or immigrant from India and Pakistan. *International Review of Psychiatry, 11*(2-3), 136-144. doi:10.1080/09540269974302 | Not a systematic review |
| Bhui, K., Christie, Y., & Bhugra, D. (1995). The essential elements of culturally sensitive psychiatric services. *International Journal of Social Psychiatry, 41*(4), 242-256. | Not a systematic review |
| Bhui, K., Dinos, S., & McKenzie, K. (2012). Ethnicity and its influence on suicide rates and risk. *Ethn Health, 17*(1-2), 141-148. | Not a systematic review |
| Bhui, K., Warfa, N., Edonya, P., McKenzie, K., & Bhugra, D. (2007). Cultural competence in mental health care: a review of model evaluations. *BMC Health Services Research, 7*(15). doi:10.1186/1472-6963-7-15 | Not UK |
| Blackmore, R., Boyle, J., Gray, K., Willey, S., Ranasinha, S., Misso, M., Fazel, M., & Gibson-Helm, M. (2016). Prevalence of mental illness in refugees: a systematic review. *PROSPERO*. <http://www.crd.york.ac.uk/PROSPERO/display_record.asp?ID=CRD42016046349> | Not published (protocol) |
| Blythe, J., & White, J. (2012). Role of the mental health nurse towards physical health care in serious mental illness: An integrative review of 10 years of UK literature. *International Journal of Mental Health Nursing, 21*(3), 193-201. | Not ethnicity |
| Borschmann, R. D., Gillard, S., Turner, K., Chambers, M., & O'Brien, A. (2010). Section 136 of the Mental Health Act: a new literature review. *Medicine, Science & the Law, 50*(1), 34-39. | Not a systematic review |
| Botsford, J., Clarke, C. L., & Gibb, C. E. (2011). Research and dementia, caring and ethnicity: A review of the literature. *Journal of Research in Nursing, 16*(5), 437-449. | Not a systematic review |
| Bowers, L., Banda, T., & Nijman, H. (2010). Suicide inside: A systematic review of inpatient suicides. *Journal of Nervous and Mental Disease, 198*(5), 315-328. | Not ethnicity |
| Bowers, L., Jeffery, D., Bilgin, H., Jarrett, M., Simpson, A., & Jones, J. (2008). Psychiatric intensive care units: a literature review. *International Journal of Social Psychiatry, 54*(1), 56-68. | Not a systematic review |
| Bowes, A. (Principal Investigator). (2009-2011). Dignity and respect in residential care: issues for Black and Minority Ethnic groups. University of Stirling. | Not mental health |
| Bradbury-Jones, C., Rattray, J., Jones, M., & Macgillivray, S. (2013). Promoting the health, safety and welfare of adults with learning disabilities in acute care settings: A structured literature review. *Journal of Clinical Nursing, 22*(11), 1497-1509. | Not ethnicity |
| Bradshaw, T., Lovell, K., & Harris, N. (2005). Healthy living interventions and schizophrenia: A systematic review. *Journal of Advanced Nursing, 49*(6), 634-654. | Not ethnicity |
| Braganza, J. (2001). Attempted suicide by Bangladeshi adolescent girls. *Paediatric Nursing, 13*(2), 26-29. | Not a systematic review |
| Broad, B., & Robbins, I. (2005). The wellbeing of unaccompanied asylum seekers leaving care. *Diversity in Health & Social Care, 2*(4), 271-277. | Not a systematic review |
| Brophy, C., & Morris, D. (2014). Community-oriented integrated mental health services. *London J Prim Care (Abingdon), 6*(6), 159-163. | Not ethnicity |
| Brown, T. J., Todd, A., O’Malley, C. L., Moore, H. J., Husband, A. K., Bambra, C., Kasim, A., Sniehotta, F. F., Steed, L., & Summerbell, C. D. (2016). Community pharmacy interventions for public health priorities: a systematic review of community pharmacy-delivered smoking, alcohol and weight management interventions. *Public Health Research, 4*(2). doi:10.3310/phr04020 | Not ethnicity |
| Brownley, K. A., Berkman, N. D., Peat, C. M., Lohr, K. N., Cullen, K. E., Bann, C. M., & Bulik, C. M. (2016). Binge-Eating Disorder in Adults: A Systematic Review and Meta-analysis. *Annals of Internal Medicine, 165*(6), 409-420. | Not UK or ethnicity |
| Bunn, M., Goesel, C., Kinet, M., & Ray, F. (2016). Group treatment for survivors of torture and severe violence: a literature review. *Torture, 26*(1), 45-67. | Not a systematic review |
| Burman, E., & Chantler, K. (2005). Domestic violence and minoritisation: Legal and policy barriers facing minoritized women leaving violent relationships. *International Journal of Law and Psychiatry, 28*(1), 59-74. | Not a systematic review |
| Burns, T., Knapp, M., Catty, J., Healey, A., Henderson, J., Watt, H., & Wright, C. (2001). Home treatment for mental health problems: a systematic review. *Health Technol Assess, 5*(15). <https://www.journalslibrary.nihr.ac.uk/hta/hta5150#/abstract> | Not ethnicity |
| Butler, M., Warfa, N., Khatib, Y. & Bhui, K. (2015). Migration and common mental disorder: an improvement in mental health over time?. *International Review of Psychiatry, 27*(1), 51-63. | Not a systematic review |
| Cabassa, L. J., Ezell, J. M., , L.-F., & ez, R. (2010). Lifestyle interventions for adults with serious mental illness: a systematic literature review. *Psychiatric Services, 61*(8), 774-782. | Not UK |
| Campbell, C., & Burgess, R. (2012). The role of communities in advancing the goals of the Movement for Global Mental Health. *Transcultural Psychiatry, 49*(3), 379-395. | Not UK or ethnicity |
| Campbell, C., Cornish, F., & McLean, C. (2004). Social capital, participation and the perpetuation of health inequalities: Obstacles to African-Caribbean participation in 'partnerships' to improve mental health. *Ethnicity and Health, 9*(4), 313-335. | Not a systematic review |
| Cantor-Graae, E. (2008). Review: black and minority ethnic people are more likely to be detained under the Mental Health Act 1983--no clear evidence why. *Evidence Based Mental Health, 11*(2), 61-61. | Not a systematic review |
| Castillejos-Anguiano, M.C., Moreno-Küstner, B., Gómez-García, B., & Martin, C. (2016). Variability in the incidence of psychosis worldwide: enviromental factors and methodological issues. A systematic review and meta-analyses. *PROSPERO*.  <http://www.crd.york.ac.uk/PROSPERO/display_record.asp?ID=CRD42016050902> | Not published (protocol) |
| Carr, S., Lhussier, M., Forster, N., Geddes, L., & Deane, K. (2011) An evidence synthesis of qualitative and quantitative research on component intervention techniques, effectiveness, cost-effectiveness, equity and acceptability of different versions of health-related lifestyle advisor role in improving health. *Health Technol Assess*, *15*(9). doi:10.3310/hta15090 | Not ethnicity |
| Centre for Reviews & Dissemination (1996). Ethnicity and health: reviews of literature and guidance for purchasers in the areas of cardiovascular disease, mental health and haemoglobinopathies. The University of York. <https://www.york.ac.uk/media/crd/crdreport5.pdf> | Not a systematic review |
| Chadwick, A., Street, C., McAndrew, S., & Deacon, M. (2012). Minding our own bodies: Reviewing the literature regarding the perceptions of service users diagnosed with serious mental illness on barriers to accessing physical health care. *International Journal of Mental Health Nursing, 21*(3), 211-219. | Not ethnicity |
| Chakraborty, A., & McKenzie, K. (2002). Does racial discrimination cause mental illness? *The British Journal of Psychiatry, 180*(6), 475-477. | Not a systematic review |
| Chambers, M., Connor, S. L., & Davren, M. (2006). An evaluation of a combined education and multi-project practice development programme in mental health. *Journal of Psychiatric and Mental Health Nursing, 13*(3), 364-371. | Not a systematic review |
| Chew-Graham, C., Burroughs, H., & Hibbert, D. (2014). Aiming to improve the quality of primary mental health care: developing an intervention for underserved communities. *BMC Fam Pract., 16*. doi:10.1186/1471-2296-15-68 | Not a systematic review |
| Clark, D. M. (2015). Improving Access to Psychological Therapies (IAPT) - An Open Gate to Treat Anxiety and Depression. *Verhaltenstherapie, 25*(4), 316-318. | Not a systematic review |
| Claassen, D., Ascoli, M., Berhe, T., & Priebe, S. (2005). Research on mental disorders and their care in immigrant populations: a review of publications from Germany, Italy and the UK. *European Psychiatry: the Journal of the Association of European Psychiatrists, 20*(8), 540-549. | Not a systematic review |
| Codjoe, L., Byrne, M., Lister, M., McGuire, P., & Valmaggia, L. (2013). Exploring perceptions of 'wellness' in Black ethnic minority individuals at risk of developing psychosis. *Behavioural and Cognitive Psychotherapy, 41*(2), 144-161. | Not a systematic review |
| Collinson, D. (2016). Mental health bulletin: 2015-16, annual report. <http://www.content.digital.nhs.uk/catalogue/PUB22561/mhb-1516-ann-rep.pdf> | Not a systematic review |
| Cooper, B. (2005). Immigration and schizophrenia: The social causation hypothesis revisited. *The British Journal of Psychiatry, 186*(5), 361-363. | Not a systematic review |
| Coren, E., Hossain, R., Pardo, P. J., & Bakker, B. (2016). Interventions for promoting reintegration and reducing harmful behaviour and lifestyles in street-connected children and young people. *Cochrane Database of Systematic Reviews, 0*(1). | Not UK or ethnicity |
| Corlett, S. (2012). Policy watch: fighting stigma and discrimination. *Mental Health & Social Inclusion, 16*(3), 112-115. | Not a systematic review |
| Cornwell, J. (1998). Do GPs prescribe antidepressants differently for South Asian patients? *Family Practice, 15*, S16-S18. | Not a systematic review |
| Cowan, C. (2001). The mental health of Chinese people in Britain: An update on current literature. *Journal of Mental Health, 10*(5), 501-511. | Not a systematic review |
| Crombie, I., Falconer, D., Irvine, L., Williams, B., Ricketts, I., Humphris, G., et al. (2013) Reducing alcohol-related harm in disadvantaged men: development and feasibility assessment of a brief intervention delivered by mobile telephone. *Public Health Res, 1*(3). doi:10.3310/phr01030 | Not ethnicity |
| Crosby, S. S. (2013). Primary care management of non-English-speaking refugees who have experienced trauma: a clinical review. *JAMA, 310*(5), 519-528. | Not UK |
| Crowley, C. (2009). The mental health needs of refugee children: A review of literature and implications for nurse practitioners. *Journal of the American Academy of Nurse Practitioners, 21*(6), 322-331. | Not a systematic review |
| Cummins, L. H., Simmons, A. M., & Zane, N. W. S. (2005). Eating disorders in Asian populations: A critique of current approaches to the study of culture, ethnicity, and eating disorders. *American Journal of Orthopsychiatry, 75*(4), 553-574. | Not a systematic review |
| Curtis, S., & Lawson, K. (2000). Gender, ethnicity and self-reported health: the case of African-Caribbean populations in London. *Social Science & Medicine, 50*(3), 365-385. | Not a systematic review |
| Cusack, P., McAndrew, S., Cusack, F., & Warne, T. (2016). Restraining good practice: Reviewing evidence of the effects of restraint from the perspective of service users and mental health professionals in the United Kingdom (UK). *International Journal of Law and Psychiatry, 46*, 20-26. | Not ethnicity |
| Daker-White, G., Beattie, A. M., Gilliard, J., & Means, R. (2002). Minority ethnic groups in dementia care: a review of service needs, service provision and models of good practice. *Aging & Mental Health, 6*(2), 101-108. | Not a systematic review |
| Dalky, H. F. (2012). Mental illness stigma reduction interventions: review of intervention trials. *Western Journal of Nursing Research, 34*(4), 520-547. | Not ethnicity |
| Das-Munshi, J., & Becares, L. (2014). Ethnic density associations for mental health: systematic review with meta-analysis. *PROSPERO*.  <http://www.crd.york.ac.uk/PROSPERO/display_record.asp?ID=CRD42014012992> | Not published (protocol) |
| Davidson, E. M., Liu, J. J., Bhopal, R., White, M., Johnson, M. R. D., Netto, G., . . . Sheikh, A. (2013). Behavior change interventions to improve the health of racial and ethnic minority populations: A tool kit of adaptation approaches. *Milbank Quarterly, 91*(4), 811-851. | Not mental health |
| Davies, D. R. (1999). Welsh psyche: Implications for psychological services. *International Review of Psychiatry, 11*(2), 197-211. | Not a systematic review |
| de Vibe, M., Bjørndal, A., Tipton, E., Hammerstrøm, K. T., & Kowalski, K. (2012). Mindfulness based stress reduction (MBSR) for improving health, quality of life and social functioning in adults. *Campbell Systematic Reviews, 3*. doi:10.4073/csr.2012.3 | Not UK or ethnicity |
| Dein, S. (1997). ABC of mental health. Mental health in a multiethnic society. *BMJ, 315*(7106), 473-476. | Not a systematic review |
| Dennis, M. S., & Lindesay, J. (1995). Suicide in the elderly: The United Kingdom perspective. *International Psychogeriatrics, 7*(2), 263-274. | Not a systematic review |
| Dickerson, F. (2015). Early detection and intervention for people with psychosis: Getting to the bottom line. *Journal of Nervous and Mental Disease, 203*(5), 307-309. | Not a systematic review |
| Dieterich, M., Irving, C. B., Bergman, H., Khokhar, M. A., Park, B., & Marshall, M. (2017). Intensive case management for severe mental illness. *Cochrane Database of Systematic Reviews, 0*(1). | Not ethnicity |
| Dinnen, S., Simiola, V., & Cook, J. M. (2015). Post-traumatic stress disorder in older adults: a systematic review of the psychotherapy treatment literature. *Aging Ment Health, 19(*2), 144-150 | Not UK |
| Doki, S., Sasahara, S., & Matsuzaki, I. (2015). Stress of working abroad.  *PROSPERO*.  <http://www.crd.york.ac.uk/PROSPERO/display_record.asp?ID=CRD42015029315> | Not published (protocol) |
| Dowrick, C., Gask, L., Edwards, S., Aseem, S., Bower, P., Burroughs, H., . . . Waheed, W. (2009). Researching the mental health needs of hard-to-reach groups: managing multiple sources of evidence. *BMC Health Services Research, 9*(1), 226. doi:10.1186/1472-6963-9-226 | Separate UK and ethnicity data, but no UK data by ethnicity |
| Drennan, V. M., & Joseph, J. (2005). Health visiting and refugee families: Issues in professional practice. *Journal of Advanced Nursing, 49*(2), 155-163. | Not a systematic review |
| D'Silva, S., Poscablo, C., Habousha, R., Kogan, M., & Kligler, B. (2012). Mind-body medicine therapies for a range of depression severity: a systematic review. *Psychosomatics, 53*(5), 407-423. | Not ethnicity |
| Ekeland, E., Heian, F., Hagen, K. B., Abbott, J., & Nordheim, L. (2005). Exercise to improve self-esteem in children and young people. *Campbell Systematic Reviews, 4*. doi:10.4073/csr.2005.4 | Not UK or ethnicity |
| Epelbaum, C., Trejo, E., Taylor, E. R., Dekleva, K., & Mills, B. (2010). Immigration trauma, substance abuse, and suicide. *Harvard Review of Psychiatry, 18*(5), 304-313. | Not a systematic review |
| Escobar, J. I., & Gureje, O. (2007). Influence of cultural and social factors on the epidemiology of idiopathic somatic complaints and syndromes. *Psychosomatic Medicine, 69*(9), 841-845. | Not UK |
| Evans-Lacko, S., Courtin, E., Fiorillo, A., Knapp, M., Luciano, M., Park, A. L., . . . Thornicroft, G. (2014). The state of the art in European research on reducing social exclusion and stigma related to mental health: a systematic mapping of the literature. *European psychiatry: the journal of the Association of European Psychiatrists, 29*(6), 381-389. | Not ethnicity |
| Everson‐Hock, E. S., Jones, R., Guillaume, L., Clapton, J., Duenas, A., Goyder, E., . . . Swann, C. (2011). Supporting the transition of looked‐after young people to independent living: A systematic review of interventions and adult outcomes. *Child: Care, Health and Development, 37*(6), 767-779. | Not ethnicity |
| Fajutrao, L., Locklear, J., Priaulx, J., & Heyes, A. (2009). A systematic review of the evidence of the burden of bipolar disorder in Europe. *Clinical Practice and Epidemiology in Mental Health, 5*. | Not ethnicity |
| Farmer, P., & Dyer, J. (2016). The Five Year Forward View for mental health: a report from the independent Mental Health Taskforce to the NHS in England. <https://www.england.nhs.uk/wp-content/uploads/2016/02/Mental-Health-Taskforce-FYFV-final.pdf> | Not a systematic review |
| Faulkner, A. (2014). Ethnic inequalities in mental health: promoting lasting positive change: a consultation with black and minority ethnic mental health service users*.* Network for Mental Health, & Lankelly Chase Foundation. <http://www.nsun.org.uk/assets/downloadableFiles/EthnicInequalitiesinMentalHealthReportFebruary20142.pdf> | Not a systematic review |
| Fazel, S., Fiminska, Z., & Cocks, C. (2016). Patient outcomes following discharge from secure psychiatric hospitals: systematic review and meta-analysis. *The British Journal of Psychiatry*, 208(1), 17-25. | Not ethnicity |
| Fazel, M., Reed, R., Panter-Brick, C., & Stein, A. (2012). Mental health of displaced and refugee children resettled in high-income countries: risk and protective factors. *The Lancet*, 379(9812), 266-282. | Not a systematic review |
| Fazel, M., & Stein, A. (2002). The mental health of refugee children. *Archives of Disease in Childhood, 87*(5), 366-370. | Not a systematic review |
| Feldman, R. (2006). Primary health care for refugees and asylum seekers: a review of the literature and a framework for services. *Public Health, 120*(9), 809-816. | Not a systematic review |
| Fellmeth, G., Fazel, M., & Plugge, E. (2017). Migration and perinatal mental health in women from low- and middle-income countries: a systematic review and meta-analysis. *BJOG, 124*, 742-752. | Not UK |
| Ferguson, K. M., Wu, Q., Spruijt-Metz, D., & Dyrness, G. (2007). Outcomes evaluation in faith-based social services: are we evaluating faith accurately? *Research on Social Work Practice*, *17*(2), 264-276. | Not UK |
| Fernandez, A., Moreno-Peral, P., Zabaleta-del-Olmo, E., Bellon, J. A. . . . Rubio-Valera, M. (2015). Is there a case for mental health promotion in the primary care setting? A systematic review. *Preventive Medicine*, 76, S5-S11. | Not ethnicity |
| Fernando, S. (2012). Race and culture issues in mental health and some thoughts on ethnic identity. *Counselling Psychology Quarterly, 25*(2), 113-123. | Not a systematic review |
| Fisher, M., & Baum, F. (2010). The social determinants of mental health: Implications for research and health promotion. *Australian and New Zealand Journal of Psychiatry, 44*(12), 1057-1063. | Not UK |
| Fleming, E., , G., & , W. (2009). An exploration of culture, diabetes, and nursing in the South Asian community: A metasynthesis of qualitative studies. *Journal of Transcultural Nursing, 20*(2), 146-155. | Not mental health |
| Ford, J. A., Wong, G., & Jones, A. P. (2016). Access to primary care for socioeconomically disadvantaged older people in rural areas: a realist review. *BMJ Open*, 6:e010652. doi:10.1136/bmjopen-2015-010652 | Not ethnicity |
| Forsythe-Brown, I. (2013). Review of Working with families of African Caribbean origin: Understanding issues around immigration and attachment. *Ethnic and Racial Studies, 36*(7), 1252-1253. | Not a systematic review |
| Foster, J., Papadopoulos, C., Dadzie, L., & Jayasinghe, N. (2007). A review of tobacco and alcohol use literature in the native and migrant Greek community. *Journal of Substance Use, 12*(5), 323-335. | Not a systematic review |
| Franx, G., Kroon, H., Grimshaw, J., Drake, R., Grol, R., & Wensing, M. (2008). Organizational change to transfer knowledge and improve quality and outcomes of care for patients with severe mental illness: a systematic overview of reviews. *Canadian Journal of Psychiatry - Revue Canadienne de Psychiatrie, 53*(5), 294-305. | Not ethnicity |
| Fryers, T., Melzer, D., & Jenkins, R. (2003). Social inequalities and the common mental disorders - A systematic review of the evidence. *Social Psychiatry and Psychiatric Epidemiology, 38*(5), 229-237. | Not ethnicity |
| Fuentes, D., & Aranda, M. P. (2012). Depression interventions among racial and ethnic minority older adults: a systematic review across 20 years. *American Journal of Geriatric Psychiatry, 20*(11), 915-931. | Not UK |
| Fung, W. L. A., Bhugra, D., & Jones, P. B. (2009). Ethnicity and mental health: the example of schizophrenia and related psychoses in migrant populations in the Western world. *Psychiatry, 8*(9), 335-341. | Not a systematic review |
| Gask, L., Bower, P., Lamb, J., Burroughs, H., Chew-Graham, C., Edwards, S., . . . Group, A. M. P. R. (2012). Improving access to psychosocial interventions for common mental health problems in the United Kingdom: narrative review and development of a conceptual model for complex interventions. *BMC Health Services Research, 12*, 249. | Not a systematic review |
| Gelder, U. (2006). Review of The well-being of children in the UK. *Early Years: An International Journal of Research and Development, 26*(1), 111-112. | Not a systematic review |
| Gensby, U., Lund, T., Kowalski, K., Saidj, M., Jørgensen, A. M. K., Filges, T., Irvin, E., Amick, B. C. III, & Labriola, M. (2012). Workplace Disability Management Programs Promoting Return-to-Work: A Systematic Review. *Campbell Systematic Reviews, 17*. doi:10.4073/csr.2012.17 | Not UK or ethnicity |
| Gilburt, H., Peck, E., Ashton, B., Edwards, N., & Naylor, C. (2014). Service  transformation: lessons from mental health. King’s Fund. <https://www.kingsfund.org.uk/sites/files/kf/field/field_publication_file/service-transformation-lessons-mental-health-4-feb-2014.pdf> | Not a systematic review |
| Gray, R., Smedley, N., & Thomas, B. (1997). The use of section 136: a review of research 1972-96. *Psychiatric Care, 4*(2), 62-66. | Not a systematic review |
| Greenhalgh, J., Knight, C., Hind, D., Beverley, C., & Walters, S. (2005). Clinical and cost-effectiveness of electroconvulsive therapy for depressive illness, schizophrenia, catatonia and mania; systematic reviews and economic modelling studies. *Health Technol Assess, 9*(9). | Not ethnicity |
| Greig, R. (2006). Government policy in England: inclusion in mainstream healthcare. *Psychiatry, 5*(9), 295-297. | Not a systematic review |
| Grey, H. (2016). Prevalence and outcomes of adverse childhood experiences (ACEs) in young offender populations. *PROSPERO*.  <http://www.crd.york.ac.uk/PROSPERO/display_record.asp?ID=CRD42016051740> | Not published (protocol) |
| Grey, T., Sewell, H., Shapiro, G., & Ashraf, F. (2013). Mental health inequalities facing U.K. minority ethnic populations: Causal factors and solutions. *Journal of Psychological Issues in Organizational Culture, 3*, 146-157. | Not a systematic review |
| Guo, B., & Harstall, C. (2002). Efficacy of suicide prevention programs for children and youth. *Health Technology Assessment, 26*(Series A). Edmonton: Alberta Heritage Foundation for Medical Research. | Not UK |
| Hackett, L., Theodosiou, L., & Patel, J. (2006). Service innovations: Developing a service for the mental health needs of South-Asian children and adolescents. *Psychiatric Bulletin, 30*(12), 460-462. | Not a systematic review |
| Haeri, S., Williams, J., Kopeykina, I., Johnson, J., Newmark, A., Cohen, L., & Galynker, I. (2011). Disparities in diagnosis of bipolar disorder in individuals of African and European descent: A review. *Journal of Psychiatric Practice, 17*(6), 394-403. | Not a systematic review |
| Hall, E. (2005). The entangled geographies of social exclusion/inclusion for people with learning disabilities. *Health and Place, 11*(2), 107-115. | Not ethnicity |
| Harkes, M. A., Brown, M., & Horsburgh, D. (2014). Self Directed Support and people with learning disabilities: a review of the published research evidence. *British Journal of Learning Disabilities, 42*(2), 87-101. | Not ethnicity |
| Hashemi, A. H., & Cochrane, R. (1999). Expressed emotion and schizophrenia: A review of studies across cultures. *International Review of Psychiatry, 11*(2), 219-224. | Not a systematic review |
| Haw, C., & Hawton, K. (2016). Suicide and Self-Harm by Drowning: A Review of the Literature. *Archives of Suicide Research, 20*(2), 95-112. | Separate UK and ethnicity data, but no UK data by ethnicity |
| Healey, P., et al. (2017). Cultural adaptations to augment health and mental health services: a systematic review. *BMC Health Serv Res., 17*, doi:10.1186/s12913-016-1953-x | Not UK |
| Health Committee (2015). Healthy minds, healthy Londoners: improving access to mental health services for London’s young and Black, Asian and minority ethnic population. <https://www.london.gov.uk/sites/default/files/gla_migrate_files_destination/HealthyMindsHealthyLondoners_0.pdf> | Not a systematic review |
| Hek, R., Hughes, N., & Ozman, R. (2012). Safeguarding the Needs of Children and Young People Seeking Asylum in the UK: Addressing Past Failings and Meeting Future Challenges. *Child Abuse Review, 21*(5), 335-348. | Not a systematic review |
| Hickling, F. W. (2005). The epidemiology of schizophrenia and other common mental health disorders in the English-speaking Caribbean. *Pan American Journal of Public Health, 18*(4-5), 256-262. | Not a systematic review |
| HFMA, Mental Health Finance Faculty, & NHS Providers (2016). Funding mental health at local level: unpicking the variation. <https://nhsproviders.org/media/1945/nhs-providers_hfma_mental-health-survey.pdf> | Not ethnicity |
| Hickling, F. W. (1993). Psychiatry in Jamaica growth and development. *International Review of Psychiatry, 5*(2), 193-203. | Not UK |
| Higginbottom, G. M. A., Richter, M. S., Mogale, R. S., Ortiz, L., Young, S., & Mollel, O. (2011). Identification of nursing assessment models/tools validated in clinical practice for use with diverse ethno-cultural groups: an integrative review of the literature. *BMC Nursing*, *10*. doi:10.1186/1472-6955-10-16 | Not UK |
| Hines, L. A., Sundin, J., Rona, R. J., Wessely, S., & Fear, N. T. (2014). Posttraumatic stress disorder post Iraq and Afghanistan: Prevalence among military subgroups. *The Canadian Journal of Psychiatry / La Revue canadienne de psychiatrie, 59*(9), 468-479. | Not UK |
| Hitchcock, C., Werner-Seidler, A., Blackwell, S. E., & Dalgleish, T. (2017). Autobiographical episodic memory-based training for the treatment of mood, anxiety and stress-related disorders: A systematic review and meta-analysis. *Clinical Psychology Review, 52*, 92-107. | Not UK or ethnicity |
| Ho, E. C., Chiu, H. F., Chong, M. Y., Yu, X., Kundadak, G., & Kua, E. H. (2012). Elderly suicide in Chinese populations. *Asia-Pacific Psychiatry, 4*(1), 5-9. | Not a systematic review |
| Hodes, M. (2000). Psychological distressed refugee children in the United Kingdom. *Child Psychology & Psychiatry Review, 5*(2), 57-68. | Not a systematic review |
| Holttum, S. (2012). Research watch: recovery as a personal journey: how mental health services are trying to support it. *Mental Health & Social Inclusion, 16*(4), 169-174. | Not a systematic review |
| Hossain, M., Dewey, A., Hakak, Y., & Jutlla, K. (2013). Understanding dementia among UK Bangladeshi: a synthesis of qualitative research on South Asian people with dementia. *PROSPERO*. <https://researchportal.port.ac.uk/portal/files/240901/Understanding_dementia_among_UK_Bangladeshi_a_synthesis_of_qualitative_research_on_South_Asian_people_with_dementia.pdf> | Not published (protocol) |
| Howard, M., & Hodes, M. (2000). Psychopathology, adversity, and service utilization of young refugees. *Journal of the American Academy of Child & Adolescent Psychiatry, 39*(3), 368-377. | Not a systematic review |
| Hunter, E. C. M., Sierra, M., & David, A. S. (2004). The epidemiology of depersonalisation and derealisation - A systematic review. *Social Psychiatry and Psychiatric Epidemiology, 39*(1), 9-18. | Not ethnicity |
| Husain, M. I., Waheed, W., & Husain, N. (2006). Self-harm in British South Asian women: Psychosocial correlates and strategies for prevention. *Annals of General Psychiatry, 5*(7). | Not a systematic review |
| Husk, K., Lovell, R., Cooper, C., Stahl-Timmins, W., & Garside, R. (2016). Participation in environmental enhancement and conservation activities for health and well-being in adults: a review of quantitative and qualitative evidence. *Cochrane Database of Systematic Reviews, 0*(5). | Not ethnicity |
| Hussain, F., & Cochrane, R. (2004). Depression in South Asian women living in the UK: a review of the literature with implications for service provision. *Transcultural Psychiatry, 41*(2), 253-270. | Not a systematic review |
| Hutchinson, G., & Haasen, C. (2004). Migration and schizophrenia: the challenges for European psychiatry and implications for the future. *Social Psychiatry & Psychiatric Epidemiology, 39*(5), 350-357. | Not a systematic review |
| Hutchinson, G., & Hickling, F. W. (1999). Problem in society, problem in psychiatry. *International Review of Psychiatry, 11*(2), 162-167. | Not a systematic review |
| Hutchinson, G., Mallett, R., & Fletcher, H. (1999). Are the increased rates of psychosis reported for the population of Caribbean origin in Britain an urban effect? *International Review of Psychiatry, 11*(2), 122-128. | Not a systematic review |
| Hutchinson, G., & Sharpley, M. (1999). Ethnicity and first year of contact with psychiatric services. *The British Journal of Psychiatry, 175*, 492-492. | Not a systematic review |
| Ibbotson, B. (2010). Review of Mental health, service user involvement and recovery. *International Journal of Psychiatry in Clinical Practice, 14*(3), 233-234. | Not a systematic review |
| Ida, D. J. (2007). Cultural competency and recovery within diverse populations. *Psychiatric Rehabilitation Journal, 31*(1), 49-53. | Not a systematic review |
| Inclusion Health (2014). Promising practice: enabling better access to primary care for vulnerable populations: examples of good practice. <https://www.gov.uk/government/uploads/system/uploads/attachment_data/file/307376/Promising_Practice.pdf?utm_medium=email&utm_source=The+King%27s+Fund+newsletters&utm_campaign=4043517_HMP+2014-05-02&dm_i=21A8,2ENZX,FLXH0G,8RQGI,1> | Not ethnicity |
| Independent Mental Health Services Alliance (2015). Breaking down barriers: improving patient access and outcomes in mental health. <http://www.imhsa.org.uk/upload/files/IMHSA_policypapers_30_790927026.pdf> | Not ethnicity |
| Ineichen, B. (2008). Suicide and attempted suicide among South Asians in England: Who is at risk? *Mental Health in Family Medicine, 5*(3), 135-138. | Not a systematic review |
| Ineichen, B. (2012). Mental illness and suicide in British South Asian adults. *Mental Health, Religion & Culture, 15*(3), 235-250. | Not a systematic review |
| Ivbijaro, G. O., Kolkiewicz, L. A., & Palazidou, E. (2005). Mental health in primary care: ways of working -- the impact of culture. *Primary Care Mental Health, 3*(1), 47-53. | Not a systematic review |
| Jones, K. (1967). Review of Mental health in the service of the community. *The Sociological Review, 15*(2), 228-229. | Not a systematic review |
| Jones, P., Russo, D., Stochl, J., Shelley, G., Crane, C., Painter, M., et al. (2016) Understanding causes of and developing effective interventions for schizophrenia and other psychoses. *Programme Grants Appl Res*, *4*(02). doi:10.3310/pgfar04020 | Not a systematic review |
| Jukes, M., & O'Shea, K. (1998). Mental health. Transcultural therapy 1: mental health and learning disabilities. *British Journal of Nursing, 7*(15), 901-906. | Not a systematic review |
| Jun, G. T., Morrison, C., & Clarkson, P. J. (2014). Articulating current service development practices: a qualitative analysis of eleven mental health projects. *BMC Health Services Research*, *14*. doi:10.1186/1472-6963-14-20 | Not ethnicity |
| Jutlla, K. (2013). Ethnicity and cultural diversity in dementia care: a review of the research. *Journal of Dementia Care, 21*(2), 33-39. | Not a systematic review |
| Kaltenthaler, E., Brazier, J., de Nigris, E., Tumur, I., & Ferriter, M. (2006). Computerised cognitive behaviour therapy for depression and anxiety update: a systematic review and economic evaluation. *Health Technology Assessment, 10*(33), 186. doi:10.3310/hta10330 | Not ethnicity |
| Kaltenthaler, E., Pandor, A., & Wong, R. (2014). The effectiveness of sexual health interventions for people with severe mental illness: a systematic review. *Health Technology Assessment (Winchester, England), 18*(1), 1-74. | Not UK |
| Kaplan, I., Stolk, Y., Valibhoy, M., Tucker, A., & Baker, J. (2016). Cognitive assessment of refugee children: effects of trauma and new language acquisition. *Transcultural Psychiatry, 53*(1), 81-109. | Not a systematic review |
| Katona, C., Robjant, K., von Werthern, M., Chui, Z., Schon, R. (2017).  Immigration detention and mental health: a systematic review. *PROSPERO*. <http://www.crd.york.ac.uk/PROSPERO/display_record.asp?ID=CRD42017056444> | Not published (protocol) |
| Kendrick, T. (2003). Review: black people are more likely than white people to be detained in psychiatric wards in the United Kingdom. *Evidence Based Mental Health, 6*(3), 76-76. | Not a systematic review |
| Kalra, G., & Bhugra, D. (2011). Ethnic factors in managing black and minority ethnic patients. *Current opinion in psychiatry, 24*(4), 313-317. | Not a systematic review |
| Karriker-Jaffe, K. J. (2011). Areas of disadvantage: a systematic review of effects of area-level socioeconomic status on substance use outcomes. *Drug & Alcohol Review, 30*(1), 84-95. | Not ethnicity |
| Kasmi, Y. (2007). Characteristics of patients admitted to psychiatric intensive care units. *Irish Journal of Psychological Medicine, 24*(2), 75-78. | Not a systematic review |
| Kemp, M. (2003). Review of Mental Slavery: Psychoanalytic Studies of Caribbean People. *Journal of the British Association of Psychotherapists (BAP), 41*(1), 72-80. | Not a systematic review |
| Kennedy, P., Kilvert, A., & Hasson, L. (2015). Ethnicity and rehabilitation outcomes: The Needs Assessment Checklist. *Spinal Cord, 53*(5), 334-339. | Not a systematic review |
| Khan, F., & Waheed, W. (2006). Suicide and self-harm in South Asian immigrants. *Psychiatry, 5*(8), 283-285. | Not a systematic review |
| Kisely, S. R., Campbell, L. A., & O'Reilly, R. (2017). Compulsory community and involuntary outpatient treatment for people with severe mental disorders. *Cochrane Database of Systematic Reviews, 0*(3). | Not UK |
| Krentzman, A. R. (2013). Review of the application of positive psychology to substance use, addiction, and recovery research. *Psychology of Addictive Behaviors, 27*(1), 151-165. | Not ethnicity |
| Kubitz, N., Vossen, C., Papadimitropoulou, K., & Karabis, A. (2014). The prevalence and disease burden of treatment-resistant depression-a systematic review of the literature. *Value in Health, 17*(7), A455-A456. | Wrong publication type |
| Laird, L. D., Amer, M. M., Barnett, E. D., & Barnes, L. L. (2007). Muslim patients and health disparities in the UK and the US. *Archives of Disease in Childhood, 92*(10), 922-926. | Not a systematic review |
| Lankelly Chase Foundation, Mind, The Afia Trust, & Centre for Mental Health (2014). Ethnic inequalities in mental health: promoting lasting positive change. <https://lankellychase.org.uk/wp-content/uploads/2015/07/Ethnic-Inequality-in-Mental-Health-Confluence-Full-Report-March2014.pdf> | Not a systematic review |
| Lau, A. (1990). Psychological problems in adolescents from ethnic minorities. *British Journal of Hospital Medicine, 44*(3), 201-205. | Not a systematic review |
| Laungani, P. (1999). Understanding mental illness in India and Britain. *International Journal of Health Promotion & Education, 37*(4), 144-157. | Wrong publication type |
| Lay, A. Y., & Ridge, M. (2011). Addressing the impact of social exclusion on mental health in Gypsy, Roma, and Traveller communities. *Mental Health & Social Inclusion, 15*(3), 128-137. | Not a systematic review |
| Leaune, E., Dealberto, M.-J., Brunelin, J., Poulet, E., & Zeroug-Vial, H. (2016). Defining the role of ethnicity in the psychosis continuum: a systematic review and meta-analysis. *PROSPERO*. <http://www.crd.york.ac.uk/PROSPERO/display_record.asp?ID=CRD42016047742> | Not published (protocol) |
| Leavey, G. (1999). Suicide and Irish migrants in Britain: Identity and integration. *International Review of Psychiatry, 11*(2), 168-172. | Not a systematic review |
| Lefley, H. P. (1985). Families of the mentally ill in cross-cultural perspective. *Psychosocial Rehabilitation Journal, 8*(4), 57-75. | Not a systematic review |
| Lefley, H. P. (2012). Cross-cultural perspective of family psychoeducation. Psychiatric Annals, 42(6), 236-240. | Not a systematic review |
| Levy, R. (2010). Medication use by ethnic and racial groups: Policy implications. *Journal of Pharmaceutical Health Services Research, 1*(1), 15-22. | Not UK |
| Li, H., Song, X., & Yu, X. (2012). A review of consequences of relapse in schizophrenia. *Value in Health, 15*(7), A670. | Not a systematic review |
| Littlewood, R. (2015). Review of Insanity, race and colonialism: Managing mental disorder in the post-emancipation British Caribbean, 1838–1914.*The British Journal of Psychiatry, 206*(4), 345-345. | Not a systematic review |
| Livanou, M., Furtado, V., Silvester, A., & Singh, S. (2015). Prevalence of mental health problems among young offenders in custody and community: a meta-analysis. *PROSPERO*. <http://www.crd.york.ac.uk/PROSPERO/display_record.asp?ID=CRD42015029677> | Not published (protocol) |
| Livingston, J. D. (2016). Contact between police and people with mental disorders: a review of rates. *Psychiatric Services, 67*(8), 850-857. | Separate UK and ethnicity data, but no UK data by ethnicity |
| Livingston, G., & Sembhi, S. (2003). Mental health of the ageing immigrant population. *Advances in Psychiatric Treatment, 9*(1), 31-37. | Not a systematic review |
| Lloyd, K. (2009). Common mental disorders among black and minority ethnic groups in the UK. *Psychiatry, 8*(9), 342-346. | Not a systematic review |
| Lovrin, M. (2009). Treatment of major depression in adolescents: Weighing the evidence of risk and benefit in light of black box warnings. *Journal of Child and Adolescent Psychiatric Nursing, 22*(2), 63-68. | Not ethnicity |
| Lucas, P. J., McIntosh, K., Petticrew, M., Roberts, H. M., & Shiell, A. (2008). Financial benefits for child health and well-being in low income or socially disadvantaged families in developed world countries. *Campbell Systematic Reviews, 9*. doi:10.4073/csr.2008.9 | Not UK or ethnicity |
| Macdonald, G., Bennett, C., Dennis, J. A., Coren, E., Patterson, J., Astin, M., & Abbott, J. (2008). Home-based support for disadvantaged teenage mothers. *Cochrane Database of Systematic Reviews, 0*(1). | Not UK |
| MacDonald, G.M., & Turner, W. (2007). Treatment foster care for improving outcomes in children and young people. *Campbell Systematic Reviews, 9*. doi:10.4073/csr.2007.9 | Not UK |
| Macgregor, S., & Thickett, A. (2011). Partnerships and communities in English drug policy: The challenge of deprivation. *International Journal of Drug Policy, 22*(6), 478-490. | Not a systematic review |
| MacLean, S., Cameron, J., Harney, A., & Lee, N. K. (2012). Psychosocial therapeutic interventions for volatile substance use: a systematic review. *Addiction, 107*(2), 278-288. | Not ethnicity |
| Maddern, S. (2004). Post-traumatic stress disorder in asylum seekers. *Nursing Standard, 18*(18), 36-39. | Not a systematic review |
| Mahr, F., McLachlan, N., Friedberg, R. D., Mahr, S., & Pearl, A. M. (2015). Cognitive-behavioral treatment of a second-generation child of Pakistani descent: ethnocultural and clinical considerations. *Clinical Child Psychology* *and Psychiatry, 20*(1), 134-147. | Not a systematic review |
| March, D., Hatch, S. L., & Susser, E. (2010). Psychosis in migrant and minority populations: Prescriptions for scientific and social policy. *Psychological Medicine, 40*(5), 737-739. | Not a systematic review |
| Markkula, N., Cabieses, B., Lehti, V., Uphoff, N., Maturana, A., Pacheco, C., & Jasmen, A. (2016). Systematic review: use of health services among international migrant children. *PROSPERO*.  <http://www.crd.york.ac.uk/PROSPERO/display_record.asp?ID=CRD42016039876> | Not published (protocol) |
| Martin, G. (2009). Recovery approach to the care of people with dementia: decision making and 'best interests' concerns. *Journal of Psychiatric & Mental Health Nursing, 16*(7), 654-660. | Not ethnicity |
| Masley, S. A., Gillanders, D. T., Simpson, S. G., & Taylor, M. A. (2012). A Systematic Review of the Evidence Base for Schema Therapy. *Cognitive Behaviour Therapy, 41*(3), 185-202. | Not ethnicity |
| Mathers, B. M., Degenhardt, L., Ali, H., Wiessing, L., Hickman, M., Mattick, R. P., . . . Strathdee, S. A. (2010). HIV prevention, treatment, and care services for people who inject drugs: a systematic review of global, regional, and national coverage. *The Lancet, 375*(9719), 1014-1028. | Not ethnicity |
| Maynard, B. R., Solis, M. R., Miller, V. L., & Brendel, K. E. (2017). Mindfulness-based interventions for improving cognition, academic achievement, behavior, and socioemotional functioning of primary and secondary school students. *Campbell Systematic Reviews, 5*. doi:10.4073/csr2017.5 | Not ethnicity |
| McCourt, J., & Waller, G. (1996). The influence of sociocultural factors on the eating psychopathology of Asian women in British society. *European Eating Disorders Review, 4*(2), 73-83. | Not a systematic review |
| McGee, T. F. (1976). Review of Community mental health programs in England: An American view. *American Journal of Orthopsychiatry, 46*(3), 562-563. | Not a systematic review |
| McGruder, J. (2004). Disease Models of Mental Illness and Aftercare Patient Education: Critical Observations from Meta-Analyses, Cross-Cultural Practice and Anthropological Study. *British Journal of Occupational Therapy, 67*(7), 310-318. | Not a systematic review |
| McKeigue, P. M., & Karmi, G. (1993). Alcohol consumption and alcohol-related problems in Afro-Caribbeans and south Asians in the United Kingdom. *Alcohol & Alcoholism, 28*(1), 1-10. | Not a systematic review |
| McPherson, K. E., Kerr, S., Morgan, A., McGee, E., Cheater, F. M., McLean, J., & Egan, J. (2013). The association between family and community social capital and health risk behaviours in young people: an integrative review. *BMC Public Health, 13*. doi:10.1186/1471-2458-13-971. | Separate UK and ethnicity data, but no UK data by ethnicity |
| Meekums, B., Karkou, V., & Nelson, E. A. (2015). Dance movement therapy for depression. *Cochrane Database of Systematic Reviews, 0*(2). | Not ethnicity |
| Mela, M., & McBride, A. J. (2000). Khat and khat misuse: an overview. *Journal of Substance Use, 5*(3), 218-226. | Not a systematic review |
| Mental Health Crisis Care Concordat (2014). Improving outcomes  for people experiencing mental health crisis. <http://www.crisiscareconcordat.org.uk/wp-content/uploads/2014/04/36353_Mental_Health_Crisis_accessible.pdf> | Not a systematic review |
| Mental Health Providers Forum and Race Equality Foundation (2015). Better practice in mental health for black and minority ethnic communities. <http://qna.files.parliament.uk/qna-attachments/516557/original/Better%20practice%20in%20mental%20health%20(2)%20(1).pdf> | Not a systematic review |
| Milne, A., Culverwell, A., Guss, R., Tuppen, J., Whelton, R., Milne, A., . . . Whelton, R. (2008). Screening for dementia in primary care: a review of the use, efficacy and quality of measures. *International Psychogeriatrics, 20*(5), 911-926. | Not a systematic review |
| Mind (2015). Our communities, our mental health: commissioning for better public mental health. <https://www.mind.org.uk/media/2976113/mind_public-mental-health-guide_web-version.pdf> | Not a systematic review |
| Minnis, H., Kelly, E., Bradby, H., Oglethorpe, R., Raine, W., & Cockburn, D. (2003). Cultural and language mismatch: Clinical complications. *Clinical Child Psychology and Psychiatry, 8*(2), 179-186. | Not a systematic review |
| Mitchell, J. (2015). Supporting refugee children who arrive in your schools. *British Journal of School Nursing, 10*(9), 452-456. | Not a systematic review |
| Mollon, P. (2007). Thought field therapy and its derivatives: Rapid relief of mental health problems through tapping on the body. *Primary Care and Community Psychiatry, 12*(3), 123-127. | Not ethnicity |
| Mooney, R., Trivedi, D., & Sharma, S. (2016). How do people of South Asian origin understand and experience depression? A protocol for a systematic review of qualitative literature. *BMJ Open*, *6*(8), e011697. | Not published (protocol) |
| Moriarty, J. (2014). Personalisation for people from black and minority ethnic groups. (Better Health Briefing; No. 34). Race Equality Foundation. | Not a systematic review |
| Mottron, L. (2004). Matching Strategies in Cognitive Research with Individuals with High-Functioning Autism: Current Practices, Instrument Biases, and Recommendations. *Journal of Autism and Developmental Disorders, 34*(1), 19-27. | Not a systematic review |
| Murray, J., Farrington, D., Sekol, I., & Olsen, R. F. (2009). Effects of parental imprisonment on child antisocial behaviour and mental  health: a systematic review. *Campbell Systematic Reviews, 4*. doi:10.4073/csr.2009.4 | Not ethnicity |
| Murray, K., Leske, S., Schweitzer, R., Senadeera, M., & Correa-Velez, I.  (2017). Resilience of asylum seeker and refugee youth after resettlement. *PROSPERO*.  <http://www.crd.york.ac.uk/PROSPERO/display_record.asp?ID=CRD42017055913> | Not published (protocol) |
| Naylor, C., Wallcraft, J., Samele, C., & Greatley, A. (2007). Research Priorities for Service User and Carer-Centred Mental Health Services:  Consultation report. Report for the National Co-ordinating Centre for NHS Service Delivery and Organisation R & D (NCCSDO). | Not a systematic review |
| Nesbitt, A., Lynch, M. A., Nesbitt, A., & Lynch, M. A. (1992). African children in Britain. *Archives of Disease in Childhood, 67*(11), 1402-1405. | Not a systematic review |
| Newbigging, K., Ridley, J., McKeown, M., Machin, K., & Poursanidou, K. (2015). 'When you haven't got much of a voice': An evaluation of the quality of Independent Mental Health Advocate (IMHA) services in England. *Health & Social Care in the Community, 23*(3), 313-324. | Not a systematic review |
| Newby, L., & Denison, N. (eds). (2014). “If you could do one thing...”: nine local actions to reduce health inequalities. British Academy. <http://www.britac.ac.uk/sites/default/files/If%20you%20could%20do%20one%20thing%20-%20full%20report.pdf> | Not a systematic review |
| NICE (2007). Community-based interventions to reduce substance misuse among vulnerable and disadvantaged children and young people. NICE public health intervention guidance 4. <http://www.dldocs.stir.ac.uk/documents/niceonterventions.pdf> | Not a systematic review |
| O'Brien, A., Fahmy, R., & Singh, S. P. (2009). Disengagement from mental health services. A literature review. *Social Psychiatry & Psychiatric Epidemiology, 44*(7), 558-568. | Not a systematic review |
| O'Connor, E., Gaynes, B. N., Burda, B. U., Soh, C., & Whitlock, E. P. (2013). Screening for and treatment of suicide risk relevant to primary care: a systematic review for the U.S. Preventive Services Task Force. *Annals of Internal Medicine, 158*(10), 741-754. | Not ethnicity |
| Okereke, E., Archibong, U., Chiemeka, M., Baxter, C. E., & Davis, S. (2007). Participatory approaches to assessing the health needs of African and African-Caribbean communities. *Diversity in Health & Social Care, 4*(4), 287-301. | Not a systematic review |
| O'Mahony, J., & Donnelly, T. (2010). Immigrant and refugee women's post-partum depression help-seeking experiences and access to care: a review and analysis of the literature. *Journal of Psychiatric & Mental Health Nursing, 17*(10), 917-928. | Not a systematic review |
| Oluwatayo, O., & Gater, R. (2004). The role of engagement with services in compulsory admission of African/Caribbean patients. *Social Psychiatry and Psychiatric Epidemiology, 39*(9), 739-743. | Not a systematic review |
| Onwumere, J., Smith, B., Kuipers, E., Lobban, F., Barrowclough, C., Lobban, F., & Barrowclough, C. (2009). Family intervention with ethnically and culturally diverse groups. *A casebook of family interventions for psychosis., 0*, 211-231. | Not a systematic review |
| Oram, S., Khondoker, M., Abas, M., Broadbent, M., & Howard, L. M. (2015). Characteristics of trafficked adults and children with severe mental illness: A historical cohort study. *The Lancet Psychiatry, 2*(12), 1084-1091. | Not a systematic review |
| Ott, E., & Montgomery, P. (2015). Interventions to improve the economic self-sufficiency and well-being of resettled refugees: a systematic review. *Campbell Systematic Reviews, 4*. doi:10.4073/csr.2015.4 | Not UK |
| Ouellette-Kuntz, H., Garcin, N., Lewis, M. E., Minnes, P., Martin, C., & Holden, J. J. (2005). Addressing health disparities through promoting equity for individuals with intellectual disability. *Canadian Journal of Public Health. Revue Canadienne de Sante Publique, 96*, S8-22. | Not a systematic review |
| Owuro, J. O. A., & Nake, J. N. (2015). Internalised stigma as a barrier to access to health and social care services by minority ethnic groups in the UK. *Better Health Briefing.* <http://www.better-health.org.uk/sites/default/files/briefings/downloads/Health%20Briefing%2036_1.pdf> | Not a systematic review |
| Pinfold, V., Farmer, P., Rapaport, J., Bellringer, S., Huxley, P., Murray, J., Banerjee, S., Slade, M., Kuipers, E., Bhugra, D., & Waitere, S. (2005). Positive and Inclusive? Effective ways for professionals to involve carers in information sharing. Report to the National Co-ordinating Centre for NHS Service Delivery and Organisation R & D (NCCSDO). | Not a systematic review |
| Pinto, R., Ashworth, M., & Jones, R. (2008). Schizophrenia in black Caribbeans living in the UK: an exploration of underlying causes of the high incidence rate. *British Journal of General Practice, 58*(551), 429-434. | Not a systematic review |
| Pottie, K., Welch, V., Niragira, O., Medu, O., James, M., & Miller, K. (2014). Do older migrants suffer higher rates of depressive symptomatology and loneliness than older non-migrants? An equity-focused systematic review. *PROSPERO*.  <http://www.crd.york.ac.uk/PROSPERO/display_record.asp?ID=CRD42014009725> | Not published (protocol) |
| Prady, S. L., Pickett, K. E., Gilbody, S., Petherick, E. S., Mason, D., Sheldon, T. A., & Wright, J. (2016). Variation and ethnic inequalities in treatment of common mental disorders before, during and after pregnancy: combined analysis of routine and research data in the Born in Bradford cohort. *BMC Psychiatry, 16*, 99. | Not a systematic review |
| Pridmore, S., Robinson, J., & Ahmadi, J. (2007). Suicide for scrutinizers. *Australasian Psychiatry, 15*(3), 247-248. | Not a systematic review |
| Procter, N. G. (2005). 'They first killed his heart (then) he took his own life'. Part I: A review of the context and literature on mental health issues for refugees and asylum seekers. *International Journal of Nursing Practice, 11*(6), 286-291. | Not a systematic review |
| Public Health England, & NHS England (2015). A guide to community-centred approaches for health and wellbeing: full report. <https://www.gov.uk/government/uploads/system/uploads/attachment_data/file/417515/A_guide_to_community-centred_approaches_for_health_and_wellbeing__full_report_.pdf> | Not ethnicity |
| Pynoos, R. S., Nader, K., Black, D., Kaplan, T., Hendriks, J. H., Gordon, R., . . . Raphael, B. (1993). The impact of trauma on children and adolescents. *International handbook of traumatic stress syndromes., 0*, 535-657. | Not a systematic review |
| Rapaport, J. (2005). Policy swings over thirty-five years of mental health social work in England and Wales 1969-2004. *Practice (09503153), 17*(1), 43-56. | Not ethnicity |
| Ratan, D., Gandhi, D., & Palmer, R. (1998). Eating disorders in British Asians. *International Journal of Eating Disorders, 24*(1), 101-105. doi:10.1002/(SICI)1098-108X(199807)24:1<101::AID-EAT10>3.0.CO;2-3 | Not a systematic review |
| Regan, J. L., Bhattacharyya, S., Kevern, P., & Rana, T. (2013). A systematic review of religion and dementia care pathways in black and minority ethnic populations. *Mental Health, Religion & Culture, 16*(1), 1-15. | Not UK |
| Remes, O., Brayne, C., van der Linde, R., & Lafortune, L. (2016). A systematic review of reviews on the prevalence of anxiety disorders in adult populations. *Brain and Behavior, 6*(7). doi:10.1002/brb3.497 | Not ethnicity |
| Reupert, A. E., Cuff, R., Drost, L., Foster, K., van Doesum, K. T., & van Santvoort, F. (2013). Intervention programs for children whose parents have a mental illness: a review. *Medical Journal of Australia, 199*(3), S18-22. | Not ethnicity |
| Riaz, F., Sang-Ah Park, M., & Golden, K. (2016). Mental health studies of Asian marginalized populations: a mixed method systematic review. *PROSPERO*.  <http://www.crd.york.ac.uk/PROSPERO/display_record.asp?ID=CRD42016046181> | Not published (protocol) |
| Robbins, V., Sandys, M., & Goldblatt, P. (2014). Take the local lead on health inequalities. *Health Service Journal*. <https://www.hsj.co.uk/sectors/commissioning/take-the-local-lead-on-health-inequalities/5076497.article> | Not a systematic review |
| Roberts, D. J., & de Souza, V. C. (2016). A venue-based analysis of the reach of a targeted outreach service to deliver opportunistic community NHS Health checks to 'hard-to-reach' groups. *Public Health*, 137, 176-81. | Not a systematic review |
| Robinson, G. (2017). Dementia and ethnicity. *Nurse Prescribing, 15*(3), 126-132. | Not a systematic review |
| Robinson, N., Lorenc, A., & Liao, X. (2011). The evidence for Shiatsu: a systematic review of Shiatsu and acupressure. *BMC Complementary and Alternative Medicine,* *11,* doi:10.1186/1472-6882-11-88 | Not ethnicity |
| Rowe, J. (2012). Great expectations: A systematic review of the literature on the role of family carers in severe mental illness, and their relationships and engagement with professionals. *Journal of Psychiatric and Mental Health Nursing, 19*(1), 70-82. | Not ethnicity |
| Roy, A., Fountain, J., & Anitha, S. (2008). The social and institutional context of throughcare and aftercare services for prison drug service clients, with a focus on Black and minority ethnic prisoners. *Drugs & Alcohol Today, 8*(4), 14-25. | Not a systematic review |
| Ruddy, R. A., & Dent-Brown, K. (2007). Drama therapy for schizophrenia or schizophrenia-like illnesses. *Cochrane Database of Systematic Reviews, 0*(1). | Not ethnicity |
| Rudolph, C. E. (2002). Searching for a 'true, clear self': Culture and subjectivity in experiences of bulimia. *62*, 3839-3839. | Wrong publication type |
| Salway, S., Carter, L., Powell, K., Turner, D., Mir, G., & Ellison, G. T. H. (2014). Race equality and health inequalities: towards more integrated policy and practice. A Race Equality Foundation Briefing Paper. *Better Health Briefing, 32*. <http://cdn.basw.co.uk/upload/basw_125418-4.pdf> | Not a systematic review |
| Schafheutle, E. I. (2006). Removing Prescription Charges for Patients with Mental Health Disorders: Would it Improve Patient Outcomes in the UK? *Disease Management & Health Outcomes, 14*(3), 139-145. | Not a systematic review |
| Selten, J.-P., Cantor-Graae, E., & Kahn, R. S. (2007). Migration and schizophrenia. *Current Opinion in Psychiatry, 20*(2), 111-115. | Not a systematic review |
| Sen, P., Exworthy, T., & Forrester, A. (2014). Mental health care for foreign national prisoners in England and Wales. *Journal of Mental Health, 23*(6), 333-339. | Not a systematic review |
| Senior, J., & Shaw, J. (2011). The public health implications of United Kingdom offender healthcare policy: A holistic approach to achieve individual and societal gains. *International Journal of Law and Psychiatry, 34*(4), 283-286. | Not ethnicity |
| Shah, A. (2009). Psychiatry of old age and ethnic minority older people in the United Kingdom. *Reviews in Clinical Gerontology, 19*(2), 119-134. doi:10.1017/S0959259809990190 | Not a systematic review |
| Shah, A., Oommen, G., & Koshy, A. (2009). Ethnic elders and their needs. *Psychiatry, 8*(9), 358-362. | Not a systematic review |
| Shah, A., Oommen, G., & Wuntakal, B. (2008). Cross-cultural aspects of dementia. *Psychiatry, 7*(2), 94-97. | Not a systematic review |
| Sharp, T. J. (1997). Cognitive-behaviour therapy: Towards the new millennium! *Behaviour Change, 14*(4), 187-191. | Not a systematic review |
| Sharpley, M., Hutchinson, G., McKenzie, K., & Murray, R. M. (2001). Understanding the excess of psychosis among the African-Caribbean population in England. *The British Journal of Psychiatry, 178*(Suppl40), s60-s68. doi:10.1192/bjp.178.40.s60. | Not a systematic review |
| Shaw, R. J., Atkin, K., Bécares, L., Albor, C. B., Stafford, M., Kiernan, K. E., . . . Pickett, K. E. (2012). Impact of ethnic density on adult mental disorders: Narrative review. *British Journal of Psychiatry, 201*(1), 11-19. | Not a systematic review |
| Shishehgar, S., Gholizadeh, L., DiGiacomo, M., & Davidson, P. M. (2015). The impact of migration on the health status of Iranians: an integrative literature review. *BMC International Health & Human Rights, 15*, 20. | Not a systematic review |
| Shuker, L. (2013). Review of Safeguarding children from abroad: Refugee, asylum‐seeking and trafficked children in the UK. *Child Abuse Review, 22*(3), 223-223. | Not a systematic review |
| Singh, S. P. (Principal Investigator). (2008-2012). Assessing the impact of the Mental Health Act 2007 (AMEND Study). Warwick Medical School. | Not a systematic review |
| Singh, S. P., & Burns, T. (2006). Race and mental health: there is more to race than racism. *BMJ, 333*(7569), 648-651. | Not a systematic review |
| Singh, R., Nichols, W. C., & Nichols, W. C. (2004). Exploring Culture in Practice: A Few Facets of a Training Course. *Family therapy around the world: A festschrift for Florence W. Kaslow., 0*, 87-104. | Not a systematic review |
| Sinha, S., & Warfa, N. (2013). Treatment of eating disorders among ethnic minorities in western settings: A systematic review. *Psychiatria Danubina, 25*, 295-299. | Wrong publication type |
| Soares-Weiser, K., Bravo Vergel, Y., Beynon, S., Dunn, G., & Barbieri, M., et al. (2007). A systematic review and economic model of the clinical effectiveness and cost-effectiveness of interventions for preventing relapse in people with bipolar disorder. *Health Technol Assess*, *11*(39). | Not ethnicity |
| Spector, R. (2001). Is there a racial bias in clinicians' perceptions of the dangerousness of psychiatric patients? A review of the literature. *Journal of Mental Health, 10*(1), 5-15. | Not a systematic review |
| Stacciarini, J.-M. R., Rosa, A., Ortiz, M., Munari, D. B., Uicab, G., & Balam, M. (2012). Promotoras in mental health: a review of English, Spanish, and Portuguese literature. *Family & Community Health, 35*(2), 92-102. | Not UK |
| Stafford, J., & Kirkbride J. (2016). A systematic review of the incidence of late-onset psychosis. *PROSPERO*.  <http://www.crd.york.ac.uk/PROSPERO/display_record.asp?ID=CRD42016035720> | Not published (protocol) |
| Steel, Z., Marnane, C., Iranpour, C., Chey, T., Jackson, J. W., Patel, V., & Silove, D. (2014). The global prevalence of common mental disorders: a systematic review and meta-analysis 1980-2013. *Int J Epidemiol, 43*(2), 476-493. doi:10.1093/ije/dyu038 | Not ethnicity |
| Strassmayr, C., Matanov, A., Priebe, S., et al. (2012). Mental health care for irregular migrants in Europe: barriers and how they are overcome. *BMC Public Health, 12*, doi:10.1186/1471-2458-12-367 | Not a systematic review |
| Suresh, K., & Bhui, K. (2006). Ethnic minority patients' access to mental health services. *Psychiatry, 5*(11), 413-416. | Not a systematic review |
| ter Heide, J.J., Smid, G., Mooren, T., Kleber, R., & Martinmäki, S. (2017). Prevalence of complex post-traumatic stress disorder in trauma-exposed populations: meta-analysis. *PROSPERO*. <http://www.crd.york.ac.uk/PROSPERO/display_record.asp?ID=CRD42017064445> | Not published (protocol) |
| Thomas, S., & Byford, S. (2003). Research with unaccompanied children seeking asylum. *British Medical Journal, 327*(7428), 1400-1402. | Not a systematic review |
| Thompson, A. D., Bartholomeusz, C., & Yung, A. R. (2011). Social cognition deficits and the 'ultra high risk' for psychosis population: a review of literature. *Early intervention in psychiatry, 5*(3), 192-202. | Not ethnicity |
| Thompson, N., & Bhugra, D. (2000). Rates of deliberate self-harm in Asians: Findings and models. *International Review of Psychiatry, 12*(1), 37-43. doi:10.1080/09540260074102 | Not a systematic review |
| Tillman, J. G. (2017). Review of The last asylum: A memoir of madness in our times. *Psychoanalytic Psychology, 34*(1), 134-136. | Not a systematic review |
| Toniolo, I. (2007). Psychiatric disorders in a transcultural setting. *Clinical Neuropsychiatry, 4*(4), 160-178. | Not a systematic review |
| Trejo-Phillips, P. (2014). Interpreters in a psychotherapeutic triad interpreting for torture survivors: An intergrative literature review through a cultural competency lens. *75*. | Not a systematic review |
| Trivedi, R. B., Nieuwsma, J. A., & Williams Jr, J. W. (2011). Examination of the utility of psychotherapy for patients with treatment resistant depression: A systematic review. *Journal of General Internal Medicine, 26*(6), 643-650. | Not ethnicity |
| Truong, M., Paradies, Y., & Priest, N. (2014) Interventions to improve cultural competency in healthcare: a systematic review of reviews. *BMC Health Serv Res., 14*. doi:10.1186/1472-6963-14-99 | Not UK |
| Turner, S. W., & Herlihy, J. (2009). Working with refugees and asylum seekers. *Psychiatry, 8*(8), 322-324. | Not a systematic review |
| Turrini, G., Purgato, M., Ballette, F., Ostuzzi, G., Nosè, M., & Barbui, C. (2017). Mental health conditions in refugees and asylum seekers: umbrella review of prevalence and intervention studies. *PROSPERO*.  <http://www.crd.york.ac.uk/PROSPERO/display_record.asp?ID=CRD42017056338> | Not published (protocol) |
| van Wyk, S., & Schweitzer, R. D. (2014). A systematic review of naturalistic interventions in refugee populations. *Journal of Immigrant & Minority Health, 16*(5), 968-977. | Not UK |
| Varley, C. K. (2006). Treating depression in children and adolescents: what options now? *CNS Drugs, 20*(1), 1-13. | Not ethnicity |
| Veling, W. (2013). Ethnic minority position and risk for psychotic disorders. *Current Opinion in Psychiatry, 26*(2), 166-171. | Not a systematic review |
| Vickers, B. (2005). Cognitive model of the maintenance and treatment of post-traumatic stress disorder applied to children and adolescents. *Clinical Child Psychology & Psychiatry, 10*(2), 217-234. | Not a systematic review |
| Vige, M. (2014). Tackling inequality in mental health care. *Health Service Journal, 124*(6420), 30-31. | Not a systematic review |
| Waldemar, A. K., Arnfred, S. M., Petersen, L., & Korsbek, L. (2016). Recovery-oriented practice in mental health inpatient settings: A literature review. *Psychiatric Services, 67*(6), 596-602. | Not ethnicity |
| Wang, C., Bannuru, R., Ramel, J., Kupelnick, B., Scott, T., & Schmid, C. H. (2010). Tai Chi on psychological well-being: Systematic review and meta-analysis, *BMC Complementary and Alternative Medicine,* *10,* doi:10.1186/1472-6882-10-23 | Not ethnicity |
| Waraich, P., Goldner, E. M., Somers, J. M., & Hsu, L. (2004). Prevalence and incidence studies of mood disorders: a systematic review of the literature. *Canadian Journal of Psychiatry - Revue Canadienne de Psychiatrie, 49*(2), 124-138. | Not ethnicity |
| Warfa, N., Klein, A., Bhui, K., Leavey, G., Craig, T., & Stansfeld, S. A. (2007). Khat use and mental illness: A critical review. *Social Science & Medicine, 65*(2), 309-318. | Not a systematic review |
| Waxler, N. E. (1974). Culture and mental illness: A social labeling perspective. *Journal of Nervous and Mental Disease, 159*(6), 379-395. | Not a systematic review |
| Webber, M., & Huxley, P. (2004). Social exclusion and risk of emergency compulsory admission. A case-control study. *Social Psychiatry and Psychiatric Epidemiology, 39*(12), 1000-1009. | Not a systematic review |
| White, J., Bagnall, A.-M., & Trigwell, J. (2015). Health trainers making a difference to mental health and wellbeing. *Perspectives in Public Health, 135*(3), 130-132. | Not a systematic review |
| Whitehead, D. L., Steptoe, A., Fink, G., & Fink, G. (2010). Prison. *Stress consequences: Mental, neuropsychological and socioeconomic., 0*, 566-571. | Not a systematic review |
| Whitley, R. (2005). Review of Cultural Diversity, Mental Health and Psychiatry: The Struggle Against Racism. *Transcultural Psychiatry, 42*(3), 507-509. | Not a systematic review |
| Williams, P. E., Turpin, G., & Hardy, G. (2006). Clinical Psychology Service Provision and Ethnic Diversity within the UK: A Review of the Literature. *Clinical Psychology & Psychotherapy, 13*(5), 324-338. | Not a systematic review |
| Wimpenny, K., Savin-Baden, M., & Cook, C. (2014). A Qualitative Research Synthesis Examining the Effectiveness of Interventions Used by Occupational Therapists in Mental Health. *British Journal of Occupational Therapy, 77*(6), 276-288. doi:10.4276/030802214X14018723137959 | Not ethnicity |
| Winstead, B. A. (1996). Review of Suicide--The Ultimate Rejection? A Psycho-Social Study. *Contemporary Psychology, 41*(10), 1062-1062. | Not a systematic review |
| Wollscheid, S., Munthe-Kaas, H. M., Hammerstrøm, K. T., & Noonan, E. (2015). Effect of Interventions to Facilitate Communication Between Families or Single Young People with Minority Language Background and Public Services: A Systematic Review. *Campbell Systematic Reviews, 7*. doi:10.4073/csr.2015.7 | Not UK |
| Wu, Y. T., Beiser, A. S., Breteler, M. M. B., Fratiglioni, L., Helmer, C., Hendrie, H. C., . . . Brayne, C. (2017). The changing prevalence and incidence of dementia over time-current evidence. *Nature Reviews Neurology, 13*(6), 327-339. | Not a systematic review |
| Yorke, C. B., Voisin, D. R., Berringer, K. R., & Alexander, L. S. (2016). Cultural factors influencing mental health help-seeking attitudes among Black English-Speaking Caribbean immigrants in the United States and Britain. *Social Work in Mental Health, 14*(2), 174-194. | Not a systematic review |
| Zolnierek, C. D. (2009). Non-psychiatric hospitalization of people with mental illness: systematic review. *Journal of Advanced Nursing, 65*(8), 1570-1583. | Not ethnicity |

Review of pathways primary literature (2012-2017)

Not peer-reviewed article (n=24)

No relevant outcomes (n=18)

Relevant data not by ethnicity (n=10)

No consideration of ethnicity (n=8)

Not adults (n=7)

Not psychosis (n=6)

Duplicate study/sample (n=4)

Not mental health (n=2)

Not UK (n=1)

Wrong comparison (n=1)

| **Reference** | **Main reason for exclusion** |
| --- | --- |
| Aboulghate, A., Abel, G., Elliott, M. N., Parker, R. A., Campbell, J., Lyratzopoulos, G., . . . , M. (2012). Do English patients want continuity of care, and do they receive it? *British Journal of General Practice, 62*(601), e567-575. | No relevant outcomes |
| Ajnakina, O., Morgan, C., Oduola, S., Bourque, F., Valmaggia, L., Dazzan, P., . . . David, A. S. (2014). Pathways to care for young individuals with a first-episode psychosis in South London: Use of prodromal services. *Schizophrenia Research, 153*, S269 | Not peer-reviewed article |
| Ajnakina, O., Vasso, E., Di Forti, M., Kolliakou, A., Trotta,  A., Schoeler, T., . . . Lally, J. (2015). Ethnic variations in  outcome during the 5-years following a first episode of  psychosis. *Schizophrenia Bulletin, 41*, S159. | Not peer-reviewed article |
| Ali, A., Ghosh, S., Strydom, A., & Hassiotis, A. (2016). Prisoners with intellectual disabilities and detention status. Findings from a UK cross sectional study of prisons. *Research in Developmental Disabilities, 53*, 189-197. | No relevant outcomes |
| Bansal, N., Bhopal, R., Netto, G., Lyons, D., Steiner, M. F., & Sashidharan, S. P. (2014). Disparate patterns of hospitalisation reflect unmet needs and persistent ethnic inequalities in mental health care: the Scottish health and ethnicity linkage study. *Ethnicity & Health, 19*(2), 217-239. | Wrong comparison |
| Beezhold, J., , P., ey, A., , P., ey, S., & Parker, J. (2013). Outcomes of 11509 assessments for possible detention under the mental health ACT in norfolk, United Kingdom 2001-2011. *European Psychiatry, 28*. | Not peer-reviewed article |
| Bhavsar, V., Hotopf, M., Maccabe, J., & McGuire, P. (2016). Subclinical psychotic experiences (Pes) and the rate of mental health service use in South London- A survey/health record linkage study. *Early intervention in psychiatry, 10*, 96. | Relevant data not by ethnicity |
| Boydell, J., Onwumere, J., Dutta, R., Bhavsar, V., Hill, N., Morgan, C., . . . Fearon, P. (2014). Caregiving in first-episode psychosis: social characteristics associated with perceived 'burden' and associations with compulsory treatment. *Early intervention in psychiatry, 8*(2), 122-129. | Relevant data not by ethnicity |
| Brown, J. S., Ferner, H., Wingrove, J., Aschan, L., Hatch, S. L., & Hotopf, M. (2014). How equitable are psychological therapy services in South East London now? A comparison of referrals to a new psychological therapy service with participants in a psychiatric morbidity survey in the same London borough. *Social Psychiatry & Psychiatric Epidemiology, 49*(12), 1893-1902. | No relevant outcomes |
| Brown, J. S. L., Evans-Lacko, S., Aschan, L., Henderson, M. J., Hatch, S. L., & Hotopf, M. (2014). Seeking informal and formal help for mental health problems in the community: A secondary analysis from a psychiatric morbidity survey in South London. *BMC Psychiatry, 14*(1). | No relevant outcomes |
| Bruce, M., Cobb, D., Clisby, H., Ndegwa, D., & Hodgins, S. (2014). Violence and crime among male inpatients with severe mental illness: attempting to explain ethnic differences. *Social Psychiatry & Psychiatric Epidemiology, 49*(4), 549-558. | No relevant outcomes |
| Ciufolini, S., Morgan, C., Morgan, K., Fearon, P., Boydell, J., Hutchinson, G., . . . Dazzan, P. (2015). Self esteem and self agency in first episode psychosis: Ethnic variation and relationship with clinical presentation. *Psychiatry Research, 227*(2), 213-218. | No relevant outcomes |
| Connor, C., Birchwood, M., Lester, H., Singh, S., Patterson, P., Palmer, C., & Butterworth, S. (2012). Reducing DUP in first-episode psychosis requires a detailed understanding of care pathways in community and mental health service settings: Data from Birmingham, UK. *Early intervention in psychiatry, 6*, 5. | Not peer-reviewed article |
| Cooper, C., Spiers, N., Livingston, G., Jenkins, R., Meltzer, H., Brugha, T., . . . Bebbington, P. (2013). Ethnic inequalities in the use of health services for common mental disorders in England. *Social Psychiatry & Psychiatric Epidemiology, 48*(5), 685-692. | Not psychosis |
| Corrigall, R., & Bhugra, D. (2013). The role of ethnicity and diagnosis in rates of adolescent psychiatric admission and compulsory detention: a longitudinal case-note study. *Journal of the Royal Society of Medicine, 106*(5), 190-195. | Not adults |
| Cullen, A. E., Bowers, L., Khondoker, M., Pettit, S., Achilla, E., Koeser, L., . . . Tulloch, A. D. (2016). Factors associated with use of psychiatric intensive care and seclusion in adult inpatient mental health services. *Epidemiology and Psychiatric Sciences*, 1-11. | No relevant outcomes |
| De La Cruz, L. F., Jassi, A., Kolvenbach, S., Vidal-Ribas, P., Llorens, M., & Mataix-Cols, D. (2015). Children from ethnic minorities with obsessive-compulsive disorder: Service use inequalities, reasons behind these inequalities, and treatment outcomes. *European Child and Adolescent Psychiatry, 24*(1), S94. | Not adults |
| De La Cruz, L.F., Llorens, M., Jassi, A., Krebs, G., Vidal-Ribas, P., . . . Mataix-Cols, D. (2015). Ethnic inequalities in the use of secondary and tertiary mental health services among patients with obsessive-compulsive disorder. *British Journal of Psychiatry, 207*(6), 530-535. | Not psychosis |
| Dominguez Barrera, M. D. G., Fisher, H., Johnson, S., & Hodes, M. (2012). Key determinants of longer duration of untreated psychosis in adolescents. *Neuropsychiatrie de l'Enfance et de l'Adolescence, 60*(5), S42. | Not peer-reviewed article |
| Dominguez, M. D., Fisher, H. L., Major, B., Chisholm, B., Rahaman, N., Joyce, J., . . . Hodes, M. (2013). Duration of untreated psychosis in adolescents: ethnic differences and clinical profiles. *Schizophrenia Research, 150*(2), 526-532. | Not adults |
| Dominguez, M. D. G., Fisher, H. L., Johnson, S., & Hodes, M. (2013). Differential pathways to care in first episode psychosis: Adolescents versus adults. *European Child and Adolescent Psychiatry, 22*(2), S167. | Not peer-reviewed article |
| Dominguez, M. D. G., Fisher, H. L., Johnson, S., & Hodes, M. (2015). The influence of family factors in shaping pathways to care in first episode psychosis in adolescents. *European Child and Adolescent Psychiatry, 24*(1), S98. | Not peer-reviewed article |
| Edbrooke-Childs, J., Newman, R., Fleming, I., Deighton, J., & Wolpert, M. (2016). The association between ethnicity and care pathway for children with emotional problems in routinely collected child and adolescent mental health services data. *European Child & Adolescent Psychiatry, 25*(5), 539-546. | Not adults |
| Forrester, A., Singh, J., Slade, K., Exworthy, T., & Sen, P. (2014). Mental health in-reach in an urban UK remand prison. *International journal of prison health., 10*(3), 155-163. | Relevant data not by ethnicity |
| Gaynor, K., & Brown, J. S. L. (2013). Self-referrers to community workshops: Who are they and why do some participants not consult with their GP about their mental health difficulties? *Journal of Mental Health, 22*(3), 227-236. | Not psychosis |
| Gazard, B., Frissa, S., Nellums, L., Hotopf, M., & Hatch, S. L. (2015). Challenges in researching migration status, health and health service use: an intersectional analysis of a South London community. *Ethnicity & Health, 20*(6), 564-593. | Not psychosis |
| Gilbert, C., Wilcock, J., Thuné-Boyle, I., & Iliffe, S. (2017). A comparison of service use by people with dementia in two samples a decade apart. *Dementia: The International Journal of Social Research and Practice, 16*(1), 96-107. | No relevant outcomes |
| Gillani, S. R., Sidhu, M., & Singh, B. M. (2013). An audit of factors affecting access of patients to primary care diabetes services. *Diabetic Medicine, 30*, 190-191. | No relevant outcomes |
| Giordano, V., Cole, C., Jeggo, M., Methuen, C., & McColl, H. (2016). Analysis of inpatient admission and use of mental health act in relation to ethnicity and substance misuse in a cohort of patients with first episode psychosis in London. *Early intervention in psychiatry, 10*, 206. | Not peer-reviewed article |
| Gnanavel, S. (2013). 'Exploring and addressing the unmet healthcare needs of Indian adolescents': Comment. *The British Journal of Psychiatry, 203*(6), 468-468. | Not adults |
| Goldwyn, C. (2013). Many detainees shouldn't be there in the first place. *BMJ, 346*, f2365. | Not peer-reviewed article |
| Jolliffe, D., Cattell, J., Raza, A., & Minoudis, P. (2017). Factors associated with progression in the London pathway project. *Criminal Behaviour and Mental Health, 27*(3), 222-237. | No consideration of ethnicity |
| Keown, P., McBride, O., Twigg, L., Crepaz-Keay, D., Cyhlarova, E., Parsons, H., . . . Weich, S. (2016). Rates of voluntary and compulsory psychiatric in-patient treatment in England: an ecological study investigating associations with deprivation and demographics. *British Journal of Psychiatry, 209*(2), 157-161. | No consideration of ethnicity |
| Khan, F., Chaudhry, I. B., Laganis, C., O'Brian, P., Qureshi, M., Mahadevan, D., & Husain, N. (2012). Ethnic differences in the presentation and characteristics of adolescents in contact with a UK early intervention service. *Early intervention in psychiatry, 6*, 41. | Not peer-reviewed article |
| Kluge, U. P. (2012). Responses to the challenges of immigrant patients in different kinds of health services across Europe. *European Psychiatry, 27*. | Not peer-reviewed article |
| Langford, K. M., Bottle, A., Aylin, P. P., & Ward, H. (2012). Using routine data to monitor inequalities in an acute trust: a retrospective study. *BMC Health Services Research, 12*, 104. | Not mental health |
| Lau, J. S., Adams, S. H., & Irwin, C. E. (2015). Delivery of office-based mental health services to adolescents and young adults. *Journal of Adolescent Health, 56*(2), S98. | Not peer-reviewed article |
| Lepping, P., Masood, B., Flammer, E., & Noorthoorn, E. O. (2016). Comparison of restraint data from four countries. *Social psychiatry and psychiatric epidemiology, 51*(9), 1301-1309. | No consideration of ethnicity |
| Lepresle, A., Mairesse, E., & Chariot, P. (2013). Section 136 and police custody. *The Lancet, 382*(9900), 1248-1248. | No relevant outcomes |
| McGorrian, C., Frazer, K., Daly, L., Moore, R. G., Turner, J., Sweeney, M. R., . . . Kelleher, C. C. (2012). The health care experiences of Travellers compared to the general population: The All-Ireland Traveller Health Study. *Journal of Health Services Research and Policy, 17*(3), 173-180. | Not UK |
| Mills, J. G., Morgan, C., Frissa, S., Verdecchia, M., Stewart, R., Fear, N. T., . . . Hatch, S. L. (2012). Psychotic experiences and help-seeking in a representative community sample. *Schizophrenia Research, 136*, S275. | Not peer-reviewed article |
| Misselbrook, T., Patel, R., Nicholson, T., Cullen, A., & Pollak, T. (2017). Organic psychosis: Using electronic patient records to investigate demographics, etiology, and outcome. *Schizophrenia Bulletin, 43*, S132. | Not peer-reviewed article |
| Morgan, C. (2012). Aesop-10: A 10-year follow-up study of first-episode psychosis-rationale, method, and preliminary findings. *Schizophrenia Research, 136*, S123-S124. | Not peer-reviewed article |
| Morgan, C., Heslin, M., Lappin, J., Croudace, T., Doody, G., Donoghue, K., . . . Dazzan, P. (2013). Ethnicity and the long-term course and outcome of psychosis: Initial findings from AESOP-10. *Schizophrenia Bulletin, 39*, S72. | Duplicate study/sample |
| Morgan, C., Lappin, J., Heslin, M., Croudace, T., Doody, G., Donoghue, K., . . . Dazzan, P. (2014). Ethnicity, social disadvantage and the long-term course and outcome of psychosis. *Schizophrenia Research, 153*, S348. | Duplicate study/sample |
| Mustafa, F. A., Bayatti, Z., & Faruqui, R. A. (2013). Gender differences in referral pathways and admissions to a psychiatric intensive care unit in a county psychiatric hospital in the UK. *International Journal of Social Psychiatry, 59*(2), 188-189. | Relevant data not by ethnicity |
| Newell, S. E., Harries, P., & Ayers, S. (2012). Boredom proneness in a psychiatric inpatient population. *International Journal of Social Psychiatry, 58*(5), 488-495. | No consideration of ethnicity |
| Ngwena, J. (2014). Black and minority ethnic groups (BME) suicide, admission with suicide or self-harm: An inner city study. *Journal of Public Health (Germany), 22*(2), 155-163. | No relevant outcomes |
| O'Donoghue, T., Shine, J., & Orimalade, O. (2014). Characteristics of referrals and admissions to a medium secure ASD unit. *Journal of Intellectual Disabilities & Offending Behaviour, 5*(3), 138-146. | Not psychosis |
| Palmer-Cooper, E., Pollak, T., & Lennox, B. (2016). Antibody mediated psychosis: A clinical and cognitive profile. *Early intervention in psychiatry, 10*, 238. | Not peer-reviewed article |
| Patel, R., Díaz-Caneja, C. M., Valmaggia, L., Byrne, M., Badger, S., Garety, P., . . . Fusar-Poli, P. (2014). Clinical outcomes of people with first episode psychosis presenting to the OASIS prodromal clinic in South London. *Early intervention in psychiatry, 8*, 88. | Not peer-reviewed article |
| Patel, R., Fusar-Poli, P., DíAz-Caneja, C. M., Valmaggia, L., Byrne, M., Badger, S., . . . McGuire, P. (2015). Prodromal services improve clinical outcomes in people who present with an established first episode of psychosis. *Schizophrenia Bulletin, 41*, S150-S151. | Not peer-reviewed article |
| Patel, R., Sheety, H., Boydell, J., Taylor, M., Stewart, R., & McGuire, P. (2014). Factors affecting hospital admission following presentation to mental health services with psychosis. *Early intervention in psychiatry, 8*, 159. | Not peer-reviewed article |
| Patel, R., Shetty, H., Jackson, R., Broadbent, M., Stewart, R., Boydell, J., . . . Taylor, M. (2015). Delays before Diagnosis and Initiation of Treatment in Patients Presenting to Mental Health Services with Bipolar Disorder. *PLoS ONE [Electronic Resource], 10*(5), e0126530. | No relevant outcomes |
| Patel, R., Shetty, H., Jackson, R., Broadbent, M., Stewart, R., Boydell, J., . . . Taylor, M. (2016). Delays to diagnosis and treatment in patients presenting to mental health services with bipolar disorder. *European Psychiatry, 33*, S75. | Duplicate study/sample |
| Patel, R., Wilson, R., Jackson, R., Ball, M., Shetty, H., Broadbent, M., . . . Bhattacharyya, S. (2014). Association of cannabis use with hospital admission and treatment resistance in people with first episode psychosis in South London. *Early intervention in psychiatry, 8*, 59. | Relevant data not by ethnicity |
| Patel, R., Wilson, R., Jackson, R., Ball, M., Shetty, H., Broadbent, M., . . . Bhattacharyya, S. (2015). Cannabis use and treatment resistance in first episode psychosis: A natural language processing study. *The Lancet, 385*, S79. | Duplicate study/sample |
| Payne, R. A., Abel, G. A., Guthrie, B., & Mercer, S. W. (2013). The effect of physical multimorbidity, mental health conditions and socioeconomic deprivation on unplanned admissions to hospital: A retrospective cohort study. *CMAJ, 185*(5), E221-E228. | No consideration of ethnicity |
| Poole, R., Pearsall, A., & Ryan, T. (2014). Delayed discharges in an urban in-patient mental health service in England. *Psychiatrist, 38*(2), 66-70. | No consideration of ethnicity |
| Roberts, A. J., Senior, J., Hayes, A. J., Stevenson, C., & Shaw, J. J. (2012). An independent evaluation of the Department of Health's procedure for the transfer of prisoners to hospital under the Mental Health Act 1983. *Journal of Forensic Psychiatry and Psychology, 23*(2), 217-236. | No consideration of ethnicity |
| Sanchez-Cao, E., Kramer, T., & Hodes, M. (2013). Psychological distress and mental health service contact of unaccompanied asylum-seeking children. *Child: Care, Health & Development, 39*(5), 651-659. | No relevant outcomes |
| Sheikh, S., & Furnham, A. (2012). The relationship between somatic expression, psychological distress and GP consultation in two cultural groups. *Counselling Psychology Quarterly, 25*(4), 389-402. | Not psychosis |
| Siva, N. (2013). Time in detention. *The Lancet, 381*(9860), 15-16. | No relevant outcomes |
| Sonuga-Barke, E. J. S., Kennedy, M., Kumsta, R., Knights, N., Golm, D., Rutter, M., . . . Kreppner, J. (2017). Child-to-adult neurodevelopmental and mental health trajectories after early life deprivation: the young adult follow-up of the longitudinal English and Romanian Adoptees study. *The Lancet, 389*(10078), 1539-1548. | No relevant outcomes |
| Spencer, S. J., Gilluley, P., & Hillier, B. (2013). Foreign national mentally disordered offenders' care pathways through UK secure services. *Psychiatrist, 37*(10), 331-335. | No relevant outcomes |
| Tapfumaneyi, A., Johnson, S., Joyce, J., Major, B., Lawrence, J., Mann, F., . . . MiData, C. (2015). Predictors of vocational activity over the first year in inner-city early intervention in psychosis services. *Early intervention in psychiatry, 9*(6), 447-458. | No relevant outcomes |
| Topal, K., Eser, E., Sanberk, I., Bayliss, E., & Saatci, E. (2012). Challenges in access to health services and its impact on quality of life: a randomised population-based survey within Turkish speaking immigrants in London. *Health & Quality of Life Outcomes, 10*, 11. | Not mental health |
| Tridente, A., Chick, A., Keep, S., Furmanova, S., Webber, S., & Bryden, D. (2012). Functional status as a predictor of admission to critical care in acutely unwell patients. *Intensive Care Medicine, 38*, S119-S120. | Not peer-reviewed article |
| Tridente, A., Chick, A., Keep, S., Furmanova, S., Webber, S., & Bryden, D. C. (2012). Factors affecting critical care  admission to a UK university hospital. *Critical Care, 16*, S180-S181. | Not peer-reviewed article |
| Tulloch, A. D., Fearon, P., & David, A. S. (2012). Timing, prevalence, determinants and outcomes of homelessness among patients admitted to acute psychiatric wards. *Social psychiatry and psychiatric epidemiology, 47*(7), 1181-1191. | Relevant data not by ethnicity |
| Tulloch, A. D., Fearon, P., & David, A. S. (2012). 'Timing, prevalence, determinants and outcomes of homelessness among patients admitted to acute psychiatric wards': Erratum. *Social psychiatry and psychiatric epidemiology, 47*(7), 1193-1193. | Relevant data not by ethnicity |
| Valmaggia, L. R., Byrne, M., Day, F., Broome, M. R., Johns, L., Howes, O., . . . McGuire, P. K. (2015). Duration of untreated psychosis and need for admission in patients who engage with mental health services in the prodromal phase. *British Journal of Psychiatry, 207*(2), 130-134. | Relevant data not by ethnicity |
| Van Mechelen, C., Riaz, H., & Uzair, F. (2012). Understanding and interpreting trends of care within ethnic diversity. *European Psychiatry, 27*. | Not peer-reviewed article |
| Von Reventlow, H. G., Krüger-Özgürdal, S., Ruhrmann, S., Schultze-Lutter, F., Heinz, A., Patterson, P., . . . Juckel, G. (2014). Pathways to care in subjects at high risk for psychotic disorders - A European perspective. *Schizophrenia Research, 152*(2), 400-407. | Relevant data not by ethnicity |
| Von Reventlow, H. G., Patterson, P., Ruhrmann, S., Linszen, D., Schultze-Lutter, F., Salokangas, R. K. R., . . . Juckel, G. (2012). Pathways to care of UHR patients across four European regions. *Early intervention in psychiatry, 6*, 36. | Not peer-reviewed article |
| Vostanis, P., Svirydzenka, N., Dugard, P., Singh, S., & Dogra, N. (2013). Mental health service use by adolescents of Indian and White origin. *Archives of Disease in Childhood, 98*(10), 764-767. | Not adults |
| Warwick Medical School (2013). Patient ethnicity has no impact on likelihood of mental health detention, study finds. *Mental Health Today*, 6-6. | Not peer-reviewed article |
| Watson, J., & Daley, S. (2015). The use of section 135(1) of the Mental Health Act in a London borough. *Mental Health Review Journal, 20*(3), 133-143. | No relevant outcomes |
| Winsper, C., Singh, S. P., Marwaha, S., Amos, T., Lester, H., Everard, L., . . . Birchwood, M. (2013). Pathways to violent behavior during first-episode psychosis: A report from the UK national EDEN study. *JAMA Psychiatry, 70*(12), 1287-1293. | No consideration of ethnicity |
| Yassaee, A. A., Hargreaves, D. S., Chester, K., Lamb, S., Hagell, A., & Brooks, F. M. (2017). Experience of Primary Care Services Among Early Adolescents in England and Association With Health Outcomes. *Journal of Adolescent Health, 60*(4), 388-394. | Not adults |
| Zimbron, J., Ruiz de Azua, S., , K., aker, G. M., , G., amaneni, P. K., . . . Perez, J. (2013). Clinical and sociodemographic comparison of people at high-risk for psychosis and with first-episode psychosis. *Acta Psychiatrica Scandinavica, 127*(3), 210-216. | Relevant data not by ethnicity |
