# Supplementary material for: Ethnic inequalities and pathways to care in psychosis in England: a systematic review and meta-analysis
Source: BMC Med. 2018 Dec 12;16:223. doi: 10.1186/s12916-018-1201-9 (PMC6290527; doi:10.1186/s12916-018-1201-9)
Supplement: Supplementary file 4 — Full summary and quality scores of included primary studies (used in meta-analyses). (DOCX 51 kb) [file 12916_2018_1201_MOESM4_ESM.docx]

**Additional file 4:**

**Full summary and quality scores of included primary studies (used in meta-analyses)**

| STUDIES from PREVIOUS REVIEWS/META-ANALYSES (N=29)   \|  \|  \|  \|  \|  \|  \|  \|  \| \| --- \| --- \| --- \| --- \| --- \| --- \| --- \| --- \| \| Study \| Ethnicity measure(s) \| Ethnicity categories (*n*) \| GP^*^ *n*, % \| Detention *n*, % (civil/forensic/mixed^†^) \| Police or CJS^‡^ *n*, % \| DUP^§^ (days), median (IQR^¶^) \| Quality assessment  (max score = 11)^\|\|^ \| | | | | | | | | | | | | | |
| --- | --- | --- | --- | --- | --- | --- | --- | --- | --- | --- | --- | --- | --- | --- | --- | --- | --- | --- | --- | --- | --- | --- | --- | --- | --- | --- | --- | --- | --- |
|  |  |  | | |  | |  | |  | |  | |  |
| Bebbington et al. 1994 | Not reported | White (190)  Black Caribbean (49) | | |  | | 38, 20% (civil)  22, 45% (civil) | |  | |  | | Sample (source and size) = 2  Adjustment (confounders) = 2  Ethnicity categorisation (quality) = 0  Ethnicity categorisation (analysis) = 0  **Total = 4 (moderate quality)** |
| Banerjee et al. 1995 | Not reported | White European (804)  Black Caribbean (375)  Other (50) | | |  | | 22, 3% (forensic)  27, 7% (forensic)  4, 8% (forensic) | |  | |  | | Sample (source and size) = 1  Adjustment (confounders) = 1  Ethnicity categorisation (quality) = 0  Ethnicity categorisation (analysis) = 0  **Total = 2 (low quality)** |
| Bhui et al. 1998 | By place of birth and Census categorisation | White (184)  Black African (16)  Black British (12)  Black Caribbean (26)  Asian/Other (18) | | |  | | 29, 16% (forensic)  6, 38% (forensic)  7, 59% (forensic)  7, 27% (forensic)  7, 39% (forensic) | |  | |  | | Sample (source and size) = 1  Adjustment (confounders) = 4  Ethnicity categorisation (quality) = 1  Ethnicity categorisation (analysis) = 2  **Total = 8 (high quality)** |
| Birchwood et al. 1992 | Third-party reports | White British (74)  Black Caribbean British (50)  Asian British (30)  Irish (5)  Other (10) | | |  | | 19, 26% (civil)  16, 32% (civil)  4, 13% (civil) | | 5, 13% (police)  10, 30% (police)  2, 12% (police) | |  | | Sample (source and size) = 2  Adjustment (confounders) = 1  Ethnicity categorisation (quality) = 0  Ethnicity categorisation (analysis) = 2  **Total = 5 (moderate quality)** |
| Brunet 2003 | Third-party reports | White (16)  Black (36)  Asian (28)  Other (8) | | | 12, 80%  21, 66%  19, 79%  5, 71% | | 5, 31% (civil)  16, 44% (civil)  8, 29% (civil)  1, 13% (civil) | |  | | 711, 884 (n=15)**  209, 319**  195, 312 (n=26)** | | Sample (source and size) = 1  Adjustment (confounders) = 1  Ethnicity categorisation (quality) = 0  Ethnicity categorisation (analysis) = 0  **Total = 2 (low quality)** |
| Burnett et al. 1999 | By place of birth | White (38)  Black Caribbean (38)  Asian (24) | | | 19, 50%  14, 37%  13, 54% | | 108, 14% (mixed)  43, 19% (mixed)  - | | 6, 16% (police/CJS)  10, 26% (police/CJS)  1, 4% (police/CJS) | |  | | Sample (source and size) = 1  Adjustment (confounders) = 3  Ethnicity categorisation (quality) = 1  Ethnicity categorisation (analysis) = 1  **Total = 6 (moderate quality)** |
| Callan 1996 | By place of birth | White British-born (169)  Black Caribbean (200) | | | 80, 48%  78, 40% | | 45, 27% (civil)  107, 54% (civil) | | 24, 14% (police)  49, 25% (police) | |  | | Sample (source and size) = 2  Adjustment (confounders) = 2  Ethnicity categorisation (quality) = 1  Ethnicity categorisation (analysis) = 2  **Total = 7 (moderate quality)** |
| Cole et al. 1995 | Self-reported/  Census categorisation | White (39)  Black (38)  Asian and Other (16) | | | 27, 69%  26, 68%  13, 81% | | 11, 28% (civil)  15, 39% (civil)  3, 19% (civil) | | 15, 38% (police)  17, 45% (police)  7, 44% (police) | |  | | Sample (source and size) = 1  Adjustment (confounders) = 4  Ethnicity categorisation (quality) = 1  Ethnicity categorisation (analysis) = 0  **Total = 6 (moderate quality)** |
| Commander et al. 1999 | Self-reported/  Census categorisation | White (40)  Black (40)  Asian (40) | | | 5, 13%  12, 30%  18, 45% | | 11, 28% (civil)  27, 68% (civil)  23, 58% (civil) | | 4, 10% (police)  24, 60% (police)  16, 40% (police) | |  | | Sample (source and size) = 2  Adjustment (confounders) = 1  Ethnicity categorisation (quality) = 1  Ethnicity categorisation (analysis) = 0  **Total = 4 (moderate quality)** |
| Crowley and Simmons 1992 | Third-party reports | White (75)  Black Caribbean (49)  Asian (28) | | |  | | 8, 11% (civil)  21, 43% (civil)  4, 14% (civil) | |  | |  | | Sample (source and size) = 1  Adjustment (confounders) = 1  Ethnicity categorisation (quality) = 0  Ethnicity categorisation (analysis) = 1  **Total = 3 (low quality)** |
| Davies et al. 1996 | By place of birth and Census categorisation | White UK-born (207)  White non-UK-born (36)  Black African UK-born (6)  Black African non-UK-born (21)  Black Caribbean UK-born (58)  Black Caribbean non-UK-born (54)  Other (15) | | |  | | 85, 41% (mixed)  18, 50% (mixed)  5, 83% (mixed)  14, 67% (mixed)  43, 74% (mixed)  36, 67% (mixed)  7, 47% (mixed) | |  | |  | | Sample (source and size) = 2  Adjustment (confounders) = 3  Ethnicity categorisation (quality) = 1  Ethnicity categorisation (analysis) = 2  **Total = 8 (high quality)** |
| Drake et al. 2000 | Self-reported | White (216)  Black Caribbean (19)  Other (13) | | |  | |  | |  | | 84, 35-252  63, 42-196  70, 42-280 | | Sample (source and size) = 1  Adjustment (confounders) = 3  Ethnicity categorisation (quality) = 1  Ethnicity categorisation (analysis) = 1  **Total = 6 (moderate quality)** |
| Goater et al. 1999 | Self-reported/  Census categorisation | White (68)  Black (71)  Other (15) | | |  | | 19, 28% (mixed)  29, 41% (mixed)  6, 40% (mixed) | |  | |  | | Sample (source and size) = 2  Adjustment (confounders) = 3  Ethnicity categorisation (quality) = 1  Ethnicity categorisation (analysis) = 0  **Total = 6 (moderate quality)** |
| Harrison et al. 1989 | Not reported | General Population (89)  Black Caribbean (42) | | | 67, 77%  25, 63% | | 19, 21% (civil)  19, 45% (civil) | | 6, 7% (police)  8, 20% (police) | |  | | Sample (source and size) = 2  Adjustment (confounders) = 2  Ethnicity categorisation (quality) = 0  Ethnicity categorisation (analysis) = 0  **Total = 4 (moderate quality)** |
| Ineichen et al. 1984 | Third-party reports | White UK (193)  Other (White) (9)  Black Caribbean (43)  Other (Non-White) (19) | | |  | | 54, 28% (civil)  3, 33% (civil)  27, 63% (civil)  5, 26% (civil) | |  | |  | | Sample (source and size) = 2  Adjustment (confounders) = 1  Ethnicity categorisation (quality) = 0  Ethnicity categorisation (analysis) = 1  **Total = 4 (moderate quality)** |
| Johnson et al. 1998 | Not reported | White (173)  Black African (15)  Black Caribbean (70)  Other (14) | | |  | | 26, 15% (civil)  4, 27% (civil)  23, 33% (civil)  3, 21% (civil) | |  | |  | | Sample (source and size) = 1  Adjustment (confounders) = 3  Ethnicity categorisation (quality) = 0  Ethnicity categorisation (analysis) = 1  **Total = 5 (moderate quality)** |
| Koffman et al. 1997 | Third-party reports | White (2,978)  Black (631)  Asian (160) | | |  | | 911, 31% (civil)  401, 64% (civil)  62, 39% (civil) | |  | |  | | Sample (source and size) = 3  Adjustment (confounders) = 2  Ethnicity categorisation (quality) = 0  Ethnicity categorisation (analysis) = 0  **Total = 5 (moderate quality)** |
| Lloyd and Moodley 1992 | Third-party reports | White (101)  Black (37) | | |  | | 30, 30% (civil)  25, 68% (civil) | |  | |  | | Sample (source and size) = 2  Adjustment (confounders) = 3  Ethnicity categorisation (quality) = 0  Ethnicity categorisation (analysis) = 0  **Total = 5 (moderate quality)** |
| McKenzie et al. 1995 | By place of birth | White British (58)  Black Caribbean (53) | | |  | | 16, 43% (civil)  33, 83% (civil) | | 2, 3% (CJS)  12, 23% (CJS) | |  | | Sample (source and size) = 2  Adjustment (confounders) = 3  Ethnicity categorisation (quality) = 1  Ethnicity categorisation (analysis) = 2  **Total = 8 (high quality)** |
| Moodley and Perkins 1991 | Not reported | White (25)  Black Caribbean (22) | | |  | | 6, 24% (civil)  13, 59% (civil) | |  | |  | | Sample (source and size) = 1  Adjustment (confounders) = 1  Ethnicity categorisation (quality) = 0  Ethnicity categorisation (analysis) = 0  **Total = 2 (low quality)** |
| Moodley and Thornicroft 1988 | Third-party reports | White (295)  Black Caribbean (47) | | |  | | 65, 22% (civil)  26, 55% (civil) | | 0 (police)  4, 31% (police) | |  | | Sample (source and size) = 1  Adjustment (confounders) = 1  Ethnicity categorisation (quality) = 0  Ethnicity categorisation (analysis) = 1  **Total = 3 (low quality)** |
| Morgan et al. 2005 | Self-reported | White British (237)  Other White (33)  Black African (64)  Black Caribbean (128) | | | 99, 42%  15, 45%  14, 22%  32, 25% | | 64, 27% (civil)  10, 30% (civil)  35, 55% (civil)  66, 52% (civil) | | 34, 14% (CJS)  6, 18% (CJS)  21, 33% (CJS)  38, 30% (CJS) | |  | | Sample (source and size) = 2  Adjustment (confounders) = 4  Ethnicity categorisation (quality) = 1  Ethnicity categorisation (analysis) = 2  **Total = 9 (high quality)** |
| Morgan et al. 2006 | Self-reported | White British (217)  Black Caribbean (129)  Black African (68) | | |  | |  | |  | | 56, 14-273  84, 28-539  56, 7-147 | | Sample (source and size) = 2  Adjustment (confounders) = 3  Ethnicity categorisation (quality) = 1  Ethnicity categorisation (analysis) = 2  **Total = 8 (high quality)** |
| Owens et al. 1991 | Third-party reports | Non-Black Caribbean (155)  Black Caribbean (120) | | |  | | 224, 10% (civil)  55, 46% (civil) | | 9, 20% (police)  24, 57% (police) | |  | | Sample (source and size) = 2  Adjustment (confounders) = 2  Ethnicity categorisation (quality) = 0  Ethnicity categorisation (analysis) = 0  **Total = 4 (moderate quality)** |
| Parkman et al. 1997 | By place of birth and Census categorisation | White UK-born (94)  White non-UK-born (17)  Black Caribbean UK-born (22)  Black Caribbean non-UK-born (20) | | |  | | 46, 49% (mixed)  10, 59% (mixed)  18, 82% (mixed)  16, 80% (mixed) | |  | |  | | Sample (source and size) = 1  Adjustment (confounders) = 3  Ethnicity categorisation (quality) = 1  Ethnicity categorisation (analysis) = 2  **Total = 7 (moderate quality)** |
| Patrick et al. 1989 | Not reported | White (34)  Black (26) | | |  | | 10, 29% (civil)  15, 58% (civil) | |  | |  | | Sample (source and size) = 1  Adjustment (confounders) = 2  Ethnicity categorisation (quality) = 0  Ethnicity categorisation (analysis) = 0  **Total = 3 (low quality)** |
| Singh et al. 1998 | Census categorisation and third-party reports | White (352)  Black Caribbean (44) | | |  | | 66, 19% (civil)  19, 43% (civil) | |  | |  | | Sample (source and size) = 2  Adjustment (confounders) = 4  Ethnicity categorisation (quality) = 1  Ethnicity categorisation (analysis) = 1  **Total = 8 (high quality)** |
| Takei et al. 1998 | Not reported | White (49)  Black Caribbean (32) | | |  | | 13, 27% (civil)  21, 66% (civil) | |  | |  | | Sample (source and size) = 2  Adjustment (confounders) = 3  Ethnicity categorisation (quality) = 0  Ethnicity categorisation (analysis) = 0  **Total = 5 (moderate quality)** |
| Thomas et al. 1993 | Third-party reports | UK-born European 16-29 years (452)  UK-born European 30-44 years (439)  UK-born European 45-pens. age (374)  Black Caribbean 16-29 years, 1^st^ gen. (27)  Black Caribbean 16-29 years, 2^nd^ gen. (67)  Black Caribbean 30-44 years (50)  Black Caribbean 45-pens. age (49)  Asian 16-29 years, 1^st^ generation (16)  Asian 16-29 years, 2^nd^ generation (14)  Asian 30-44 years (28)  Asian 45-pensionable age (18) | | |  | | 77, 17% (civil)  59, 13% (civil)  52, 14% (civil)  13, 48% (civil)  26, 39% (civil)  30, 60% (civil)  28, 57% (civil)  8, 50% (civil)  5, 36% (civil)  10, 36% (civil)  7, 39% (civil) | | 22, 5% (police)  14, 3% (police)  12, 3% (police)  2, 7% (police)  13, 19% (police)  5, 10% (police)  3, 6% (police)  0 (police)  1, 7% (police)  1, 4% (police)  0 (police) | |  | | Sample (source and size) = 0  Adjustment (confounders) = 2  Ethnicity categorisation (quality) = 0  Ethnicity categorisation (analysis) = 1  **Total = 3 (low quality)** |
|  |  |  | | |  | |  | |  | |  | |  |
| STUDIES IDENTIFIED IN SEARCH FOR PRIMARY LITERATURE 2012-2017 (n=11) | | | | | | | | | | | | | |
|  |  | |  |  | |  | |  | |  | |  | |
| Study | **Ethnicity measure(s)** | | **Ethnicity categories (*n*)** | **GP^*^ *n*, %** | | **Detention *n*, % (civil/forensic/mixed^†^)** | | **Police or CJS^‡^ *n*, %** | | **DUP^§^ (days), median (IQR**^¶^**)** | | **Quality assessment**  **(max score = 11)^\|\|^** | |
|  |  | |  |  | |  | |  | |  | |  | |
| Ajnakina et al. 2017 | Census categorisation | | White British (62)  Black African (63)  Black Caribbean (50) |  | | 33, 53% (civil)  51, 81% (civil)  31, 62% (civil) | | 27, 44% (police)  48, 77% (police)  25, 50% (police) | |  | | Sample (source and size) = 2  Adjustment (confounders) = 2  Ethnicity categorisation (quality) = 1  Ethnicity categorisation (analysis) = 2  **Total = 7 (moderate quality)** | |
| Bhui et al. 2014 | Self-reported | | White (177)  Black (160)  Indian sub-continent (114)  Other (29) | 65, 37%  43, 27%  54, 47%  17, 24% | |  | | 37, 21% (CJS)  60, 38% (CJS)  13, 11% (CJS)  12, 41% (CJS) | | 122, 61-305  92, 31-153  122, 61-365  122, 61-365 | | Sample (source and size) = 2  Adjustment (confounders) = 5  Ethnicity categorisation (quality) = 1  Ethnicity categorisation (analysis) = 1  **Total = 9 (high quality)** | |
| Bhui et al. 2015 | Census categorisation | | White British (23)  White Other (14)  Black African (28)  Black Caribbean (31)  Black Other (1)  Bangladeshi (4)  Indian (4)  Pakistani (3)  Other (14) |  | |  | | N/A  N/A  N/A  N/A  N/A  N/A  N/A  N/A  N/A | |  | | Sample (source and size) = 1  Adjustment (confounders) = 5  Ethnicity categorisation (quality) = 1  Ethnicity categorisation (analysis) = 2  **Total = 9 (high quality)** | |
| Gajwani et al. 2016 | Self-reported | | White British/Other (437)  Black African (62)  Black Caribbean (120)  Asian Bangladeshi (16)  Asian Indian (47)  Asian Pakistani (125) |  | | 306, 70% (mixed)  38, 61% (mixed)  87, 73% (mixed)  15, 94% (mixed)  30, 64% (mixed)  85, 68% (mixed) | |  | |  | | Sample (source and size) = 3  Adjustment (confounders) = 3  Ethnicity categorisation (quality) = 1  Ethnicity categorisation (analysis) = 2  **Total = 9 (high quality)** | |
| Ghali et al. 2013 | Census categorisation | | White British (183)  White Other (103)  Black African (136)  Black British (152)  Black Caribbean (27)  South Asian (80) | 99, 54%  40, 39%  60, 44%  63, 42%  7, 26%  41, 53% | |  | | 31, 17% (CJS)  23, 23% (CJS)  48, 36% (CJS)  41, 27% (CJS)  9, 33% (CJS)  12, 15% (CJS) | | 113, 345 (n=134)  72, 198 (n=84)  57, 155 (n=110)  98, 182 (n=126)  55, 236 (n=21)  60, 164 (n=60) | | Sample (source and size) = 3  Adjustment (confounders) = 5  Ethnicity categorisation (quality) = 1  Ethnicity categorisation (analysis) = 2  **Total = 11 (high quality)** | |
| Lawlor et al. 2012 | Census categorisation | | White British (146)  White Other (45)  Black African (41)  Black Caribbean (26)  Black Other (29) | 23, 16%  6, 13%  4, 10%  1, 4%  1, 3% | | 19, 13% (civil)  16, 36% (civil)  20, 49% (civil)  11, 42% (civil)  13, 45% (civil) | | 25, 17% (police/CJS)  15, 33% (police/CJS)  17, 41% (police/CJS)  8, 31% (police/CJS)  14, 48% (police/CJS) | |  | | Sample (source and size) = 2  Adjustment (confounders) = 5  Ethnicity categorisation (quality) = 1  Ethnicity categorisation (analysis) = 2  **Total = 10 (high quality)** | |
| Mann et al. 2014 | Self-reported/  Census categorisation | | White British (158)  White Other (93)  Black African (188)  Black British (55)  Black Caribbean (78)  Mixed Black/White (36)  South Asian (37)  Asian Other (29) | 61, 50%  36, 42%  49, 34%  9, 28%  23, 38%  14, 48%  18, 56%  7, 30% | | 53, 35% (civil)  32, 37% (civil)  105, 60% (civil)  27, 53% (civil)  32, 46% (civil)  14, 41% (civil)  15, 44% (civil)  10, 37% (civil) | | 11, 8% (CJS)  13, 14% (CJS)  89, 15% (CJS)  11, 16% (CJS)  40, 25% (CJS)  4, 13% (CJS)  3, 12% (CJS)  3, 6% (CJS) | |  | | Sample (source and size) = 3  Adjustment (confounders) = 3  Ethnicity categorisation (quality) = 1  Ethnicity categorisation (analysis) = 2  **Total = 9 (high quality)** | |
| Morgan et al. 2017 | Self-reported/  Census categorisation | | White British (159)  Black African (44)  Black Caribbean (107) |  | | 89, 58% (civil)  36, 84% (civil)  84, 79% (civil) | | 58, 36% (police)  28, 64% (police)  66, 62% (police) | |  | | Sample (source and size) = 2  Adjustment (confounders) = 3  Ethnicity categorisation (quality) = 1  Ethnicity categorisation (analysis) = 2  **Total = 8 (high quality)** | |
| Singh et al. 2014 | Third-party reports | | White (2,587)  Black (811)  Asian (430)  Other (359) |  | | 1,668, 64% (civil)  577, 71% (civil)  273, 63% (civil)  255, 71% (civil) | |  | |  | | Sample (source and size) = 3  Adjustment (confounders) = 4  Ethnicity categorisation (quality) = 0  Ethnicity categorisation (analysis) = 0  **Total = 7 (moderate quality)** | |
| Singh et al. 2015 | Self-reported/  Census categorisation | | White (45)  Black (35)  Asian (43) | N/A  N/A  N/A | | N/A  N/A  N/A | | N/A  N/A  N/A | | 685 (838)**  751 (1,078)**  627 (657)** | | Sample (source and size) = 2  Adjustment (confounders) = 4  Ethnicity categorisation (quality) = 1  Ethnicity categorisation (analysis) = 0  **Total = 7 (moderate quality)** | |
| Weich et al. 2017 | Not reported | | White (997,169)  Black or Black British (39,249)  Asian or Asian British (46,544)  Mixed (13,781)  Other Ethnicity (22,053) |  | | 32,130, 3% (civil)  4,965, 13% (civil)  2,771, 6% (civil)  1,027, 7% (civil)  1,052, 5% (civil) | |  | |  | | Sample (source and size) = 0  Adjustment (confounders) = 2  Ethnicity categorisation (quality) = 0  Ethnicity categorisation (analysis) = 0  **Total = 2 (low quality)** | |

^*^ = GP stands for general practitioner involvement.

^†^ = While ’forensic population’ refers to samples who are currently imprisoned or otherwise remanded through the criminal justice system, ‘civil population’ refers to samples who do not currently have these punitive measures imposed upon them. ‘Mixed population’ refers to studies that include a mixture of both civil and forensic populations (as defined above) in their samples.

^‡^ = CJS stands for criminal justice system involvement.

§ = DUP stands for the duration of untreated psychosis.

¶ = IQR stands for the inter-quartile range.

^||^ = The scoring system used to rate primary studies is replicated from Bhui et al.^3^ From a maximum of 11 points, primary studies that received a total of 0-3 points were ranked as ‘low’ quality, 4-7 points ‘moderate’ quality and 8-11 points ‘high’ quality.

^**^ = These numbers refer to the mean days and standard deviations, as the medians and IQRs were not available to use as summary statistics in the table for these studies.
