# Supplementary material for: Ethnic inequalities and pathways to care in psychosis in England: a systematic review and meta-analysis
Source: BMC Med. 2018 Dec 12;16:223. doi: 10.1186/s12916-018-1201-9 (PMC6290527; doi:10.1186/s12916-018-1201-9)
Supplement: Supplementary file 5 — Relevant sections of the Mental Health Act (1983, amended in 2007). (DOCX 22 kb) [file 12916_2018_1201_MOESM5_ESM.docx]

**Additional file 5:**

**Relevant sections of the Mental Health Act (1983, amended in 2007)**

| **Civil sections** | |
| --- | --- |
| *Section* | *Definition* |
| Section 2  (Admission for assessment) | Concerns the power to compulsory admit someone for up to 28 days for mental health assessment and is instigated based on an applicant from an approved mental health professional (AMHP) or the patient’s nearest relative and must be supported by two medical recommendations, at least one of which from a doctor approved with special expertise in the diagnosis or treatment of mental disorder. |
| Section 3  (Admission for treatment) | Concerns the power to compulsory admit someone for up to 6 months for treatment. The treatment can then be renewed for another 6 months, with any subsequent renewals at 12-monthly intervals. Treatment under this section is also instigated based on an applicant from an AMHP or the patient’s nearest relative, supported by two medical practitioners’ respective recommendations. |
| Section 4  (Emergency assessment) | As this involves an emergency procedure, only one medical recommendation is required to sanction assessment under this section while the conditions for assessment under Section 2 (see above) otherwise will also need to be fulfilled. People may be detained for up to 72 hours under Section 4, but if a second medical recommendation is also provided within this time the assessment may be extended to a 28-day period. |
| Section 5  (Holding powers) | This section refers to the holding powers of registered mental health or learning disability nurses to restrain inpatients who already receive treatment for mental illness from leaving their hospital and hold them for up to 6 hours. Within this period, the doctor or approved clinician responsible for the patient’s treatment or their deputy is expected to see the patient and deem whether it would also be necessary to write a report to the managers. |
| **Forensic sections** | |
| *Section* | *Definition* |
| Section 35  (Remand to hospital for mental health report) | This section bestows power to the Crown Court or a magistrates’ court to remand an accused person to a hospital in order that a report on their mental condition is furnished. The decision to remand is based on written or oral evidence by one registered medical practitioner relating to the accused person’s suspected mental illness and the court’s view that remanding the person on bail would compromise the ability to report on their mental condition. The period of remand in hospital should not extend beyond 28 days at a time, or for more than 12 weeks altogether. |
| Section 37  (Court order of hospital admission or guardianship) | Refers to conviction before the Crown Court or a magistrates’ court of an offence punishable with imprisonment, in which the court may authorise a person’s admission to and detention in a hospital or place them under the guardianship of a local social services authority or someone approved by a local social services authority. The decision is based on the written or oral evidence by two registered medical practitioners relating to the person’s suspected mental illness. |
| Section 41  (Restriction of discharge from hospital) | Referred to as ‘a restriction order’, higher courts may order that an offender is restricted their discharge from hospital. This section is applied to protect the public in cases in which there are reasons to believe that further offences may be committed if the offender is set at large. The section cannot be used unless supporting oral evidence has been given in court by at least one of the medical practitioners that provided evidence of the person’s suspected mental illness under Section 37 (see above). |
| **Police sections** | |
| *Section* | *Definition* |
| Section 135  (Warrant to search and remove) | Based on the information from an AMHP that a person with suspected mental illness has been or is being mistreated or unable to care for themselves, a warrant may be issued granting any constable the authority to enter the relevant premises (by force if deemed necessary) and potentially remove the person to a place of safety for up to 24 hours. After this period, the detention may be extended by a registered medical practitioner for a further 12 hours. |
| Section 136  (Removal without a warrant) | This section does not require a warrant and refers to situations where a constable deems that a person suffering from suspected mental illness is in immediate need of either care or control from themselves or in the interest of public protection. In these circumstances, the constable may remove the person to a place of safety or (if already there) keep the person at the place of safety or remove the person to a different place of safety for up to 24 hours. After this period, the detention may be extended by a registered medical practitioner for a further 12 hours. |

**References**

Mental Health Act 1983, c. 20. <http://www.legislation.gov.uk/ukpga/1983/20>. Accessed 28 July 2018.

Williamson T, Lawton-Smith S. Mental health and the law. In: Norman I, Ryrie I, editors. The art and science of mental health nursing: principles and practice (third edition). London: McGraw-Hill Education; 2013. p. 137-54.
